# A Highly Efficient Human Pluripotent Stem Cell Microglia Model Displays a Neuronal-Co-culture-Specific Expression Profile and Inflammatory Response

**DOI:** 10.1016/j.stemcr.2017.05.017

**Published:** 2017-06-09

**Authors:** Walther Haenseler, Stephen N. Sansom, Julian Buchrieser, Sarah E. Newey, Craig S. Moore, Francesca J. Nicholls, Satyan Chintawar, Christian Schnell, Jack P. Antel, Nicholas D. Allen, M. Zameel Cader, Richard Wade-Martins, William S. James, Sally A. Cowley

**Affiliations:** 1 Sir William Dunn School of Pathology, University of Oxford, South Parks Road, Oxford OX1 3RE, UK; 2 Kennedy Institute of Rheumatology, University of Oxford, Roosevelt Drive, Headington, Oxford OX3 7FY, UK; 3 Department of Pharmacology, University of Oxford, Oxford OX1 3QT, UK; 4 Division of BioMedical Sciences, Faculty of Medicine, Memorial University of Newfoundland, St. John's, NL A1B 3V6, Canada; 5 Department of Psychiatry, University of Oxford, Warneford Hospital, Oxford OX3 7JX, UK; 6 Weatherall Institute of Molecular Medicine, University of Oxford, Oxford OX3 9DS, UK; 7 School of Biosciences, College of Biomedical and Life Sciences, Cardiff University, Cardiff CF10 3AT, UK; 8 Montreal Neurological Institute, McGill University, Montreal, QC H3A 2B4, Canada; 9 Department of Physiology, Anatomy and Genetics, University of Oxford, South Parks Road, Oxford OX1 3QX, UK; 10 Oxford Parkinson's Disease Centre, University of Oxford, South Parks Road, Oxford OX1 3QX, UK

**Keywords:** human, induced pluripotent stem cell, iPSC, macrophage, microglia, cortical neurons, neuroinflammation, neurodegeneration, Alzheimer's disease, Parkinson's disease

## Abstract

Microglia are increasingly implicated in brain pathology, particularly neurodegenerative disease, with many genes implicated in Alzheimer's, Parkinson's, and motor neuron disease expressed in microglia. There is, therefore, a need for authentic, efficient in vitro models to study human microglial pathological mechanisms. Microglia originate from the yolk sac as *MYB*-independent macrophages, migrating into the developing brain to complete differentiation. Here, we recapitulate microglial ontogeny by highly efficient differentiation of embryonic *MYB*-independent iPSC-derived macrophages then co-culture them with iPSC-derived cortical neurons. Co-cultures retain neuronal maturity and functionality for many weeks. Co-culture microglia express key microglia-specific markers and neurodegenerative disease-relevant genes, develop highly dynamic ramifications, and are phagocytic. Upon activation they become more ameboid, releasing multiple microglia-relevant cytokines. Importantly, co-culture microglia downregulate pathogen-response pathways, upregulate homeostatic function pathways, and promote a more anti-inflammatory and pro-remodeling cytokine response than corresponding monocultures, demonstrating that co-cultures are preferable for modeling authentic microglial physiology.

## Highlights

• iPSC microglia co-culture model with iPSC cortical neurons
• Expression of microglia markers in co-culture
• Co-culture promotes microglial ramification and motility
• Co-culture-specific cytokine release profiles

## Graphical Abstract

**Figure:**
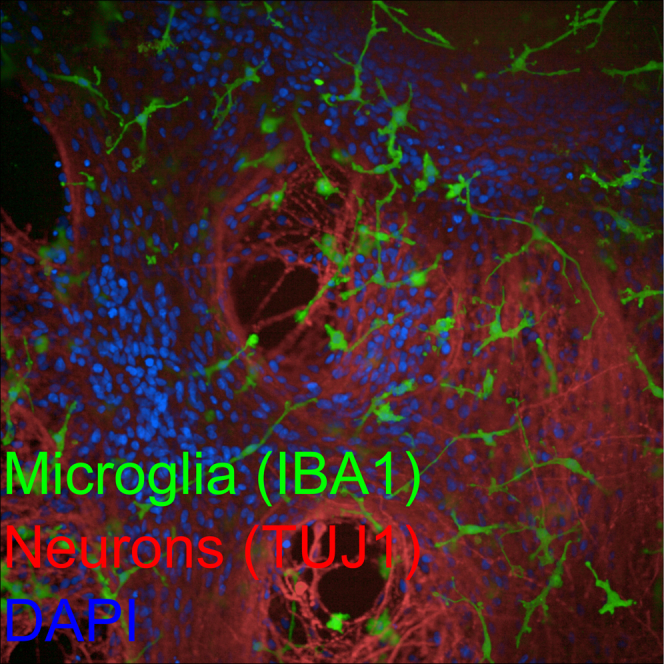

## Teaser

Microglia are implicated in neurodegenerative disease, and authentic human in vitro models are needed. The iPSC microglia model described here recapitulates microglia ontogeny by differentiating *MYB*-independent embryonic iPSC macrophages, then co-culturing them with iPSC cortical neurons. Co-culture microglia express microglia-specific markers and neurodegenerative disease genes, upregulate homeostatic pathways, develop dynamic ramifications, are phagocytic, and produce a cytokine profile that is co-culture specific.

## Introduction

Microglia are brain-resident macrophages, with important homeostatic functions that provide a supportive environment to neurons. This includes pruning incompetent synapses during development, and clearance of dead cells, misfolded proteins, and other cellular debris (Ransohoff, 2016). However, they can become activated by inflammatory stimuli, producing a battery of cytokines, including the potentially damaging tumor necrosis factor α (TNFα). If not satisfactorily resolved, this response can lead to a chronically damaging cycle of activation and neuronal destruction. Numerous genes associated with Alzheimer's disease (AD), Parkinson's disease (PD), motor neuron disease/amyotrophic lateral sclerosis (MND/ALS), and frontotemporal dementia (FTD) are expressed in microglia, including *TREM2*, *CD33*, *LRRK2*, and *C9orf72* (O'Rourke et al., 2016, Russo et al., 2014, Villegas-Llerena et al., 2016), prompting a growing interest in microglia biology and their relevance to neurodegenerative disease.

Study of microglia has been largely restricted to non-human models (mostly mouse), since availability of fresh primary human microglia is very limited and they cannot be propagated. Moreover, microglia rapidly lose their unique identity when removed from the brain environment and cultured in monoculture in vitro (Butovsky et al., 2014). Transformed microglial-like cell lines are by definition highly proliferative and therefore not a good model for understanding a predominantly non-proliferating, differentiated cell type. There is therefore a need for practical, authentic human microglial cellular models. However, only recently has the ontogeny of microglia been established to inform appropriate modeling.

In mice, two waves of embryonic macrophages are produced in the yolk sac blood islands at embryonic day 7.5 (E7.5) and E8.25, and the first wave migrate into the developing brain and differentiate to microglia (Ginhoux et al., 2010, Gomez Perdiguero et al., 2015, Hoeffel et al., 2015, Palis et al., 1999). These yolk sac-derived macrophages are *Myb* independent but dependent on *PU.1* and *Irf8* (Kierdorf et al., 2013, Schulz et al., 2012). Hematopoietic stem cells (HSCs), in contrast, derive from the aorto-gonado-mesonephros region at day E10.5, populate the fetal liver and bone marrow, and give rise to adult blood cells from HSCs in bone marrow niches, which are dependent on *Myb* for their renewal. *Myb* independence, therefore, distinguishes yolk sac-derived macrophages from adult, definitive, blood monocyte-derived macrophages. Microglia in the developing brain proliferate locally at a low rate and are not normally replaced by other monocytes and macrophages from outside the brain, in contrast to most other tissue-resident macrophages (which also initially originate from yolk sac-derived macrophages, but are partially or fully replaced by fetal liver- or blood monocyte-derived macrophages [Bain et al., 2014, Calderon et al., 2015, Epelman et al., 2014, Guilliams et al., 2014, Hoeffel and Ginhoux, 2015, Tamoutounour et al., 2013]). In the brain, interleukin-34 (IL-34) is an alternative CSF1R ligand supporting microglia survival and differentiation (Greter et al., 2012), and microglia adopt an increasingly ramified morphology and continued maturation far beyond birth.

**Figure 1:**
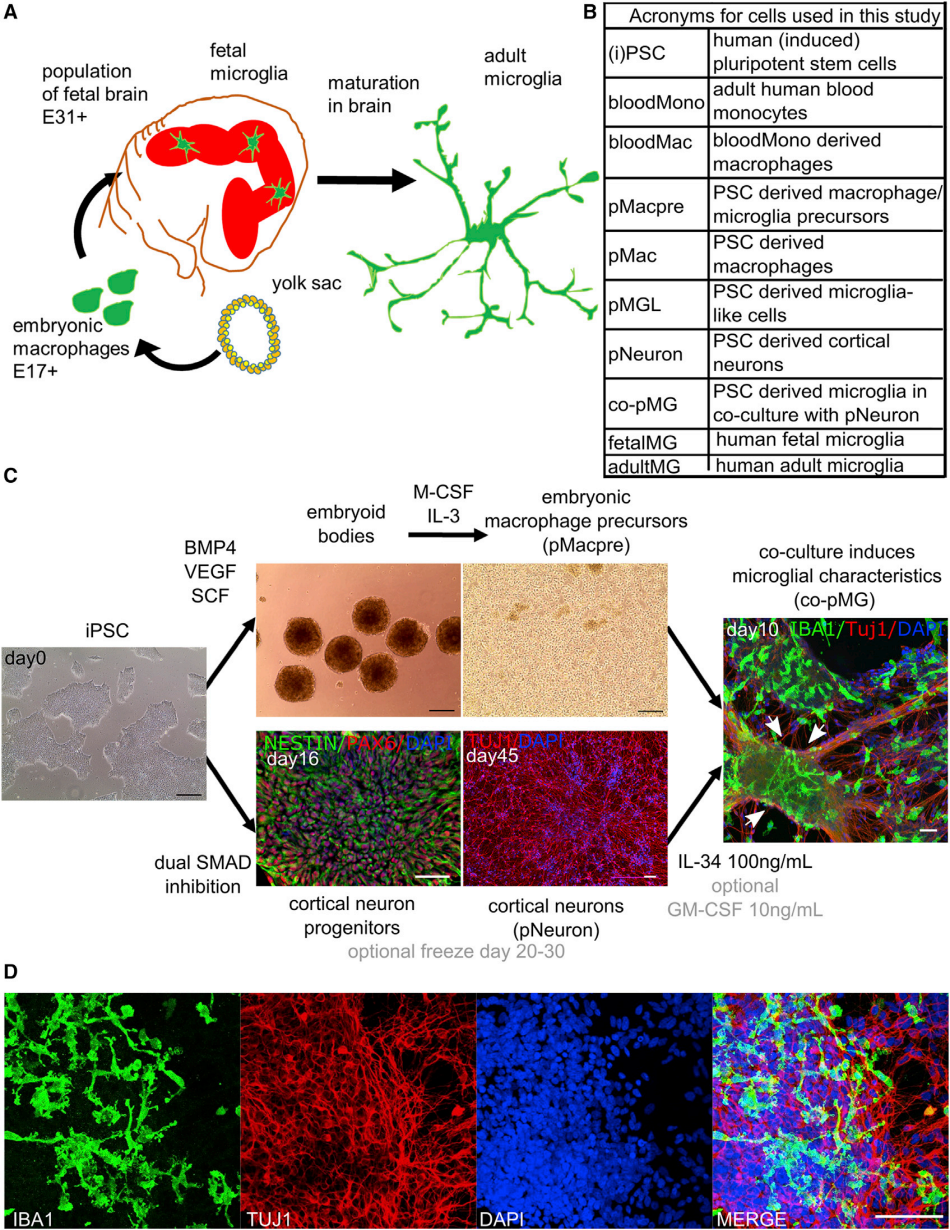
iPSC-Derived Microglia-Neuron Co-culture Recapitulates Microglial Development in the Embryo (A) Human microglia originate from the yolk sac as primitive macrophages, migrating into the fetal brain before the formation of the blood-brain barrier, and completing their maturation in the brain environment. (B) Acronyms used for the cell types in this study. (C) Co-culture of iPSC embryonic macrophages and iPSC-cortical neurons with IL-34 (and, optionally, low level GM-CSF) recapitulates development of microglia in the brain. White arrows: highly ramified cells most evident in dense neuron clusters. (D) Ramified microglia after 2 weeks of co-culture. Black scale bars, 200 μm; white scale bars, 50 μm. See also Figures S1 and S2; Movie S1.

In humans there are few opportunities to investigate the ontogeny of microglia, but it is assumed that the processes are analogous to those in mice. Yolk sac-derived macrophages appear at E17 (Tavian and Peault, 2005), enter the brain from E31 onward (Rezaie et al., 2005, Monier et al., 2007), and mature together with neurons to fully functional ramified microglia (Figure 1A). Human cortical neurons show spontaneous electrical activity after microglia invasion, from gestation week 20 onwards (Moore et al., 2011).

We aimed to recapitulate the in vivo developmental pathway of microglia in vitro, using human induced pluripotent stem cells (iPSCs). These have the advantages of limitless self-renewal and normal karyotype, and can be directed to terminally differentiated cell types. They can be derived from patients (retaining the patient's genetic background) and are amenable to gene editing, enabling sophisticated interrogation of genes of interest. To recapitulate the development of yolk sac-derived macrophages, we use our previously established, straightforward, highly efficient, serum- and feeder-free protocol for deriving PSC macrophages (Karlsson et al., 2008, van Wilgenburg et al., 2013). We have recently directly demonstrated that these derive from *MYB*-independent, *RUNX1-* and *PU.1*-dependent precursors, characteristic of yolk sac-derived macrophages (Buchrieser et al., 2017, Vanhee et al., 2015). Here, we co-culture them with iPSC cortical neurons (Shi et al., 2012), in medium optimized for survival and functionality of both neurons and microglia. The resulting co-cultures are stable for many weeks, express relevant microglia markers (including key disease-related genes), upregulate pathways relating to homeostatic functions, and downregulate pathogen-response pathways. They are phagocytic, display highly dynamic ramifications, respond to activation by clustering and adoption of ameboid morphology, and produce cytokine profiles that are specific to co-culture versus monoculture.

## Results

### iPSC-Derived Embryonic Macrophages Co-cultured with iPSC-Derived Neurons Recapitulate Microglial Development in the Embryo

To recapitulate the development of microglia in the embryo (Figure 1A), we used our previously established protocol (van Wilgenburg et al., 2013) to generate embryonic-like, *MYB*-independent macrophage precursors from iPSCs from four different donors (Table S1). Defined-size embryoid bodies (EBs) are formed using Aggrewells (STEMCELL Technologies), cultured with bone morphogenetic protein 4 (BMP4; to induce mesoderm), vascular endothelial growth factor (VEGF; endothelial precursors), and stem cell factor (SCF; hematopoietic precursors), then plated into large-format flasks with IL-3 and macrophage colony-stimulating factor (M-CSF) to promote myeloid differentiation. Most EBs adhere, put out surrounding adherent stromal cells, and develop cystic, yolk-sac-like structures. After 3–4 weeks, embryonic-like macrophage precursors emerge into the supernatant as a uniform population of large, round cells with obvious filopodia and ruffles. Originally termed “monocytes” (Karlsson et al., 2008, van Wilgenburg et al., 2013), we now understand their ontogeny to be *MYB-*independent primitive myeloid cells (Buchrieser et al., 2017), so they are more accurately termed macrophage precursors (pMacpre, Figure 1B). These can simply be harvested by collecting the supernatant without disrupting the EBs and replenishing flasks with fresh medium for many subsequent weekly harvests. The cumulative yield of pMacpre for the lines used in this study was 10- to 43-fold higher than the number of input iPSCs, consistent with yields previously reported for this protocol (van Wilgenburg et al., 2013), similar to a recently published hiPS-microglia protocol (typically 40-fold) (Abud et al., 2017), and 10-fold higher than two other recently published hiPS-microglia protocols (0.5- to 4-fold and 0.8- to 3-fold yields relative to input iPSCs, respectively) (Muffat et al., 2016, Pandya et al., 2017).

To mimic the subsequent seeding of embryonic macrophages into the developing brain, we co-cultured harvested pMacpre with iPSC-derived cortical neurons (Shi et al., 2012) (Figure 1C). We designed the co-culture medium to maintain microglia survival, which is dependent on signaling through the tyrosine kinase receptor CSF1, so we included the CSF1R ligand IL-34 (the alternative CSF1 ligand produced in the brain, M-CSF being the main ligand in the periphery). We also sought compatibility with iPSC cortical neurons in culture, but aimed to reduce the presence of components in neuronal media that might compromise microglia function, so the neuronal supplement B27 was not included as it contains corticosterone, superoxide dismutase (SOD), and catalase. Finally, in pilot experiments, we tested the ability of different media and growth factors to induce ramified microglia-like morphology in our macrophages, following previous evidence that astrocyte-derived granulocyte M-CSF (GM-CSF), M-CSF, and transforming growth factor β (TGFβ) can each induce ramified morphology in microglia (Schilling et al., 2001), and that IL-34 and GM-CSF can ramify blood monocytes (Etemad et al., 2012, Ohgidani et al., 2014). Advanced DMEM/F12 with N2 and 100 ng/mL IL-34 satisfied the requirement for CSF1R engagement and neuronal compatibility, while IL-34 with (optional) low-dose GM-CSF (10 ng/mL) induced the most ramified morphology (Figures S1A–S1D), so this microglia medium was used for all subsequent experiments (Table S2). In microglia medium and in co-culture with iPSC cortical neurons (pNeurons), pMacpre adopted ramified microglial morphology with secondary branching within 2 weeks (Figures 1C and 1D). They are referred to hereafter as co-culture PSC microglia, or co-pMG. PSC macrophages are termed pMac, unless cultivated in microglia medium (as monocultures), when they are termed PSC microglia-like cells, pMGL.

### Co-culture Is Compatible with iPSC Cortical Neuronal Maturation and Function

Co-culture could be extended for at least 42 days, during which time neurons maintained spontaneous electrical activity at least as well as neurons cultured alone (Figure S1E), and calcium flux was observable upon addition of potassium ions (Movie S1). Pre- and postsynaptic markers (Synaptophysin and PSD95) were observable in neuronal monocultures and in co-cultures (Figures S1F and S1G). Neuronal progenitors present in the cultures continued to proliferate, leading to an increase in the density of the cultures, whereas proliferation, as assessed by Ki-67 staining, was very low in co-pMG (similar to pMGL and pMac, Figures S1H–S1K). For this reason, most assays were conducted after 2 weeks of co-culture. Nonetheless, co-pMG persisted within the extended-duration cultures, maintaining expected density to at least day 39 (Figure S2). Co-pMG were dependent on CSF1R ligand delivery in the culture medium for persistence in co-culture, as withdrawal of IL-34 led to depletion of co-pMG (data not shown). Finally, co-culture neurons expressed both deep-layer (TBR1) and upper-layer (SatB2) cortical identity markers (Figure S2). Together, these observations indicate that co-culture conditions were compatible for co-pMG and had no detrimental effect on the maturity and functionality of the neurons.

### Transcriptome Analysis Demonstrates a Microglial Signature in iPSC Co-culture Microglia

To assess to what extent the co-cultured cells resembled microglia, we isolated co-pMG from the neuronal culture using CD11b magnetic beads and compared their transcriptome with human fetal microglia (fetalMG), pMGL, pMac, pMacpre, and fresh adult blood-derived monocytes (bloodMono).

**Figure 2:**
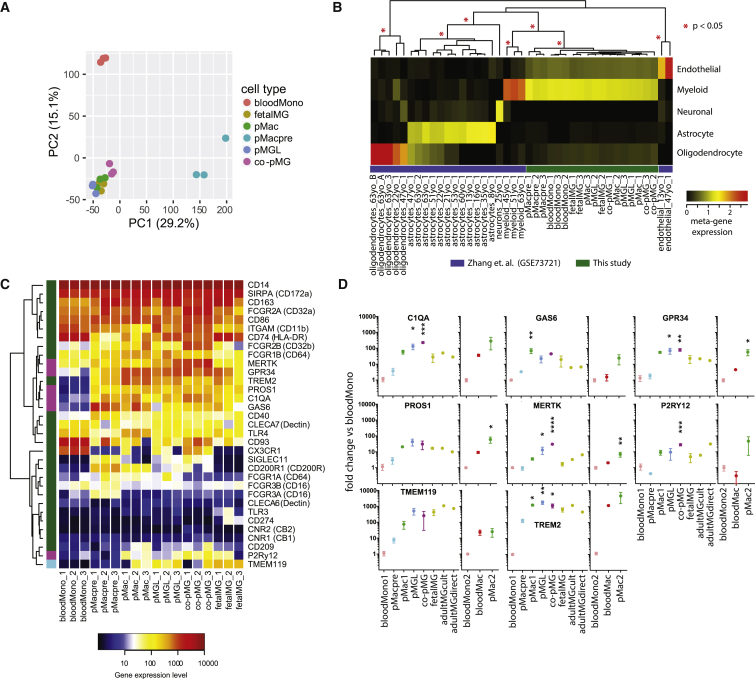
iPSC Co-culture Microglia Express Consensus Microglia Markers Illumina HT12v4 transcriptome analysis of blood monocytes (bloodMono), iPSC-derived macrophage precursors (pMacpre), iPSC-derived macrophages (pMac), iPSC macrophages in microglia medium (pMGL), iPSC co-culture microglia isolated from co-culture (co-pMG), and freshly isolated human primary fetal microglia (fetalMG) (three genetic backgrounds each). (A) Principal component (PC) analysis of samples based on protein coding gene expression. Numbers in parentheses indicate the percentage variance. (B) Cell type analysis. Samples are hierarchically clustered by their expression of metagenes defined by non-negative matrix factorization from a previously published expression dataset for human cortex myeloid cells, cortical oligodendrocytes, astrocytes, neurons, and endothelial cells (Zhang et al., 2016). Red asterisks indicate significant clusters (pvclust, approximately unbiased). (C) Expression of consensus human microglia/monocyte markers. Genes found by Melief et al. (2012) to be highest in microglia are highlighted in green on left-hand bar; six genes identified as being differentially expressed in microglia versus blood monocytes by Butovsky et al. (2014) are magenta, and TMEM119 (Bennett et al., 2016), is azure. Rows are hierarchically clustered. (D) Expression of key microglia markers by qRT-PCR. Fold change was calculated using the ΔΔCT method, with 18S RNA as an endogenous control and normalization to bloodMono. Three genetic backgrounds for all conditions, as per transcriptome samples, with additional comparison with cultured adult human microglia (n = 1, with technical PCR triplicates) and with directly isolated/processed adult human microglia (n = 1, technical PCR triplicates). A second set of bloodMono were also differentiated to macrophages and assessed for these markers, alongside a second batch of pMac (three genetic backgrounds each). Mean ± SEM, one-way ANOVA, Dunnett's multiple comparisons test versus bloodMono. ^∗^p < 0.05, ^∗∗^p < 0.01, ^∗∗∗^p < 0.001, ^∗∗∗∗^p < 0.0001. See Figures S3 and S4 for further transcriptomic analyses.

Based on their first two principal components, the samples separated into three distinct groups comprised of (1) bloodMono, (2) pMacpre, and (3) pMac, pMGL, co-pMG, and fetalMG (Figure 2A). The bloodMono and pMacpre samples showed an orthogonal separation from the macrophage and microglia samples that is in line with the different developmental origins of these cells. To investigate this possibility, we identified the set of genes significantly differentially expressed between these two populations (Figure S3 and Table S3). *FLT3*, a marker of definitive hematopoiesis, showed higher expression in the bloodMono samples (5.5-fold, adjusted p < 2.4 × 10^−8^), along with several *HLA* genes, in agreement with blood monocytes exposure to priming cytokines in the blood and their role in antigen presentation to T cells. Meanwhile *MAF*, a known marker of primitive hematopoiesis, showed higher expression in pMacpre (21.7-fold, adjusted p < 7.8 × 10^−7^). *APOE*, variants of which are major risk factors for AD, was among the most strongly differentially expressed genes, being very low in bloodMono and high in all other populations (Figure S3A).

Comparison with a previously published expression dataset for cells derived from human brain tissue (Zhang et al., 2016) showed that all the cell types in the current study cluster with human cortex myeloid cells and not with cortical oligodendrocytes, astrocytes, neurons, or endothelial cells (Figure 2B).

As a set, genes previously identified to be associated specifically with microglia but not with blood monocytes (Bennett et al., 2016, Butovsky et al., 2014, Melief et al., 2012) (Figure 2C) showed similar expression in co-pMG and fetalMG. Most notably, the six key microglia-specific genes identified by Butovsky et al. (2014), MERTK, GPR34, PROS1, C1QA, GAS6, and P2RY12, were all strongly expressed in co-pMG, whereas bloodMono poorly expressed most of these markers (highlighted in magenta, Figure 2C) These expression profiles were confirmed by qRT-PCR (Figure 2D), which also showed that co-pMG had comparable levels of expression of these microglial genes with cultured adult human microglia and with directly processed adult human microglia, and that while blood monocyte-derived macrophages also upregulated several of these genes, they mostly did not reach the same expression level. Notably, pMacpre, pMac, and pMGL also expressed high levels of most of these genes, along with many of those identified by Melief et al. (2012).

Next, we investigated the difference between the iPSC-derived macrophage and microglia populations. A targeted principal components analysis of these samples revealed a weak neural cell signature in the co-cultured pMG isolated with CD11b beads, but otherwise demonstrated the close similarity of these cells to fetal microglia (Figures S4A and S4B). We then sought to better understand the transcriptional differences between the differentiated PSC-derived samples pMac, pMGL, and co-pMG. We focused on genes with high and significantly variable expression using k-means clustering to identify five distinct signatures of gene expression (Figure S4C). Gene ontology analysis identified biological processes with significant enrichment in these gene sets, demonstrating that co-pMG downregulate genes in pathways associated with type I interferon responses (involved in antiviral responses), Toll-like receptor 1 (TLR1) and TLR2 signaling (bacterial and yeast recognition), and antigen presentation, relative to the monoculture populations. This implies that co-culture with neurons downregulates responses to external pathogens. Meanwhile, genes upregulated in co-pMG were enriched for biological processes including differentiation, chemotaxis/migration, regulation of cell-cell adhesion, and metal ion response (Hancock et al., 2014), all of which would be important for microglia to carry out their homeostatic surveillance and clearing functions.

Taken together, these results show the transcriptomic similarity of co-pMG with primary microglia, with expression of key microglial markers and genes in relevant homeostatic pathways, and downregulation of antimicrobial pathways in co-pMG. However, these results also highlight a previously unappreciated detail, which is that genes that have been previously identified as being specific to microglia versus blood monocytes are not necessarily exclusive to microglia, but a subset of them are more likely correlates of primitive macrophages, since they are also highly expressed in pMac.

### Co-culture Microglia Express Genes Associated with Major Neurodegenerative Disease

**Figure 3:**
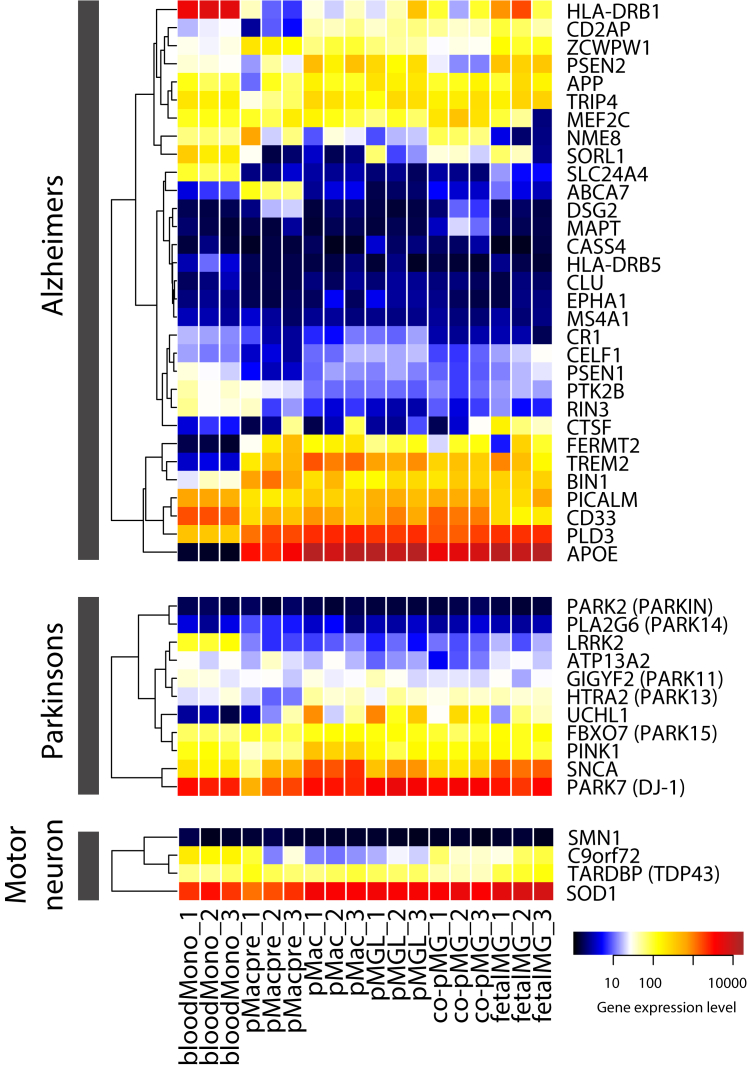
Expression of Genes Associated with Major Neurodegenerative Disorders Samples and expression dataset as per Figure 2.

Because numerous genes associated with AD, PD, MND/ALS, and FTD (through acquisition of mutations or SNP variants) have been found to be expressed in microglia, we examined the expression of these genes in our transcriptome dataset (Figure 3). *FERMT2*, *TREM2*, *APOE*, and *UCHL1* were expressed in fetalMG and co-pMG but not in bloodMono (although TREM2 was upregulated in bloodMono-derived macrophages, Figure 2D). Other key AD-related genes expressed in fetalMG and co-pMG (and bloodMono) included *APP*, *PICALM*, and *CD33*; PD-related genes included *PARK15*, *PINK1*, *SNCA*, and *DJ-1*; and MND-related genes included *C9orf72*, *TDP43*, and *SOD1*. Note that almost all of these genes were also expressed in pMacpre, pMac, and pMGL. Together, this shows that our co-culture system is a relevant model to study the effects of numerous genes associated with neurodegenerative disease, and that monoculture pMac or pMGL can be useful for answering specific disease gene-related questions where co-culture is impractical.

### Co-culture Microglia Express Macrophage-/Microglia-Relevant Proteins

**Figure 4:**
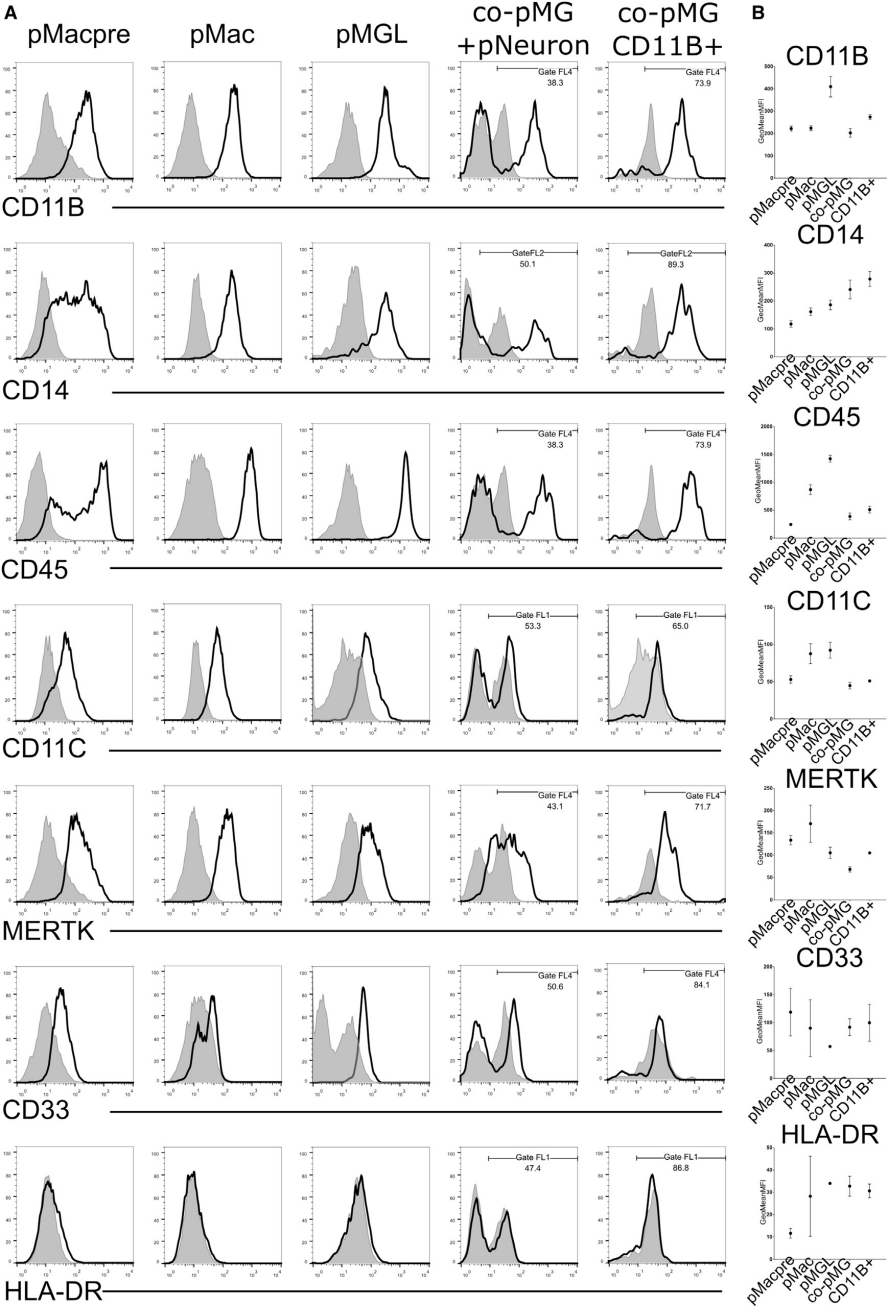
iPSC Co-culture Microglia Express Relevant Protein Markers (A) Flow cytometry of cells differentiated from one line (SFC856-03-04, black line is surface marker, filled gray area is isotype control). pMacPre were differentiated for 14 days to pMac, pMGL, or co-cultured with neurons to obtain co-pMG, which were either stained in single-cell suspension of the co-culture or isolated with CD11b beads before staining. (B) Expression of surface markers (three genetic backgrounds, lines SBAD3-01, SFC840-03-03, SFC856-03-04) in pMacpre, pMac, pMGL, and co-cultures at 2, 7, 10, and 14 days. To remove obviously non-myeloid cells from MFI analysis, we set a gate to FL1, FL2, and FL4 for all cytometry data. Error bars denote SEM. See Figure S7 for FSC/SSC gating and additional cytometry data.

We next sought evidence for the functional protein products of key microglia genes. Flow cytometry with directly conjugated antibodies showed CD11b (integrin alpha M, a marker for mature myeloid cells and a subunit of the complement receptor, CR3, also known as Mac-1), CD14 (a component of the receptor for bacterial lipopolysaccharide [LPS]), and CD45 (a pan-leukocyte marker and tyrosine phosphatase which dephosphorylates several receptor tyrosine kinases), were expressed on all PSC-derived macrophage and microglia (Figures 4A and 4B). CD11c (integrin alpha X, part of the inactivated-C3b receptor 4, CR4), was well expressed in monoculture cells, but weakly expressed in co-pMG. HLA-DR (a major histocompatibility complex [MHC] class II antigen) was undetectable in any PSC-derived myeloid lineages, reflecting the unprimed culture conditions. The microglia-associated protein MERTK was expressed highly on all PSC-derived macrophage and microglia conditions, while the AD-associated protein CD33 was detected albeit at low levels. An unconjugated polyclonal antibody to TMEM119 gave modest staining of pMGL but background staining was evident in co-culture cells, and an unconjugated antibody to the purinergic receptor P2YR12 showed strong staining of pMGL although background staining was similarly evident in co-culture (Figure S5). These markers did not increase in a consistent way during the time course of the co-cultures (Figures S5C–S5E). IBA1 (a cytoplasmic calcium-binding protein associated with myeloid cells, particularly microglia) was readily detectable in co-pMG by immunocytochemistry (Figures 1B and 1C). These results show that co-pMG and their primitive precursors express expected microglia and myeloid-associated proteins, although PSC macrophages and microglia have not been exposed to the cytokine milieu of the body and thus have low basal levels of proteins such as MHC antigens.

### Co-culture Promotes Microglial Ramification and Motility

**Figure 5:**
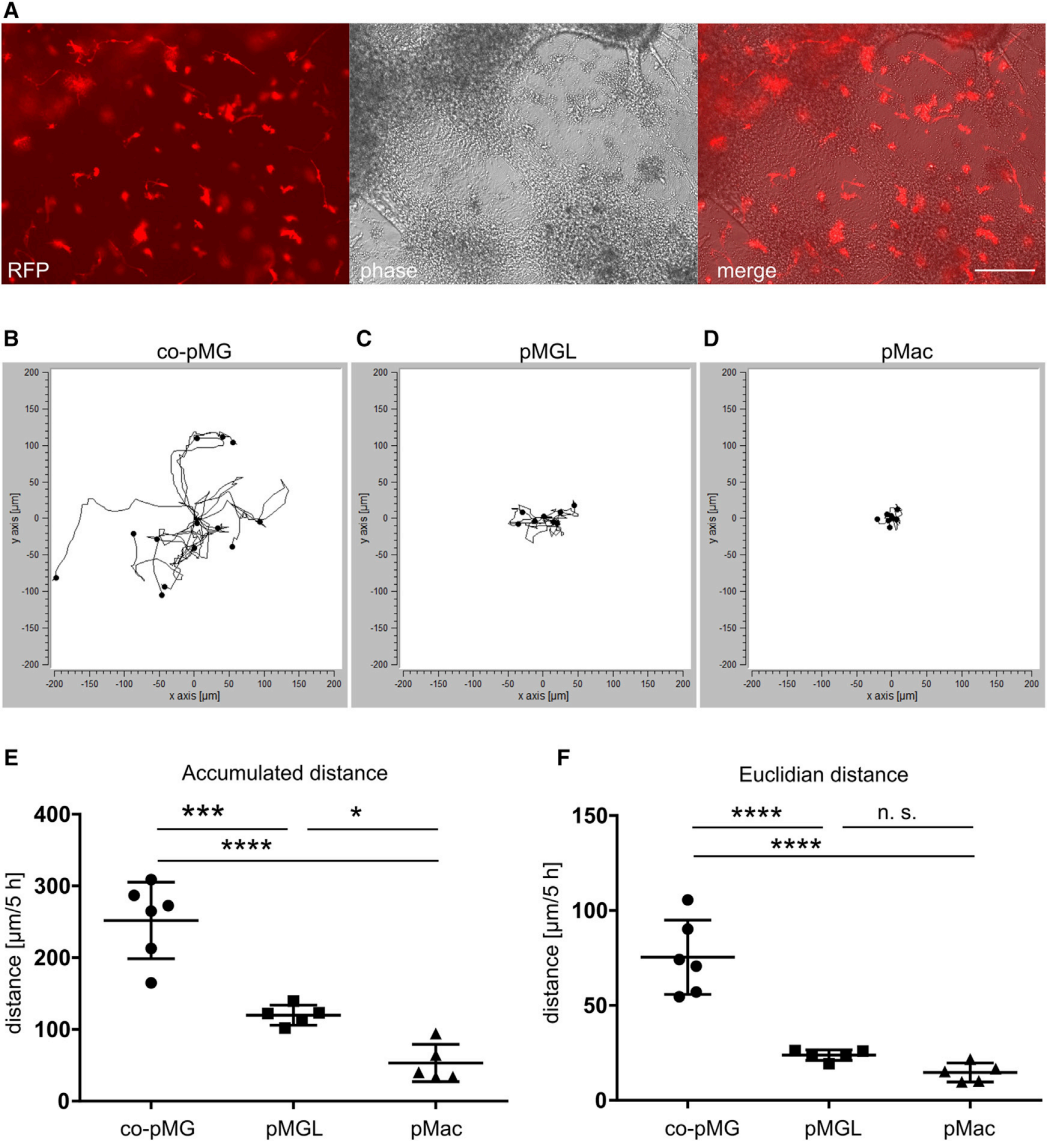
Co-culture with Neurons Promotes iPSC-Microglial Motility Macrophages and co-culture microglia were imaged every 5 min for 5 hr (two videos each of three cultures i.e., six replicates per condition). (A) co-pMG expressing RFP to enable identification in co-culture. Scale bar, 200 μm. (B–D) Tracks of co-culture microglia (co-pMG; B), compared with cells on tissue-culture plastic: pMGL (C) and pMac (D). |(E) Accumulated distance. (F) Euclidian distance (distance in a straight line, start to end). Error bars represent SD. Statistical analysis by Tukey's multiple comparisons test. ^∗^p < 0.05, ^∗∗∗^p < 0.001, ^∗∗∗∗^p < 0.0001; n.s., not significant. See also Movies S2, S3, andS4.

To identify and image microglia live in co-culture, we used co-pMG differentiated from an iPSC line containing multiple copies of an integrated lentivector containing the RFP gene under the control of the constitutively active EF-1α promoter. Co-pMG roughly “tile” within the neuronal culture, making direct contacts with the neurons (Figure 5A). Live imaging revealed a dynamically remodeled ramified morphology, every 5-min and even 12-s frame revealing changes to primary and secondary branching (Movies S2 and S3). Co-pMG moved constantly, most of them roughly maintaining their territories. Quantification of movement of co-pMG, pMGL, and pMac over 5 hr showed co-pMG moved a significantly greater accumulated distance (251 ± 21 μm; mean ± SEM; n = 6) than pMGL (119 ± 6 μm; n = 5) and pMac (53 ± 11 μm; n = 5), which hardly moved at all (Figures 5B–5F). Together, these results show that co-pMG display the morphology and dynamic behavior expected of microglia, continually sensing and responding to their neuronal environment. These features were a direct result of physical contact with neurons, as cells monocultured on tissue-culture plastic did not display such dynamic microglial characteristics.

### Co-culture Microglia Are Phagocytically Competent

We have previously demonstrated that pMac are competent at phagocytosing particles, progressive acidification of the maturing phagosome being detectable using particles coupled to pH-sensitive fluorophores (Kapellos et al., 2016). pH-sensitive fluorescent zymosan particles added to co-cultures became visible inside co-pMG, within 1 hr, comparable with pMac (Movie S4), indicating the competent development of mature phagosomes in co-pMG.

### iPSC Microglia Display Co-culture-Specific Inflammatory Responses

**Figure 6:**
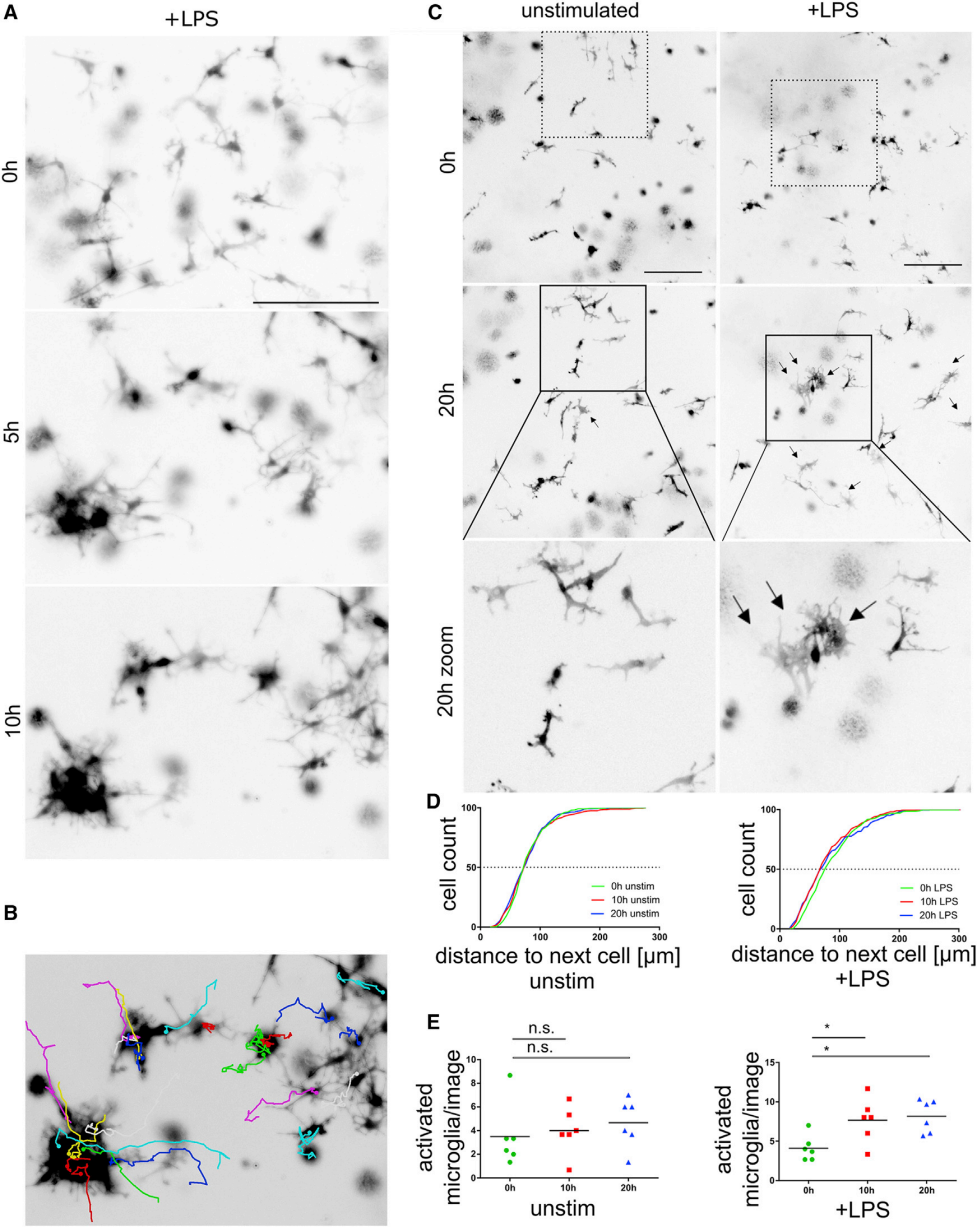
LPS Induces Inflammatory Morphology and Clustering in Co-culture Microglia (A–D) Microglial morphology displayed as inverted LUT black and white images of RFP-iPSC microglia in co-culture. (A) Images (co-culture day 12) every 5 min for 10 hr. Representative images of LPS-stimulated co-pMG are shown at 0, 5, and 10 hr. (B) co-pMG clustering on LPS stimulation shown by cell tracking. (C–E) Quantitative analysis (two videos each of three cultures, i.e., six replicates per condition). Images were taken on co-culture on day 14 every 5 min for 20 hr. (C) Representative images of 0-hr and 20-hr time points; Bottom panel: area in the black square magnified to show microglial morphology: unstimulated co-pMG show no obvious changes in morphology during imaging period, but on LPS stimulation some microglia cluster, adopting more ameboid morphology with shorter processes and higher area-to-perimeter ratio, representative of a pro-inflammatory phenotype (black arrows). (D) Distance between microglia clustering upon stimulation is evidenced by a leftward shift of the plot after 10 hr and 20 hr. (E) Micrographs were scored blind by three independent assessors for number of microglia with activated morphology. Statistical analysis by Dunnett's multiple comparison test. n.s., not significant; ^∗^p < 0.05. Scale bars, 200 μm. See also Movie S5.

To explore the ability of co-pMG to respond physically to inflammatory signals, we stimulated co-cultures with LPS and imaged them over the next 20 hr. Unstimulated co-pMG retained roughly territorial surveillance behavior over the whole imaging period (Movie S5), suggesting no imaging-induced activation. In contrast, within 5 hr LPS-stimulated co-pMG migrated to form clusters, and some microglia had reduced ramifications and increased area-to-perimeter ratio, indicative of transition to activated, ameboid microglia (Figures 6A–6C and Movie S5). Clustering was measured as distance to nearest neighbor, showing a leftward shift (i.e., smaller distance) in curves for LPS-treated cultures, indicative of the clustering clearly observable by eye (Figure 6D). Blinded morphology scoring showed a significant increase in the proportion of cells with activated morphology in LPS-treated cultures in six analyzed videos (time point mean ± SEM: 0 hr, 4.1 ± 0.6; 10 hr, 7.6 ± 1.1; 20 hr, 8.1 ± 0.8; Figure 6E).

**Figure 7:**
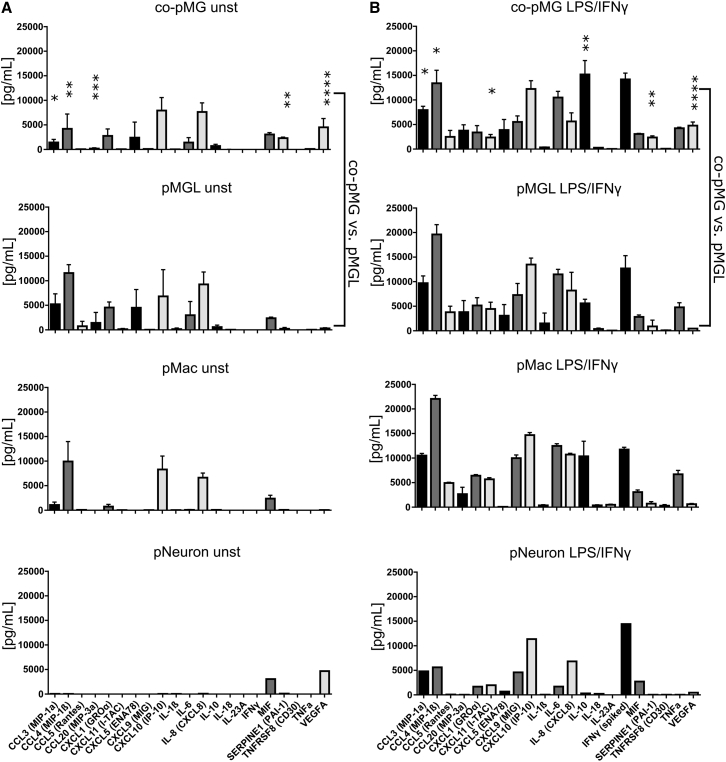
Cytokine Profiles of Co-culture versus Monocultures Eighteen-hour supernatants from cells stimulated with or without LPS/IFNγ were assayed with a Luminex multiplex assay. DMEM/F12/N2-based microglia medium was used for monoculture (pMGL), co-culture (co-pMG), and neuron-only culture (pNeuron); standard XVIVO^15^-based macrophage medium was used for pMac. Medium alone contained negligible levels of all cytokines tested. Supernatants from lines: SBAD3-01 neurons ± SFC180-01-01, SFC840-03-03, and SFC856-03-04 macrophages/microglia. (A) Unstimulated cells. (B) LPS/IFNγ-stimulated cells. Mean ± SD, three genetic backgrounds. Asterisks indicate significant difference between co-pMG and pMGL by two-tailed paired t test. ^∗^p < 0.05, ^∗∗^p < 0.01, ^∗∗∗^p < 0.001, ^∗∗∗∗^p < 0.0001. See Table S2 for further factors tested with pMac and co-pMG, and Figure S6 for effect of additional media and growth factor combinations on cytokine secretion.

To examine the cytokine responses of co-pMG, we compared co-pMG and pMac using Proteome Profiler for 102 cytokines with and without maximal activation (LPS/interferon-γ [IFNγ], Table S2). Differentially expressed cytokines were then selected for a more detailed investigation using Luminex multiplex array, with supernatants from pMac, pMGL, co-pMG, and pNeuron, with or without LPS/IFNγ stimulation (Figure 7). There was broad correspondence across Proteome Profiler and Luminex platforms. Unstimulated neurons secreted only macrophage migration inhibitory factor (MIF) and VEGF-A, and when stimulated secreted IL-6, IL-8, and a subset of chemokines. pMac secreted very few cytokines constitutively (macrophage inflammatory protein 1α [MIP1α] and MIPβ, CXCL1 and CXCL10, IL-8, and MIF), but secreted the entire panel of 22 cytokines upon stimulation, in concordance with our previous publication (which also includes comparison with blood monocyte-derived macrophages [Jiang et al., 2012]). pMGL had a higher baseline number of cytokines secreted, and upregulated most cytokines upon stimulation (except IL-23A). GM-CSF did not account for this difference, as its absence did not significantly change the cytokine profile of pMGL (Figure S6).

Co-pMG displayed an overall dampened secretion of chemokines and cytokines versus pMGL, both constitutive and induced. Interestingly, SerpinE1 (a serine protease inhibitor that inhibits fibrinolysis) and VEGF-A (stimulates angiogenesis) were significantly higher in activated co-culture versus monoculture, suggesting that tissue remodeling factors are specifically induced in co-culture. Meanwhile, IL-10, which is anti-inflammatory, was significantly increased in activated co-culture versus monocultures. Together, these results indicate that co-culture induces specific anti-inflammatory and pro-remodeling responses not seen in corresponding monocultures.

## Discussion

We have established a highly efficient in vitro human iPSC-derived microglia-neuron co-culture model, which recapitulates the ontogenetic development of microglia in vivo. Co-pMG can be maintained in co-culture, retaining neuronal maturity and functionality for many weeks. They express key human microglia-specific markers and neurodegenerative disease-relevant genes, upregulate homeostatic pathways, downregulate pathogen-response pathways, and exhibit a transcriptional profile similar to that of fetal microglia. Co-pMG are phagocytic, adopt a highly dynamic ramified microglia-like morphology, upon activation display an activated morphology, and release a battery of microglia-relevant cytokines, with co-culture promoting a more anti-inflammatory and pro-remodeling cytokine response than corresponding monocultures. This shows the relevance of using co-culture to examine direct and paracrine microglia-neuronal interactions.

Since primary human microglia can only be obtained from fresh brain material, previous efforts have been made by others to develop methods for deriving microglia in vitro. Etemad and colleagues used human blood monocytes as the starting point (Etemad et al., 2012, Ohgidani et al., 2014), but it is now known that this pathway poorly recapitulates microglia ontogenesis and that blood is not a limitless source of cells, unlike PSCs. Beutner et al. (2013) described a method for deriving microglia from mouse embryonic stem cells, and, using the same methodology, Almeida et al. (2012) derived microglia from human iPSCs. However, this method directs the EBs through a neuronal differentiation pathway and thus does not replicate yolk sac myelopoiesis. Moreover, these cells do not express a convincing microglia signature (Butovsky et al., 2014, Melief et al., 2012). Schwartz et al. (2015) recently successfully seeded iPSC macrophages into 3D iPSC-neuronal structures for toxicity testing, but did not characterize the resulting cells extensively.

In 2008, we derived human PSC macrophages using a very simple methodology without OP9 feeders (Karlsson et al., 2008), which we subsequently adapted to a fully defined, robust serum-free protocol (van Wilgenburg et al., 2013), now widely used by others (Aflaki et al., 2014, Alasoo et al., 2015, Gupta et al., 2016). Our own work (Buchrieser et al., 2017) and that of others (Hoeffel et al., 2015) indicates that this protocol produces *MYB*-independent myeloid cells, recapitulating an embryonic ontogeny, and lineage-tracking studies in mouse have demonstrated that microglia derive from primitive, yolk sac macrophages that migrate into the developing brain (Ginhoux et al., 2010, Ginhoux et al., 2013). Together, this provides a rationale for deriving PSC microglia, using PSC macrophages as a starting point and then skewing them toward a microglial phenotype. During the drafting of this manuscript, three protocols along these general principles have been published (Abud et al., 2017, Muffat et al., 2016, Pandya et al., 2017). Where our analyses overlap, there is broad consensus. However, only our protocol has direct evidence by gene knockout for producing *MYB*-independent primitive macrophages (Buchrieser et al., 2017). It is robust and efficient: time frame 1 month, versus 2 months for Muffat et al. (2016); yield 10–43 per starting iPSC for our protocol, versus 0.5–4 for Muffat et al. (2016) and Pandya et al. (2017); manipulation consisting of once-weekly feeding of differentiation cultures in flasks, simple supernatant harvest of pure precursors, and multiple harvests possible, versus sequential trituration/replating steps for Muffat et al. (2016), fluorescence-activated cell (FAC) sorting/plating on astrocytes/second FAC sorting for Pandya et al. (2017), and low oxygen concentrations/several replatings/five different cytokine cocktails/FAC sorting progenitors for Abud et al. (2017). Our microglia medium avoids B27, which contains cortisone, SOD, and catalase (likely to compromise microglia function). Muffat et al. (2016) characterized their iPSC microglia in monoculture, briefly assessing co-culture, and Pandya et al. (2017) characterized their iPSC microglia in monoculture following isolation from astrocytes. We have extensively characterized iPSC microglia in co-culture with iPSC cortical neurons, where cells have highly ramified, dynamic characteristics, and compared directly to the intermediate and parallel monoculture stages of differentiation. This reveals that while key microglia genes are expressed in co-pMG, they are often also expressed in pMacpre, pMac, and pMGL, indicating that such genes may be features of primitive macrophages rather than being microglia specific, and that comparison only with blood monocytes/macrophages does not give a complete interpretation. Finally, we show that co-culture induces a unique cytokine profile which is not the sum of monocultures.

There is a fast-growing interest in microglia, as they are increasingly implicated in neurodegenerative disease, neurodevelopmental disorders, and in neuropathic pain. Our iPSC microglia transcribe key genes involved in AD, PD, and MND. Several other disease-associated genes, including *LRRK2*, would be expected to be upregulated upon microglial stimulation (reviewed in Lee et al., 2017). Many of these genes are likely involved in phagocytosis and processing of misfolded proteins and of dying neurons (common features of these diseases), and in generating inappropriate chronic cytokine responses that exacerbate neuronal damage, creating a destructive cycle. Human iPSC microglia models enable study of these gene products at their correct gene dosage, in an authentic human in vitro system. Some of these functions and disease-relevant genes can be studied in the monoculture conditions detailed here, but others, involving crosstalk between microglia and neurons, such as paired receptor engagement, paracrine signaling, damage responses, synaptic surveillance, and pruning, will be better studied using the co-culture model we have described. The system is also amenable to scaling for the development of drug-screening assays to identify compounds that can improve microglial homeostatic clearance functions and dampen chronically activated microglia.

## Experimental Procedures

### Consent for Use of Human Material

All human material (iPSCs, adult blood, fetal and adult microglia) was obtained with informed consent and with the approval of the relevant institutions (see Supplemental Information for full details).

### Macrophage Differentiation

See Supplemental Experimental Procedures for details of cells and assays used. iPSCs were differentiated to macrophages as previously described (van Wilgenburg et al., 2013). In short, 3 × 10^6^ iPSCs were seeded into an Aggrewell 800 well (STEMCELL Technologies) to form EBs, in mTeSR1 and fed daily with medium plus 50 ng/mL BMP4 (Peprotech), 50 ng/mL VEGF (Peprotech), and 20 ng/mL SCF (Miltenyi Biotec). Four-day EBs were then differentiated in either 6-well plates (15 EBs/well), T75 (75 EBs), or T175 flasks (150 EBs) in X-VIVO15 (Lonza), supplemented with 100 ng/mL M-CSF (Invitrogen), 25 ng/mL IL-3 (R&D), 2 mM Glutamax (Invitrogen), 100 U/mL penicillin and 100 μg/mL streptomycin (Invitrogen), and 0.055 mM β-mercaptoethanol (Invitrogen), with fresh medium added weekly. pMacpre emerging into the supernatant after approximately 1 month were collected weekly and differentiation cultures replenished with fresh medium. Harvested cells were strained (40 μm, Corning) and used: either directly as pMacpre; or plated onto tissue-culture treated plastic or glass coverslips at 100,000 per cm^2^ and differentiated for 7 days or more to pMac in X-VIVO15 with 100 ng/mL M-CSF, 2 mM Glutamax, 100 U/mL penicillin, and 100 μg/mL streptomycin; or co-cultured with iPSC-derived neurons.

### Neuronal Differentiation

iPSCs were differentiated to cortical neuron progenitors (NPCs) (Shi et al., 2012) with the following modifications: feeder-free iPSCs were plated onto Matrigel-coated 6-well plates, with neural induction for 12 days using dual SMAD inhibition; after replating the neuroepithelial sheet at day 12 using dispase, 20 ng/mL fibroblast growth factor 2 (FGF2) was added to neural maintenance medium (NMM) on days 13–17, newly formed rosettes were dispased on days 17–18, and stocks of NPC were frozen at days 25–30.

### Microglia Medium

In pilot experiments, macrophages were differentiated in three different basal media (XVIVO15, RPMI, or Advanced DMEM/F12 + N2 supplement) supplemented with 2 mM Glutamax, 100 U/mL penicillin and 100 μg/mL streptomycin, and 0.055 μM β-mercaptoethanol, with combinations of 100 ng/mL M-CSF (Invitrogen), 100 ng/mL IL-34 (Peprotech or Biolegend), and 10 ng/mL GM-CSF (Invitrogen). ADMEM/F12 + N2 supplement +100 ng/mL IL-34 + 10 ng/mL GM-CSF microglia medium was used for all further experiments (for details see Table S2).

### Microglia-Neuron Co-culture

NPCs were thawed, centrifuged (200 × *g*, 10-fold volume NMM), and plated onto Matrigel in NMM supplemented with 10 μmol/L Y-27632 and 20 ng/mL FGF2. Medium was replaced the next day and every other day thereafter with NMM. After 7 days they were dissociated to single cells with StemPro Accutase (STEMCELL), added to their final Matrigel-coated format (Corning 96-well or 6-well plate, or Ibidi 8-well slide) at 100,000 cells/cm^2^ and cultured for 14 days in NMM. pMacpre were resuspended in microglia medium, added at 100,000 cells/cm^2^ to neurons, and co-cultured for a minimum 14 days before assaying.

### Statistics

GraphPad Prism was used for statistical analysis. One-way ANOVA and Dunnett's multiple comparisons, Tukey's multiple comparisons, or paired two-tailed t test (for single comparisons) were used as indicated. Values are indicated in figures as ^∗^p < 0.05, ^∗∗^p < 0.01, ^∗∗∗^p < 0.001, ^∗∗∗∗^p < 0.0001, and n.s. (not significant). Numbers in parentheses are (mean ± SEM, n).

## Author Contributions

Conceptualization, S.A.C., W.S.J., and W.H.; Methodology, S.E.N., S.C., W.H., S.A.C., and S.N.S.; Investigation, W.H.; Formal Analysis, W.H. and S.N.S.; Writing, W.H. and S.A.C.; Writing – Original Draft, W.H., S.N.S., and S.A.C.; Writing – Review & Editing, S.A.C., R.W.-M., M.Z.C., and W.S.J.; Funding Acquisition, S.A.C., R.W.-M., M.Z.C., W.H., and W.S.J.; Resources, N.D.A., S.E.N., C.S., F.J.N., C.S.M., J.P.A., S.C., S.N.S., and J.B.; Supervision, S.A.C. and W.S.J.

## Accession Numbers

The accession number for the Illumina HT12v4 expression array datasets reported in this paper is GEO: GSE89795.

## Supplemental Information

Document S1. Supplemental Experimental Procedures, Figures S1–S6, and Tables S1 and S2

Table S3. Transcriptomics Data and Differential Expression Results

Movie S1. Calcium Imaging in Neuronal Monoculture Followed by Images of Co-culture; Images Were Taken Every 3 Seconds for 2 Minutes, Related to Figure 1

Movie S2. RFP Co-culture Microglia Unstimulated, Red Channel/Phase Overlay; Images Were Taken Every 5 Minutes for 20 Hours, Related to Figure 5

Movie S3. RFP Co-culture Microglia Unstimulated, Red Channel Red Channel Inverted LUT; Images Were Taken Every 12 Seconds for 1 Hour, Related to Figure 5

Movie S4. RFP Co-Culture Microglia Fed pHrodo Green Zymosan, Followed by RFP pMac Fed pHrodo Green Zymosan; Images Were Taken Every 10 Minutes for 3 Hours, Related to Figure 5

Movie S5. RFP Co-culture Microglia Unstimulated Day 12, Followed by RFP Co-culture Microglia Stimulated with LPS, Red Channel Inverted LUT; Images Were Taken Every 5 Minutes for 17.5 Hours, Related to Figure 6

Document S2. Article plus Supplemental Information

## References

[bib1] Abud E., Ramirez R., Martinez E., Healy L., Nguyen C., Newman S., Yeromin A., Scarfone V., Marsh S., Fimbres C. (2017). iPSC-derived human microglia-like cells to study neurological diseases. Neuron.

[bib2] Aflaki E., Stubblefield B., Maniwang E., Lopez G., Moaven N., Goldin E., Marugan J., Patnaik S., Dutra A., Southall N. (2014). Macrophage models of Gaucher disease for evaluating disease pathogenesis and candidate drugs. Sci. Transl. Med..

[bib3] Alasoo K., Martinez F.O., Hale C., Gordon S., Powrie F., Dougan G., Mukhopadhyay S., Gaffney D.J. (2015). Transcriptional profiling of macrophages derived from monocytes and iPS cells identifies a conserved response to LPS and novel alternative transcription. Sci. Rep..

[bib4] Almeida S., Zhang Z., Coppola G., Mao W., Futai K., Karydas A., Geschwind M.D., Tartaglia M.C., Gao F., Gianni D. (2012). Induced pluripotent stem cell models of progranulin-deficient frontotemporal dementia uncover specific reversible neuronal defects. Cell Rep..

[bib5] Bain C.C., Bravo-Blas A., Scott C.L., Gomez Perdiguero E., Geissmann F., Henri S., Malissen B., Osborne L.C., Artis D., Mowat A.M. (2014). Constant replenishment from circulating monocytes maintains the macrophage pool in the intestine of adult mice. Nat. Immunol..

[bib6] Bennett M., Bennett C., Liddelow S., Ajami B., Zamanian J., Fernhoff N., Mulinyawe S., Bohlen C., Adil A., Tucker A. (2016). New tools for studying microglia in the mouse and human CNS. Proc. Natl. Acad. Sci. USA.

[bib7] Beutner C., Linnartz-Gerlach B., Schmidt S.V., Beyer M., Mallmann M.R., Staratschek-Jox A., Schultze J.L., Neumann H. (2013). Unique transcriptome signature of mouse microglia. Glia.

[bib8] Buchrieser J., James W., Moore M. (2017). Human induced pluripotent stem cell-derived macrophages share ontogeny with MYB-independent tissue-resident macrophages. Stem Cell Rep..

[bib9] Butovsky O., Jedrychowski M.P., Moore C.S., Cialic R., Lanser A.J., Gabriely G., Koeglsperger T., Dake B., Wu P.M., Doykan C.E. (2014). Identification of a unique TGF-beta-dependent molecular and functional signature in microglia. Nat. Neurosci..

[bib10] Calderon B., Carrero J.A., Ferris S.T., Sojka D.K., Moore L., Epelman S., Murphy K.M., Yokoyama W.M., Randolph G.J., Unanue E.R. (2015). The pancreas anatomy conditions the origin and properties of resident macrophages. J. Exp. Med..

[bib11] Epelman S., Lavine K.J., Beaudin A.E., Sojka D.K., Carrero J.A., Calderon B., Brija T., Gautier E.L., Ivanov S., Satpathy A.T. (2014). Embryonic and adult-derived resident cardiac macrophages are maintained through distinct mechanisms at steady state and during inflammation. Immunity.

[bib12] Etemad S., Zamin R.M., Ruitenberg M.J., Filgueira L. (2012). A novel in vitro human microglia model: characterization of human monocyte-derived microglia. J. Neurosci. Methods.

[bib13] Ginhoux F., Greter M., Leboeuf M., Nandi S., See P., Gokhan S., Mehler M., Conway S., Ng L., Stanley R. (2010). Fate mapping analysis reveals that adult microglia derive from primitive macrophages. Science.

[bib14] Ginhoux F., Lim S., Hoeffel G., Low D., Huber T. (2013). Origin and differentiation of microglia. Front. Cell Neurosci..

[bib15] Gomez Perdiguero E., Klapproth K., Schulz C., Busch K., Azzoni E., Crozet L., Garner H., Trouillet C., de Bruijn M.F., Geissmann F. (2015). Tissue-resident macrophages originate from yolk-sac-derived erythro-myeloid progenitors. Nature.

[bib16] Greter M., Lelios I., Pelczar P., Hoeffel G., Price J., Leboeuf M., Kundig T.M., Frei K., Ginhoux F., Merad M. (2012). Stroma-derived interleukin-34 controls the development and maintenance of Langerhans cells and the maintenance of microglia. Immunity.

[bib17] Guilliams M., Ginhoux F., Jakubzick C., Naik S., Onai N., Schraml B., Segura E., Tussiwand R., Yona S. (2014). Dendritic cells, monocytes and macrophages: a unified nomenclature based on ontogeny. Nat. Rev. Immunol..

[bib18] Gupta R., Meissner T., Cowan C., Musunuru K. (2016). Genome-edited human pluripotent stem cell–derived macrophages as a model of reverse cholesterol transport—brief report. Arterioscler. Thromb. Vasc. Biol..

[bib19] Hancock S., Finkelstein D., Adlard P. (2014). Glia and zinc in ageing and Alzheimer's disease: a mechanism for cognitive decline?. Front. Aging Neurosci..

[bib20] Hoeffel G., Ginhoux F. (2015). Ontogeny of tissue-resident macrophages. Front. Immunol..

[bib21] Hoeffel G., Chen J., Lavin Y., Low D., Almeida F.F., See P., Beaudin A.E., Lum J., Low I., Forsberg E.C. (2015). C-Myb(+) erythro-myeloid progenitor-derived fetal monocytes give rise to adult tissue-resident macrophages. Immunity.

[bib22] Jiang Y., Cowley S., Siler U., Melguizo D., Tilgner K., Browne C., Dewilton A., Przyborski S., Saretzki G., James W. (2012). Derivation and functional analysis of patient-specific induced pluripotent stem cells as an in vitro model of chronic granulomatous disease. Stem Cells.

[bib23] Kapellos T., Taylor L., Lee H., Cowley S., James W., Iqbal A., Greaves D. (2016). A novel real time imaging platform to quantify macrophage phagocytosis. Biochem. Pharmacol..

[bib24] Karlsson K., Cowley S., Martinez F., Shaw M., Minger S., James W. (2008). Homogeneous monocytes and macrophages from human embryonic stem cells following coculture-free differentiation in M-CSF and IL-3. Exp. Hematol..

[bib25] Kierdorf K., Erny D., Goldmann T., Sander V., Schulz C., Perdiguero E.G., Wieghofer P., Heinrich A., Riemke P., Hölscher C. (2013). Microglia emerge from erythromyeloid precursors via Pu.1- and Irf8-dependent pathways. Nat. Neurosci..

[bib26] Lee H., James W., Cowley S. (2017). LRRK2 in peripheral and central nervous system innate immunity: its link to Parkinson's disease. Biochem. Soc. Trans..

[bib27] Melief J., Koning N., Schuurman K., Van De Garde M., Smolders J., Hoek R., Van Eijk M., Hamann J., Huitinga I. (2012). Phenotyping primary human microglia: tight regulation of LPS responsiveness. Glia.

[bib28] Monier A., Adle-Biassette H., Delezoide A.L., Evrard P., Gressens P., Verney C. (2007). Entry and distribution of microglial cells in human embryonic and fetal cerebral cortex. J. Neuropathol. Exp. Neurol..

[bib29] Moore A.R., Zhou W.L., Jakovcevski I., Zecevic N., Antic S.D. (2011). Spontaneous electrical activity in the human fetal cortex in vitro. J. Neurosci..

[bib30] Muffat J., Li Y., Yuan B., Mitalipova M., Omer A., Corcoran S., Bakiasi G., Tsai L.-H., Aubourg P., Ransohoff R. (2016). Efficient derivation of microglia-like cells from human pluripotent stem cells. Nat. Med..

[bib31] O'Rourke J.G., Bogdanik L., Yáñez A., Lall D., Wolf A.J., Muhammad A.K., Ho R., Carmona S., Vit J.P., Zarrow J. (2016). C9orf72 is required for proper macrophage and microglial function in mice. Science.

[bib32] Ohgidani M., Kato T.A., Setoyama D., Sagata N., Hashimoto R., Shigenobu K., Yoshida T., Hayakawa K., Shimokawa N., Miura D. (2014). Direct induction of ramified microglia-like cells from human monocytes: dynamic microglial dysfunction in Nasu-Hakola disease. Sci. Rep..

[bib33] Palis J., Robertson S., Kennedy M., Wall C., Keller G. (1999). Development of erythroid and myeloid progenitors in the yolk sac and embryo proper of the mouse. Development.

[bib34] Pandya H., Shen M., Ichikawa D., Sedlock A., Choi Y., Johnson K., Kim G., Brown M., Elkahloun A., Maric D. (2017). Differentiation of human and murine induced pluripotent stem cells to microglia-like cells. Nat. Neurosci..

[bib35] Ransohoff R. (2016). Neuroinflammation: surprises from the sanitary engineers. Nature.

[bib36] Rezaie P., Dean A., Male D., Ulfig N. (2005). Microglia in the cerebral wall of the human telencephalon at second trimester. Cereb. Cortex.

[bib37] Russo I., Bubacco L., Greggio E. (2014). LRRK2 and neuroinflammation: partners in crime in Parkinson's disease?. J. Neuroinflammation.

[bib38] Schilling T., Nitsch R., Heinemann U., Haas D., Eder C. (2001). Astrocyte-released cytokines induce ramification and outward K+ channel expression in microglia via distinct signalling pathways. Eur. J. Neurosci..

[bib39] Schulz C., Perdiguero E., Chorro L., Szabo-Rogers H., Cagnard N., Kierdorf K., Prinz M., Wu B., Sten E., Pollard J. (2012). A lineage of myeloid cells independent of Myb and hematopoietic stem cells. Science.

[bib40] Schwartz M.P., Hou Z., Propson N.E., Zhang J., Engstrom C.J., Santos Costa V., Jiang P., Nguyen B.K., Bolin J.M., Daly W. (2015). Human pluripotent stem cell-derived neural constructs for predicting neural toxicity. Proc. Natl. Acad. Sci. USA.

[bib41] Shi Y., Kirwan P., Livesey F.J. (2012). Directed differentiation of human pluripotent stem cells to cerebral cortex neurons and neural networks. Nat. Protoc..

[bib42] Tamoutounour S., Guilliams M., Montanana Sanchis F., Liu H., Terhorst D., Malosse C., Pollet E., Ardouin L., Luche H., Sanchez C. (2013). Origins and functional specialization of macrophages and of conventional and monocyte-derived dendritic cells in mouse skin. Immunity.

[bib43] Tavian M., Peault B. (2005). Embryonic development of the human hematopoietic system. Int. J. Dev. Biol..

[bib44] van Wilgenburg B., Browne C., Vowles J., Cowley S. (2013). Efficient, long term production of monocyte-derived macrophages from human pluripotent stem cells under partly-defined and fully-defined conditions. PLoS One.

[bib45] Vanhee S., De Mulder K., Van Caeneghem Y., Verstichel G., Van Roy N., Menten B., Velghe I., Philippe J., De Bleser D., Lambrecht B.N. (2015). In vitro human embryonic stem cell hematopoiesis mimics MYB-independent yolk sac hematopoiesis. Haematologica.

[bib46] Villegas-Llerena C., Phillips A., Garcia-Reitboeck P., Hardy J., Pocock J. (2016). Microglial genes regulating neuroinflammation in the progression of Alzheimer's disease. Curr. Opin. Neurobiol..

[bib47] Zhang Y., Sloan S., Clarke L., Caneda C., Plaza C., Blumenthal P., Vogel H., Steinberg G., Edwards M., Li G. (2016). Purification and characterization of progenitor and mature human astrocytes reveals transcriptional and functional differences with mouse. Neuron.

