# Supplementary material for: A Highly Efficient Human Pluripotent Stem Cell Microglia Model Displays a Neuronal-Co-culture-Specific Expression Profile and Inflammatory Response
Source: Stem Cell Reports. 2017 Jun 9;8(6):1727–42. doi: 10.1016/j.stemcr.2017.05.017 (PMC5470330; doi:10.1016/j.stemcr.2017.05.017)
Supplement: Document S1. Supplemental Experimental Procedures, Figures S1–S6, and Tables S1 and S2 [file mmc1.pdf]

Stem Cell Reports, Volume *8*

Supplemental Information

A Highly Efficient Human Pluripotent Stem Cell Microglia Model Displays a Neuronal-Co-culture-Specific Expression Profile and Inflammatory Response

Walther Haenseler, Stephen N. Sansom, Julian Buchrieser, Sarah E. Newey, Craig S. Moore, Francesca J. Nicholls, Satyan Chintawar, Christian Schnell, Jack P. Antel, Nicholas D. Allen, M. Zameel Cader, Richard Wade-Martins, William S. James, and Sally A. Cowley

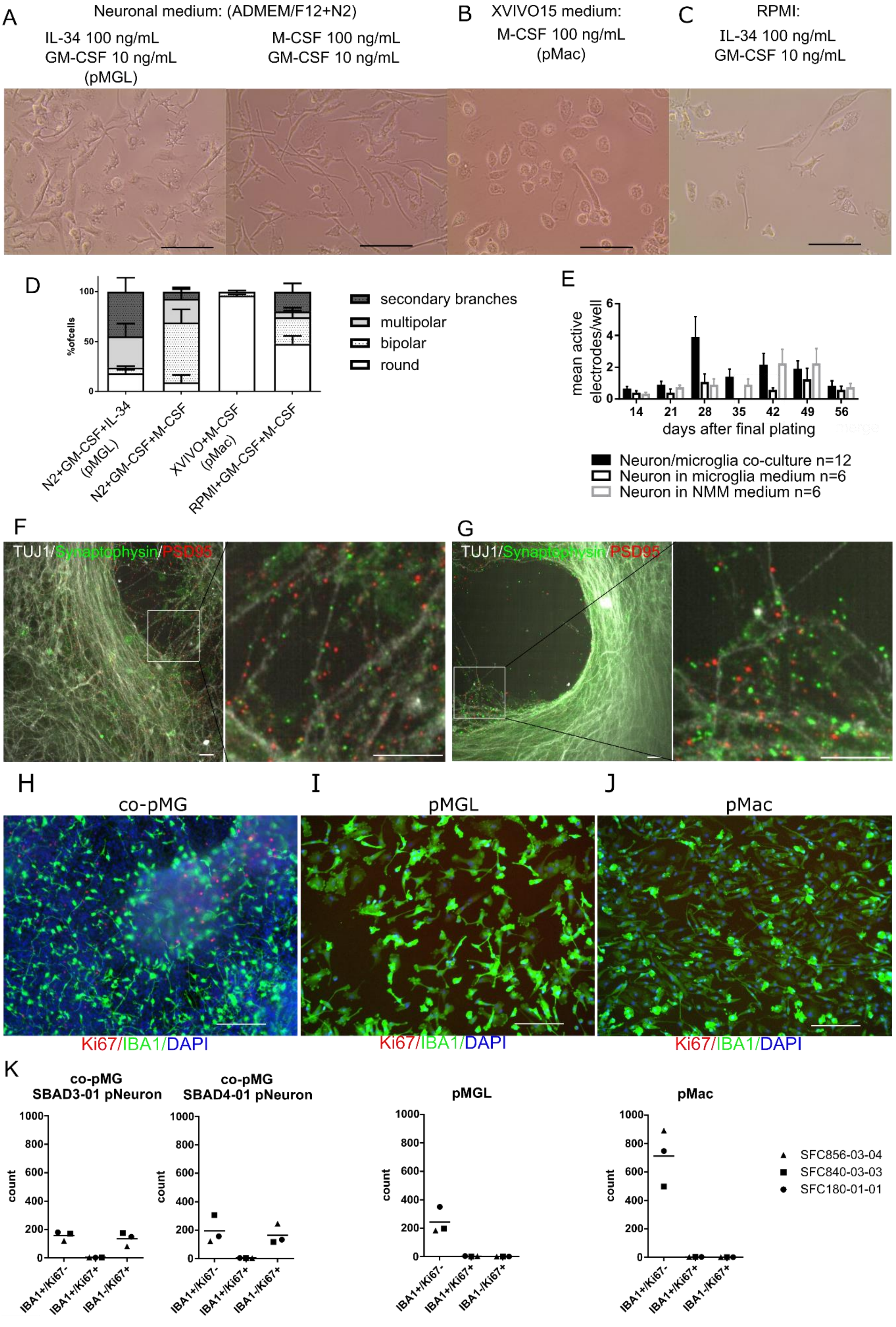

Figure S1 Further characterisation of macrophage and microglia cultures

(A-D) Identification of basal media and growth factors that promote ramified microglial morphology.pMacpre were differentiated for 17 days with different basal media and growth factors, as indicated. (A) Neuronal medium. (B) Our standard iPSC-macrophage medium (van Wilgenburg et al., 2013). (C) Medium described previously to promote microglia-like morphology in blood monocytes (Etemad et al., 2012). Representative phase contrast images of each condition show that ADMEM/F12 + N2 + 100 ng/mL IL-34 + 10 ng/mL GM-CSF promotes the most ramified microglia-like morphology. Scale bar 50 µm. (D) Quantification of morphology. Secondary branching was considered indicative of a ramified microglia-like morphology. Mean of 3 images per condition, error bars represent SEM. (E-G) Neuronal electrical functionality and synaptic markers in co-cultures. (E) Spontaneous electrical activity of cultures detected using a multi-electrode array (12 electrodes per well, n = number of wells, error bars represent SD). Neurons show spontaneous electrical activity from the beginning of co-culture, which increases modestly over an extended time period. Electrical activity is not inhibited by the presence of co-pMG. Note that electrical activity can only be detected from neurons that are in contact with the electrodes, and since the neurons form clumps, especially at longer culture times, not all replicate wells record activity. (F, G) The presynaptic marker Synaptophysin and the postsynaptic marker PSD95 can be detected in co-cultures (F) and neuron monoculture (G) (images taken at day 15 of co-culture, scalebar 20 µm). See also Video S1, showing calcium flux upon K^+ stimulation. (H-K) Continued Proliferation of iPSC-neuronal progenitors but rarely of microglia or macrophages. (H-J) Cultures were stained for the proliferation marker Ki-67 (Red) and IBA1 (macrophages/microglia, green) after 3 weeks. (H) Co-culture of SFC840-03-01 microglia with SBAD3-01 neurons. (I) SFC840-03-03 macrophages cultured in microglia medium. (J) SFC840-03-03 macrophages cultured in macrophage medium. Scale bar 100 µm. (K) Quantification of IBA1 and Ki67 signal in microglia/macrophages of 3 iPSC lines in co-culture with 2 different pNeurons (SBAD3-01, SBAD4-01) or in monoculture. In neuronal clusters the DAPI signal was too dense for quantification of the number of neurons, but is expected to be >3000 neurons/image.

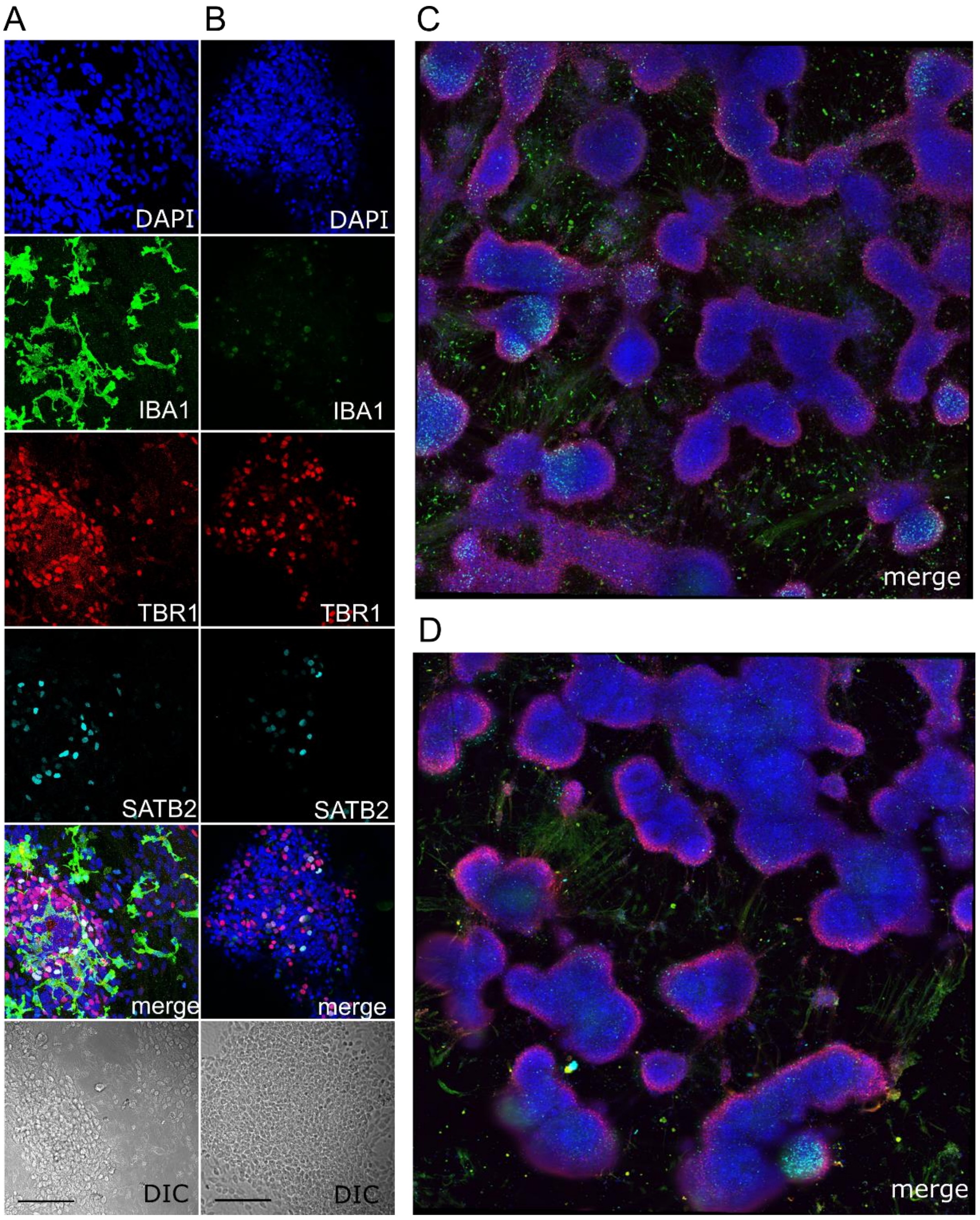

Figure S2 iPSC-microglia-neuronal co-cultures express deep layer (TBR1) and upper layer (SATB2) cortical markers and are stable for extended periods of co-culture (Relates to Figure 1)

(A) 14 day co-culture. (B) 14 day neuron-only culture. (C) 39 day co-culture (IBA1, SATB2, TBR1, DAPI). (D) 39 day neuron-only culture. Scale bar 50 µm.

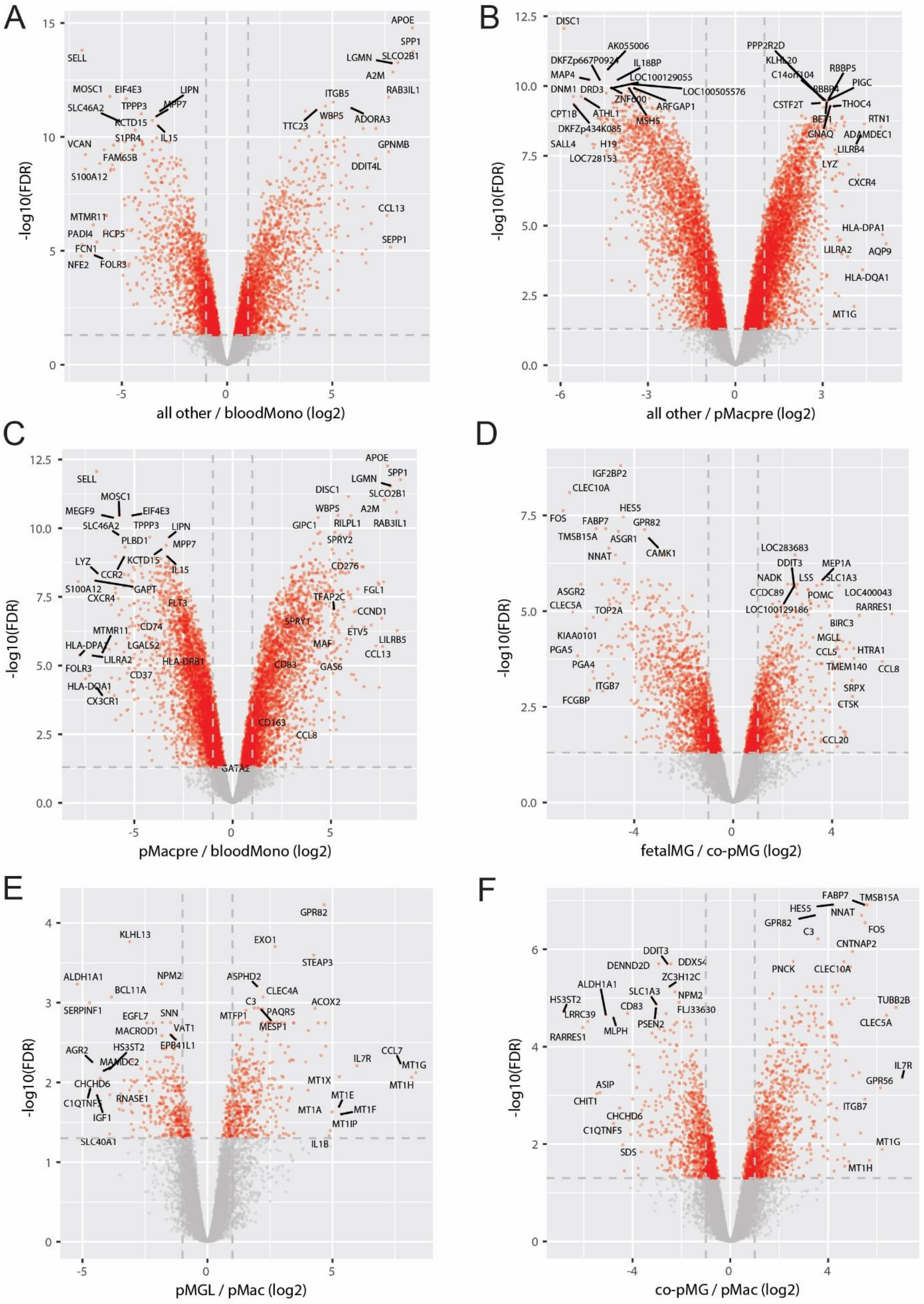

Supplementary Figure S3 Differential expression analyses (Relates to Figure 2)

Volcano plots shows genes differentially expressed between (A) bloodMono vs all other samples (to explore PC2 in Figure 2A); (B) pMacpre vs all other samples (to explore PC1 in Figure 2A); (C-F) other comparisons of interest

as indicated. Horizontal dashed lines indicate an adjusted p- value of 0.05. Vertical dashed lines indicate a two-fold difference in expression.

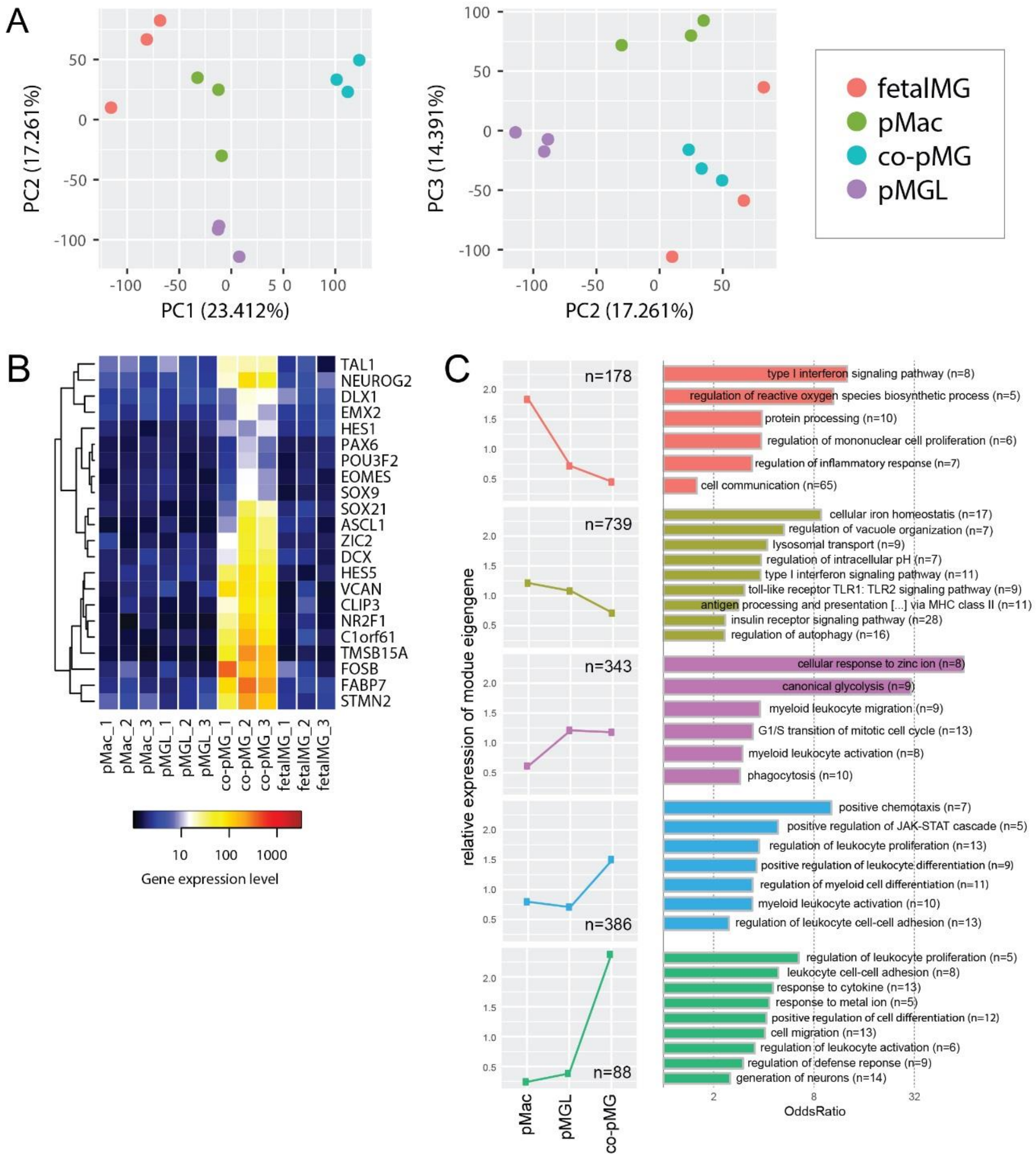

Supplementary Figure S4 Transcriptomics analysis of macrophage populations (Relates to Figure 2)

(A) PCA analysis of gene expression. Inspection of the proportion of variance scree plot identified three important components (data not shown). GO analysis revealed genes positively loading the first principle component (PC1, 23.4% of variance) to contain annotation categories associated with neural cells (data not shown). This signature is associated with co-pMG which, given their close association with neural cells whilst in co-culture, likely reflects a low level of neural cell derived contamination. Reassuringly, sample projection based on PC2 and PC3 (together explaining 31.7% of variance, right panel) demonstrated the similarity of the co-pMG and fetalMG samples. (B) The heatmap shows examples of neural genes that positively contribute to PC1 (A). (C) Neuronal co-culture induces a microglia-like differentiation signature in iPSC derived cells. The figure shows k-means cluster profiles (left, line plots) and associated enriched biological processes (selected GO categories, adjusted p-value < 0.05) (right, bar graphs). Weakly detected genes were excluded from the analysis to limit the impact of transcripts deriving from the apparent low-level neural cell derived contamination of the co-pMG sample. Genes with significantly variable expression (adjusted $p < 0.05$) between pMac, pMGL and co-pMG were used as the input for the kmeans clustering. Salmon red panel shows pathways strongly downregulated in co-pMG versus iMac,

green panel shows pathways strongly upregulated in co-pMG versus pMac, the intermediate panels show pathways moderately up- (blue, purple) or downregulated (green-grey) in co- pMG. In the line graph panels n = number of microarray probes, in the bar graph panels n = number of genes. Further details of the analyses are given in the Supplementary Experimental Procedures.

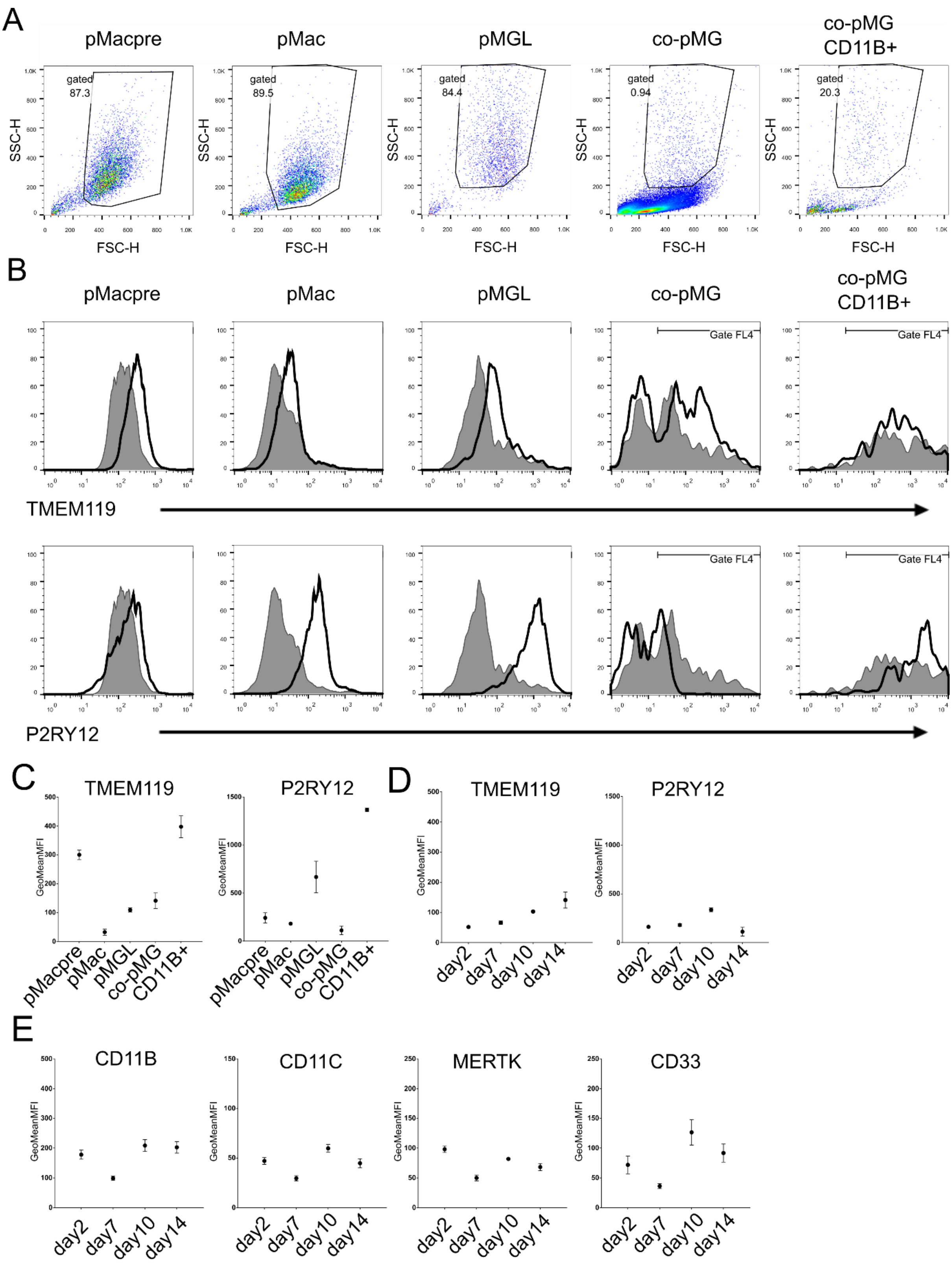

Figure S5 Additional flow cytometry (Relates to Figure 4)

(A) Forward Scatter FSC/Side Scatter SSC gating for Figure 4 and Figure S6. (B) FACS plots of microglia marker (black line) and 2nd antibody-only staining (grey) for SFC856-03-04 shows staining for TMEM119 and P2RY12 in monoculture but in co-culture non-specific background staining for 2nd antibody-only is apparent. (C) Mean Fluorescence Intensity of microglia markers (mean and SEM of 3 genetic backgrounds) in the different macrophage populations (D, E) Time-course of co-culture for microglia and monocyte/macrophage markers.

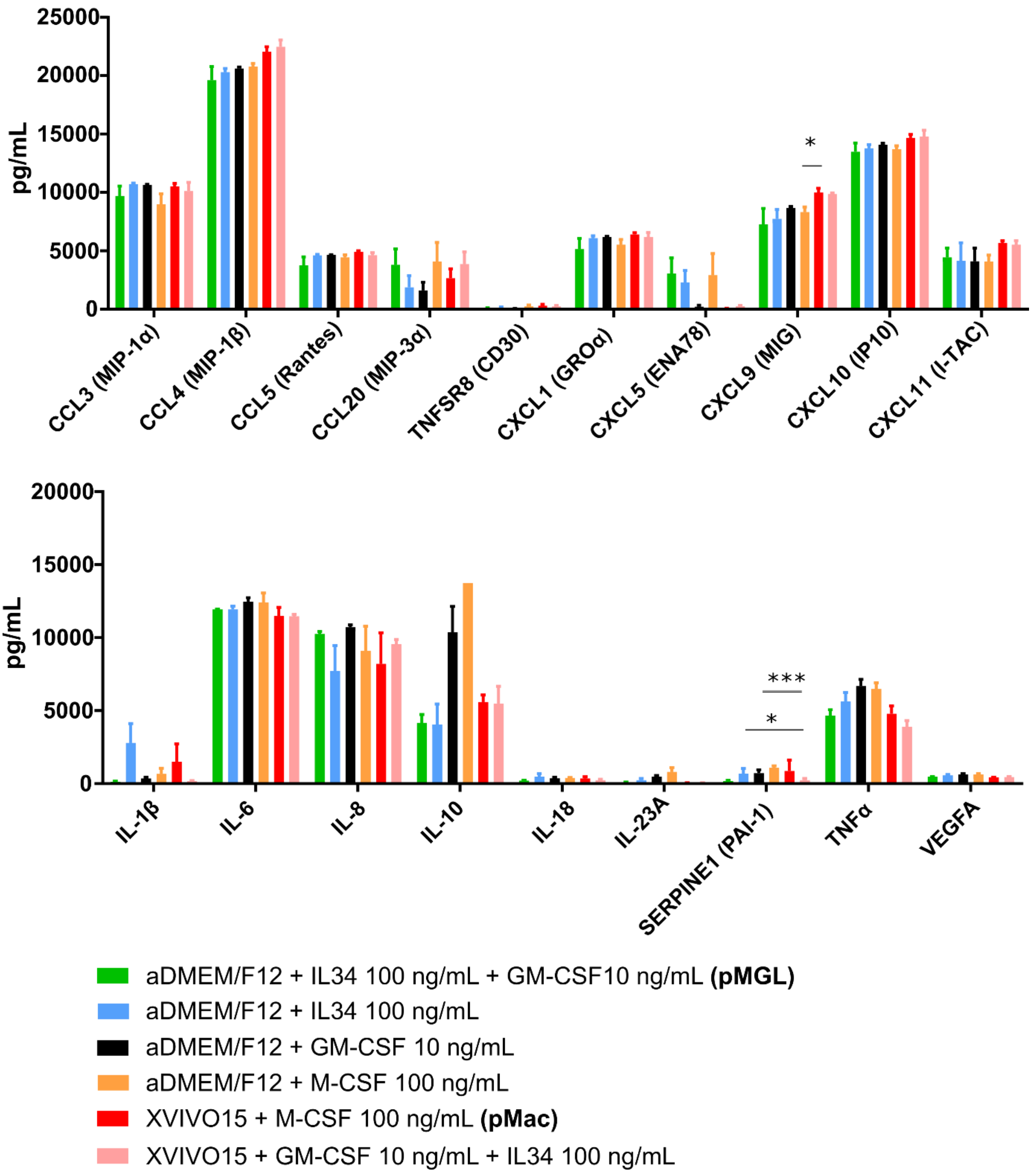

Figure S6 Effect of basal medium and cytokines on the inflammatory response to LPS/IFNγ (Relates to Figure 7)

Luminex multiplex array. Mean ± SEM of 3 biological replicates. Statistical analysis was performed with one way ANOVA followed by Tukey’s multiple comparison test.

Table S1: Details of cells and materials used in this study

iPSC lines used in this study (All from disease-free donors)					
ID of fibroblast	ID of iPSC clone	Gender	Age of Biopsy (years)	Reprogramming method	iPSC clone characterised
SF180	SFC180-01-01	female	60	Cytotune1	Haenseler in submission
SF840	SFC840-03-01	female	67	Cytotune1	(Fernandes et al., 2016)
	SFC840-03-03				
SF856	SFC856-03-04	female	78	Cytotune1	Haenseler in submission
SBAD3	SBAD3-01	female	36	Cytotune1	Melguzo in submission
SBAD4	SBAD4-01	male	80	Cytotune1	Melguzo in submission
AH016	AH016-3	male	80	rv SO^{3}KMN	(Sandor et al., 2017)
	AH016-3 Lenti_RFP_IP (11 copy)				This study

Microglia medium (for pMGL, co-pMG and pNeuron)				
	Final conc	Stock conc	Supplier	Cat no.
Advanced DMEM/F12	1x	1x	Life Technologies	12634-010
N2 supplement	1x	100x	Life Technologies	17502-048
GlutaMAX™	2mM	200mM	Life Technologies	35050-061
2-mercaptoethanol	50µM	50mM	Life Technologies	31350-010
Pen/Strep	50U/mL	100x	Life Technologies	17502-048
IL-34	100ng/mL	100ug/mL	Peprotech	200-34
GM-CSF	10ng/mL	10ug/mL	Life Technologies	PHC2013
Growth-factor reduced Matrigel (undefined product for coating plate for co-pMG and pNeuron)	83-fold dilution of supplied stock		Scientific Laboratory Supplies	354277

Neuronal maintenance medium (NMM) (for differentiation until start of co-culture and for pNeuron (Shi et al., 2012))				
	Final conc	Stock conc	Supplier	Cat no.
Neurobasal	1x	1x	Life Technologies	21103-049
Advanced DMEM/F12	1x	1x	Life Technologies	12634-010
B27 supplement	0.5x	100x	Life Technologies	17504-044
N2 supplement	0.5x	100x	Life Technologies	17502-048
GlutaMAX™	2mM	200mM	Life Technologies	35050-061
2-mercaptoethanol	50µM	50mM	Life Technologies	31350-010
Pen/Strep	50U/mL	100x	Life Technologies	17502-048
Insulin	5ug/mL		Sigma	I6634
Growth-factor reduced Matrigel (undefined product for coating plate)	83-fold dilution of supplied stock		Scientific Laboratory Supplies	354277

Macrophage differentiation medium (for pMac (van Wilgenburg et al., 2013))				
	Final conc	Stock conc	Supplier	Cat no.
X-VIVO 15	1x	1x	Lonza	BE04-418
GlutaMAX™	2mM	200mM	Life Technologies	35050-061
2-mercaptoethanol	50µM	50mM	Life Technologies	31350-010

Pen/Strep	50U/mL	100x	Life Technologies	17502-048
M-CSF	100ng/mL	100µg/mL	Gibco	PHC 9501

Composition of N2 and B27 supplements
(*components that are potentially immunosuppressive / stress buffers)

**N2 Supplement**	Conc in 100x	**B27 supplement**	Conc in 50x
Human Transferrin (Holo)	1mM	DL Alpha Tocopherol Acetate	Concentrations not given by manufacturer
Insulin Recombinant Full Chain*	0.086mM	DL Alpha-Tocopherol	
Progesterone*	0.002mM	Vitamin A (acetate)	
Putrescine*	10mM	BSA, fatty acid free Fraction V	
Selenite	0.003mM	Catalase*	
		Human Recombinant Insulin*	
		Superoxide Dismutase*	
		Corticosterone*	
		D-Galactose	
		Ethanolamine HCl	
		Glutathione (reduced)	
		L-Carnitine HCl	
		Linoleic Acid	
		Linolenic Acid	
		Progesterone*	
		Putrescine 2HCl*	
		Sodium Selenite	
		T3 (triodo-I-thyronine)	

Antibodies used for Immunocytochemistry

Primary	Species/ clonality	Manu-facturer	Cat. No.	Secondary	Fluorophore	Manu-facturer	Cat. No.
IBA1	goat/ poly	abcam	ab5076	donkey- αgoat	Alexa488	Thermo Fisher	A11055
TUJ1	mouse/ mono	Covance	MMS-435P	donkey- αmouse	Alexa647	Thermo Fisher	A10042
TUJ1	rabbit	Covance	MRB-435P	Donkey- αrabbit	Alexa568	Thermo Fisher	A31571
GFAP	rabbit/ poly	DAKO	ZO334	donkey- αrabbit	Alexa568	Thermo Fisher	A31571
TBR1	rabbit/ poly	Abcam	ab31940	donkey- αrabbit	Alexa568	Thermo Fisher	A31571
SATB2	mouse/ mono	Abcam	ab51502	donkey- αmouse	Alexa647	Thermo Fisher	A10042
NESTIN	mouse/ mono	Abcam	ab22035	donkey- αmouse	Alexa647	Thermo Fisher	A10042

PAX6	rabbit/ poly	Covance	PRB-278P	donkey-αrabbit	Alexa568	Thermo Fisher	A31571
SYNAPTO-PHYSIN	guinea pig/ poly	Synaptic Systems	101 004	goat-α-guinea pig	Alexa488	Thermo Fisher	A11073
PSD95	mouse/ mono	Thermo Fisher	MA1-045	donkey-αmouse	Alexa647	Thermo Fisher	A10042
Ki67	mouse/ mono	Merck Millipore	MAB4190	donkey-αmouse	Alexa647	Thermo Fisher	A10042
TMEM119	rabbit/ poly	abcam	ab185333	donkey-αrabbit	Alexa568	Thermo Fisher	A31571
P2RY12	rabbit/ mono	abcam	ab188968	donkey-αrabbit	Alexa568	Thermo Fisher	A31571
IgG	rabbit/ poly	abcam	ab27478	donkey-αrabbit	Alexa568	Thermo Fisher	A31571
MERTK	mouse/ mono	abcam	Ab52591	donkey-αmouse	Alexa647	Thermo Fisher	A10042
IgG1	Mouse/ mono	AbD serotec	MCA928	donkey-αmouse	Alexa647	Thermo Fisher	A10042

Fluorophore conjugated antibodies used for Flow Cytometry

Marker	Fluorophore	Isotype	Manufacturer	Cat. No. marker	Cat. No. isotype control
CD11b	APC	mouse IgG1-Κ	Biolegend	301309	400119
CD11C	FITC	mouse IgG2a	ImmunoTools	21487113	21335023
CD14	PE	mouse IgG1	ImmunoTools	21620144	21335014
CD45	APC	mouse IgG1	ImmunoTools	21270456	21275516
HLA-DR	FITC	mouse IgG2a	ImmunoTools	21278993	21335023
CX3CR1	APC	rat IgG2b-Κ	Biolegend	341609	400611
CD33	APC	mouse IgG1	eBioscience	17-0338-42	17-4717-41
MERTK	Alexa647	mouse IgG1-Κ	Biolegend	367606	400130

Primers used for qRT-PCR of microglia markers

	Forward primer sequence: (5' to 3')	Reverse primer sequence: (5' to 3')	supplier
C1QA	GTGACACATGCTCTAAGAAG	GACTCTTAAGCACTGGATTG	Sigma Aldrich
GAS6	CGAAGAAACTCAAGAAGCAG	AGACCTTGATCTCCATTAGG	Sigma Aldrich
GPR34	GAAGACAATGAGAAGTCATACC	TGTTGCTGAGAAGTTTTGTG	Sigma Aldrich
PROS1	AAAGATGTGGATGAATGCTC	TCACATTCAAAATCTCCTGG	Sigma Aldrich
MERTK	AGGACTTCCTCACTTTACTAAG	TGAACCCAGAAAATGTTGAC	Sigma Aldrich
P2RY12	AAGAGCACTCAAGACTTTAC	GGGTTTGAATGTATCCAGTAAG	Sigma Aldrich
TMEM119	AGTCCTGTACGCCAAGGAAC	GCAGCAACAGAAGGATGAGG	Sigma Aldrich
TREM2	TCTGAGAGCTTCGAGGATGC	GGGGATTTCTCCTTCAAGA	Sigma Aldrich
18S	Sequences not provided by supplier		Eurogentec

Table S2 Initial screen with Proteome Profiler™ Human XL Cytokine Array (Relates to Figure 7)

Released factor	pMac unstim	pMac LPS/IFNγ	co-pMG unstim	co-pMG LPS/IFNγ
Adiponectin	2870	1970	4640	2960
Aggrecan	9375	7010	7080	9105
**Angiogenin**	21250	14100	106500	65350
Angiopoietin-1	2365	2540	4475	5655
Angiopoietin-2	4655	4095	7020	8695
BAFF	4385	3485	4870	10725
BDNF	3010	1510	3855	4140
**C5/C5a**	2420	12600	4645	19300
CCL2 (MCP-1)	116500	144500	148000	143500
**CCL3/4 (MIP-1α/MIP-1β)***	3260	155500	3230	18200
**CCL5 (RANTES)***	3120	115500	3930	4665
**CCL7 (MCP-3)***	3975	48200	6505	51700
CCL17 (SDF-1α)	4525	6120	9840	10320
CCL17 (TARC)	3130	2035	3255	3070
**CCL19 (MIP-3β)***	2435	14350	2765	9790
**CCL20 (MIP-3α)***	1760	99650	2570	6895
**CD14**	31450	24200	46350	38900
CD40Ligand (CD154)	3425	2340	4855	4795
Chitinase 3-like 1	250500	236000	229000	209000
Complement Factor D	16100	10940	10130	13200
C-Reactive Protein	2665	5360	5040	7775
Cripto-1	2360	2320	3510	3080
**CXCL1 (GRO-α)***	3515	101900	62050	102100
CXCL4 (PF4)	2045	162	1895	2900
**CXCL5 (ENA-78)***	2225	3960	166000	102000
**CXCL9 (MIG)***	2935	128000	3620	55100
**CXCL10 (IP-10)***	3720	172500	3620	156500
**CXCL11 (I-TAC)***	2970	231000	1825	119000
Cytostatin C	35500	22950	55400	34800
Dkk-1	2475	3135	2780	2675
DPPIV (CD26)	21700	47700	42050	16045
**EGF**	3455	2880	6240	6510
**EMMPRIN (CD147)**	12050	20800	21000	4105
Endoglin (CD105)	8365	8665	9940	7470
**Fas Ligand (CD178)**	2970	1925	3015	5575
**FGF basic**	3850	9590	6925	11950
**FGF-7**	2275	966	3385	5385
**FGF19**	14900	24600	24250	35250
**Flt-3 Ligand**	1915	1355	3395	1520
**G-CSF**	1330	4840	2765	6175
GDF-15	7325	10570	6195	5790
**GM-CSF(spiked in co-pMG)**	4335	4860	31950	13350
**Growth hormone**	1340	1340	4215	1910
**HGF**	2245	7675	4780	7715
**ICAM-1 (CD54)**	10900	26600	30950	38400
**IGFBP-2**	2135	1147	124500	91850
IGFBP-3	3540	4555	8625	7430
**IL-1-ra**	15745	68350	47400	25300
**IL-1α**	4585	4540	6940	9825
IL-1β*	3535	6535	4740	5630
IL-2	2995	3025	4645	2610
IL-3	1565	1102	2335	1930
**IL-4**	4970	8225	7430	9435
IL-5	2190	909	2330	2055

**IL-6***	4440	107500	6840	102000
**IL-8 (CXCL8)***	63150	122000	149000	136000
**IL-10***	4530	19900	7565	47850
**IL-11**	5835	7365	9115	10850
**IL-12p70**	2915	4310	4290	5660
**IL-13**	2135	2525	4410	6250
**IL-15**	2690	4040	3085	6725
**IL-16**	2145	2405	2715	5860
**IL-17A**	14600	33250	18700	28150
**IL-18 Bpa***	2180	10600	3125	9030
**IL-19**	2635	2645	3145	39600
IL-22	5120	9720	6410	8270
**IL-23***	1830	13250	1468	5400
IL-24	2355	4260	3705	4155
IL-27	3760	9640	2390	5680
IL-31	1770	2089	2735	2510
IL-32α/β/γ	2510	4625	2585	4175
**IL-33**	754	2125	2675	4035
**IL-34 (spiked in co-pMG)**	2130	806	14100	3980
INFγ (spiked in LPS/IFNγ)*	8420	109000	8100	104500
Kallikrein 3	1695	2840	3860	1747
Leptin	3430	2940	3275	4620
LIF	2555	1565	1750	4270
**Lipocalin-2**	7785	8250	2320	3675
**M-CSF (spiked in pMac)**	137500	133500	5250	10725
**MIF***	20550	11450	70800	72250
**MMP-9**	56950	55100	76150	19100
Myeloperoxidase	2530	322	1605	1641
Osteopontin	107750	106000	111000	105750
**PDGF-AA**	9715	19200	5195	7080
**PDGF-AB/BB**	5615	4865	1200	1515
**Pentraxin-3**	5150	3310	12350	19050
RAGE	2320	825	2880	3295
**RBP4**	315500	355000	4180	4565
Relaxin-2	2590	2070	1510	3035
Resistin	5670	5420	7495	5515
**SerpinE1 (PAI-1)***	4630	12000	104000	87350
SHBG	3675	4675	5120	4790
ST2	2680	1930	2255	3760
TFF3	5340	3845	2950	3865
TfR (CD71)	3380	2025	3970	4475
TGF-α	2865	2230	3735	3980
**TNFRSF8 (CD30)***	2445	645	4800	2090
**TNF-α***	3645	103500	1960	8195
Thrombospondin-1	1455	-130	2950	769
**uPAR (CD87)**	6895	51050	14250	6130
**VEGF***	1590	147	36350	3315
Vitamin D BP	8660	10600	6240	7865

Legend Table S2

Results were quantified with Image Studio Lite. Numbers show the mean luminescence signal of two dots per factor (n=2). Factors that are substantially differentially released between pMac and co-pMG or upon stimulation with LPS/IFNγ are in bold font. Negative values are where measurement is below background luminescence. * are followed up in Figure 7.

Legend Table S3 Transcriptomics data and differential expression results

The Excel file contains the normalised probe level expression data and the full results of the differential expression analyses presented in Supplementary Figure S3. Further details of these analyses are given in the Supplementary Experimental Procedures.

Supplemental Experimental Procedures

iPSC lines

The derivation and characterisation of the iPSC lines used in this study is described elsewhere (Fernandes et al., 2016; Sandor et al., 2017) Haenseler, submitted, Melguzo in preparation), see Table S1. All lines were derived from dermal fibroblasts from disease-free donors recruited through StemBANCC (SF180, SF856) (Morrison et al., 2015), or the Oxford Parkinson's Disease Centre (SF840, AH016): participants were recruited to this study having given signed informed consent, which included derivation of hiPSC lines from skin biopsies (Ethics Committee: National Health Service, Health Research Authority, NRES Committee South Central, Berkshire, UK, who specifically approved this part of the study (REC 10/H0505/71), or from fibroblasts purchased from Lonza (SBAD3, SBAD4), who provide the following ethics statement: 'These cells were isolated from donated human tissue after obtaining permission for their use in research applications by informed consent or legal authorization.' iPSC were cultured in mTeSR™1 (StemCell Technologies), on hESC-qualified Matrigel-coated plates (BD), passaging as clumps using 0.5 mM EDTA in PBS (Beers et al., 2012). Large-scale SNP-QCed batches were frozen at p15-25 and used for experiments within a minimal number of passages post-thaw to ensure consistency.

Generation and characterisation of the RFP expressing iPSC line AH016-3 Lenti_IP_RFP

To generate an iPSC line constitutively expressing RFP, AH016-3 was transduced with a second generation SIN lentiviral vector (LV-EF1a-RFP-IRES-Puromycin^{R}). Cells were kept under continuous puromycin selection (2 µg/mL: a concentration sufficient to kill untransduced cells). For single cell cloning AH016-3-RFP were plated at 10^4 per 10 cm dish on mitotically-inactivated mouse embryonic fibroblast feeder cells (MEF; outbred Swiss mice established and maintained at the Department of Pathology, Oxford (Chia, Achilli, Festing, & Fisher, 2005; Gardner, 1982)) on gelatin-coated tissue culture plates in hESC medium (KO-DMEM, 2 mmol/L GlutaMAX 100 mmol/L non-essential amino acids, 20% serum replacement (KO-SR), and 8 ng/mL basic fibroblastic growth factor (FGF2)), supplemented with 10 µmol/L Y-27632 on the day of the plating. After 7 days of expansion, individual single-cell colonies were picked manually onto a matrigel coated 96 well plate in mTeSR^{TM}1. Number of lentiviral integrants per clone was quantified using digital droplet PCR (ddPCR) copy number variation analysis (Bio-Rad QX200) according to manufacturer's protocol. Briefly, 2 µl of EcoRI-digested genomic DNA at 100 ng/µl was used with the EvaGreen Super Mix and 100 nM forward and reverse primers. The following RFP primers were used: JB-111 (5' - ATGCAGAAGAAAACACGCGG - 3') and JB-112 (5' - CCGGGCATCTTGAGGTTCTT - 3'). PCR primers for the *MYB* gene, were used as endogenous control: JB-71 (5' – ACAGGAAGGTTATCTGCAGGAGTCT – 3') and JB-72 (5' – AGTGGCAGGGAGTTGAGCTGTA - 3'). The iPSC clone used in this study has 11 lentiviral integrants.

Immunofluorescence

Cells were fixed with 4% paraformaldehyde (PFA) in PBS for 10 min, permeabilised with 0.3% Triton X-100 in PBS and blocked with 10% normal donkey serum (Sigma) for 1 hr, then incubated with primary antibodies in PBS, 5% normal donkey serum and 0.1% Triton X-100 overnight, washed 3 times with PBS and 0.3% Triton X-100, incubated with secondary antibodies in PBS 0.1% Triton X-100 and 5% normal donkey serum for 90 min, washed 3 times with PBS and 0.3% Triton X-100, stained with DAPI, washed once with PBS, overlaid with PBS and imaged with an EVOS fl auto microscope (AMG), FV1200 (Olympus) confocal microscope, OperaPhenix (PerkinElmer) or an IN Cell Analyzer 6000 (GE).

The antibodies used are listed in Table S3.

Transcriptome sample preparation and analysis

30 mL of peripheral blood was collected from 3 healthy adult volunteers, according to University of Oxford OHS policy document 1/03, with signed informed consent. PBMCs were isolated after density gradient centrifugation with Ficol-Paque PLUS (17-1440-03, GE Healthcare), and monocytes (bloodMono) were extracted with CD14 MACS beads (130-050-201, Miltenyi). iPSC-derived macrophage precursors (pMacpre), from 3 control lines, were collected and immediately lysed for RNA isolation. From the same harvest, cells were set up in macrophage differentiation medium for 2 weeks to obtain pMac, and in microglia medium to obtain pMGL (lysed directly in the well after 2 weeks to obtain RNA), or resuspended in microglia medium, added to SBAD3-01 neurons and differentiated to co-pMG for 2 weeks. Then co-culture was dissociated to a single cell suspension with StemPro accutase (StemCell technologies), and any remaining adherent microglia gently lifted with a cell scraper. Cells were passed through a 70 µm cell strainer (352350, BD Bioscience), then co-pMG were selected from the co-culture with CD11b MACS beads (130-093-634, Miltenyi Biotec) and lysed immediately for RNA isolation. RNA was extracted from lysates using an RNeasy kit (Qiagen) for Illumina HT12v4 transcriptome array analysis. For qPCR, additional samples were blood monocyte-derived macrophages (bloodMac), which were differentiated on tissue-culture-treated plates, for 1 week in macrophage medium and compared directly to the same donors' bloodMono (lysed straight after CD14 bead selection) and to pMac differentiated for 1 week.

RNA from human microglia was obtained from 3 individual human fetal samples (at pre-myelinating gestational ages of fetalMG_1 20, fetalMG_2 23, fetalMG_3_15 weeks) and one human adult sample, according to Durafourt et al. (Durafourt et al., 2013). Briefly, microglia were cultured *ex vivo* in DMEM supplemented with 5% FBS for 5-7 days prior to RNA isolation using standard Trizol^{TM} methods. All procedures related to the use of these cells followed established institutional (McGill University, Montreal, QC, Canada) and Canadian Institutes of Health Research guidelines for the use of human cells. A further sample of adult microglia RNA was also obtained from directly isolated human surgical brain material (age 51; UK: Re: An Investigation of Novel Proteins and Biomarkers in Surgically-Resected Tissue from Patients with Epilepsy. R&D Ref: 10815; REC Ref: 14/EE/1098; IRAS No: 144065). Brain material was papain-treated to obtain single cells, then panned with CD11b MACS beads and the positive population lysed immediately for RNA extraction, within 4 hours of surgical removal.

Microarray data were pre-processed with the Bioconductor beadarray package (Dunning et al., 2007) using the "neqc" method to normalise expression levels within and between samples. Annotations were sourced from the Bioconductor illuminaHumanv4.db package: probes with an assigned quality of "No match" were excluded from down-stream analysis. Following inspection of the data only the top two-thirds of probes (by maximum expression level) were retained for further analysis being considered to represent "expressed genes". PCA analysis of the normalised, scaled expression matrix was performed using the R “prcomp” function. Differential expression analysis was performed using the Limma Bioconductor package (Ritchie et al., 2015). The Benjamini-Hochberg (BH) multiple testing correction procedure was used to compute adjusted p-values.

K-means clustering analysis of gene expression in pMac, pMGL and co-pMG was based on a set of 1734 probes with high (>=1000 in at least 2 replicates) and significantly variable (overall F-test, Limma, BH adjusted p value < 0.05) expression in these samples. K-means clustering was performed on the matrix of mean-scaled gene expression levels (replicate samples first combined by median averaging) using the R "kmeans" function (nstarts=10000, iter.max=10000). Selection of cluster number was guided by scree-plot analysis of within-cluster sum of squares (not shown). The Bioconductor GOStats package (Falcon and Gentleman, 2007) was used to identify significantly over-enriched GO biological processes within each cluster (conditional test, p-value cutoff 0.01, gene universe comprised of the top two-thirds "expressed genes", adjusted p-value < 0.05).

RNA-seq data for human astrocytes, endothelial cells myeloid cells, neurons and oligodendrocytes (Zhang et al., 2016) was retrieved from GEO (GSE73721). Per-gene expression levels (upper-quartile normalised TPMs) were quantitated using Salmon (Patro et al., 2017) with a quasi index (31bp k-mers) built from human coding sequences (Ensembl version 84 hg38 annotations). Gene expression levels for the RNA-seq and microarray data were merged, subject to a robust quantile normalisation (R package “preprocessCore”, weighted to be informed only the RNA-seq samples) and log2(n+1) transformed. Non-negative matrix factorisation was applied to the values from the Zhang. et. al. samples (filtered to exclude genes below the 25th expression quantile). Five meta-genes were identified using the R package “NNLM” (method=”scd”, rel.tol=-1, max.iter=100K, loss=”mkl”). Samples were hierarchically clustered by meta-gene expression level (manhattan distance, complete agglomeration), the R package “pvclust” was used to calculate approximately unbiased p-values for the clusters (nboot=100K) and leaf order was optimised using the R package “cba”.

Reverse transcription and qRT PCR

RNA was reverse transcribed using High-Capacity RNA-to-cDNA™ Kit (Thermo Fisher). Qunatitative real time PCR was performed with *Power* SYBR® Green PCR Master Mix (Thermo Fisher) on a StepOnePlus™ Real-Time PCR System. Primers used are listed in Table S5.

Flow cytometry

Co-pMG were isolated with CD11b magnetic beads (MACS®, Miltenyi Biotec) as described for the transcriptome sample preparation. Pilot experiments to detach macrophages from the tissue culture plate with accutase, in direct comparison with our previous protocol of cold 5 mM EDTA/12 mM Lidocaine (Carter et al., 2009), showed no substantive difference in surface marker levels, so accutase was used as it lifts the cells much more rapidly, thereby minimising cellular change/damage. Where relevant, macrophages were also subjected to CD11b bead treatment. Freshly harvested macrophage precursors were stained directly, after passage through a 40 µM cell strainer (BD Bioscience). The antibodies used are listed in Table S4. Isotype control antibodies used were from the same company with the same fluorophore at the same concentration (van Wilgenburg et al., 2013).

Live imaging

AH016-3 Lenti-IP-RFP-microglia were co-cultured with SFC840-03-01 cortical neurons in matrigel-coated 96-well black/clear bottom plates (Costar, 3603). RFP signal was used to visualize microglia in co-culture. Images of RFP signal and phase were taken every 5 minutes for 5 hours (2 videos/well). Microglial movement was manually tracked with ImageJ and tracks were analysed with Chemotaxis and Migration Tool Version 2.0 (Ibidi). Microglia positions were determined by marking all microglia with Image J. Manual counting and distance to next neighbour was calculated from this data with R.
To check for proinflammatory microglial morphology, co-cultures were treated with 100 ng/mL LPS imaged every 5 minutes for 17 hr respectively 20 hr. To visualise phagocytic activity, pHrodo Green zymosan yeast bioparticles (ThermoFisher, P35365) were added at 50 µg/mL. pHrodo dyes fluoresce at low pH, ie, as the phagosome is progressively acidified after uptake of the particle in microglia (Kapellos et al., 2016). Wells were imaged every 10 minutes for 5 hours.

Calcium imaging was performed with Fluo-4 Direct™ Calcium assay kit (Thermo Fisher). Cells were cultured in 100 µl microglia medium, 100 µl of assay reagent was added to the medium and cells were incubated for 1 hr at 37ºC. All medium was then removed and replaced with 200 µl Tyrode's solution supplemented with 6 mM potassium to activate the neurons. Neurons were then imaged every 3 seconds for 2 minutes. Live imaging was performed with an EVOS™ FL Auto imaging system (Thermo Fisher) with a humidified onstage incubator set to 37ºC, 5% CO_2.

Cytokine/chemokine release measurements

Proteome profilerXL (R&D systems) was used to identify candidate cytokines that are upregulated upon stimulation or differentially expressed/released between standard iPSC-derived macrophages and co-culture microglia. SFC180-01-01 pMac, or SFC180-01-01 co-pMG in co-culture with SBAD4-01 pNeurons, were

stimulated, after 3 weeks of culture, for 18 hours with 100 ng/mL LPS and 100 ng/mL IFNγ. Supernatant was then collected and applied to the proteome profiler membranes according to manufacturer's instructions. Luminescence was captured with a GeneSnap Gel documentation system (SynGene) and signal was quantified with Image Studio Lite Version 5.2 (LI-COR).

22 cytokine/chemokine targets were assayed with a ProcartaPlex™ Custom Panel (eBioscience). pNeuron were co-cultured for 2 weeks with 3 different co-pMG, meanwhile iMac from the same lines were cultured in parallel monocultures in either macrophage medium or as pMGL in microglia medium, in a 96 well plate. Cells were then stimulated with 100 ng/mL LPS and 100 ng/mL IFNγ or with medium only for 18 hr, supernatant was collected, centrifuged and analysed with multiplex beads, according to manufacturer's instructions, with a Luminex 100 Bio-Plex system (BioRad).

Supplemental References

Beers, J., Gulbranson, D., George, N., Siniscalchi, L., Jones, J., Thomson, J., and Chen, G. (2012). Passaging and colony expansion of human pluripotent stem cells by enzyme-free dissociation in chemically defined culture conditions. Nat Protocols *7*, 2029-2040.

Carter, G., Bernstone, L., Sangani, D., Bee, J., Harder, T., and James, W. (2009). HIV entry in macrophages is dependent on intact lipid rafts. Virology *386*, 192-202.

Dunning, M., Smith, M., Ritchie, M., and Tavaré, S. (2007). beadarray: R classes and methods for Illumina bead-based data. Bioinformatics (Oxford, England) *23*, 2183-2184.

Durafourt, B., Moore, C., Blain, M., and Antel, J. (2013). Isolating, culturing, and polarizing primary human adult and fetal microglia. Methods in molecular biology (Clifton, NJ) *1041*, 199-211.

Etemad, S., Zamin, R.M., Ruitenberg, M., and Filgueira, L. (2012). A novel in vitro human microglia model: Characterization of human monocyte-derived microglia. Journal of neuroscience methods *209*, 79-89.

Falcon, S., and Gentleman, R. (2007). Using GOstats to test gene lists for GO term association. Bioinformatics (Oxford, England) *23*, 257-258.

Fernandes, H.J., Hartfield, E.M., Christian, H.C., Emmanoulidou, E., Zheng, Y., Booth, H., Bogetofte, H., Lang, C., Ryan, B.J., Sardi, S.P., *et al.* (2016). ER Stress and Autophagic Perturbations Lead to Elevated Extracellular alpha-Synuclein in GBA-N370S Parkinson's iPSC-Derived Dopamine Neurons. Stem cell reports *6*, 342-356.

Kapellos, T., Taylor, L., Lee, H., Cowley, S., James, W., Iqbal, A., and Greaves, D. (2016). A novel real time imaging platform to quantify macrophage phagocytosis. Biochemical pharmacology *116*, 107-119.

Morrison, M., Klein, C., Clemann, N., Collier, D.A., Hardy, J., Heisserer, B., Cader, M.Z., Graf, M., and Kaye, J. (2015). StemBANCC: Governing Access to Material and Data in a Large Stem Cell Research Consortium. Stem cell reviews *11*, 681-687.

Patro, R., Duggal, G., Love, M., Irizarry, R., and Kingsford, C. (2017). Salmon provides fast and bias-aware quantification of transcript expression. Nature methods.

Ritchie, M., Phipson, B., Wu, D., Hu, Y., Law, C., Shi, W., and Smyth, G. (2015). limma powers differential expression analyses for RNA-sequencing and microarray studies. Nucleic acids research *43*, gkv007-e047.

Sandor, C., Robertson, P., Lang, C., Heger, A., Booth, H., Vowles, J., Witty, L., Bowden, R., Hu, M., Cowley, S., *et al.* (2017). Transcriptomic profiling of purified patient-derived dopamine neurons identifies convergent perturbations and therapeutics for Parkinson's disease. Human molecular genetics.

Shi, Y., Kirwan, P., and Livesey, F.J. (2012). Directed differentiation of human pluripotent stem cells to cerebral cortex neurons and neural networks. Nature protocols *7*, 1836-1846.

van Wilgenburg, B., Browne, C., Vowles, J., and Cowley, S. (2013). Efficient, long term production of monocyte-derived macrophages from human pluripotent stem cells under partly-defined and fully-defined conditions. PloS one *8*, e71098.

Zhang, Y., Sloan, S., Clarke, L., Caneda, C., Plaza, C., Blumenthal, P., Vogel, H., Steinberg, G., Edwards, M., Li, G., *et al.* (2016). Purification and Characterization of Progenitor and Mature Human Astrocytes Reveals Transcriptional and Functional Differences with Mouse. Neuron *89*, 37-53.
