# Supplementary figures and images for: A Highly Efficient Human Pluripotent Stem Cell Microglia Model Displays a Neuronal-Co-culture-Specific Expression Profile and Inflammatory Response
Source: Stem Cell Reports. 2017 Jun 9;8(6):1727–42. doi: 10.1016/j.stemcr.2017.05.017 (PMC5470330; doi:10.1016/j.stemcr.2017.05.017)

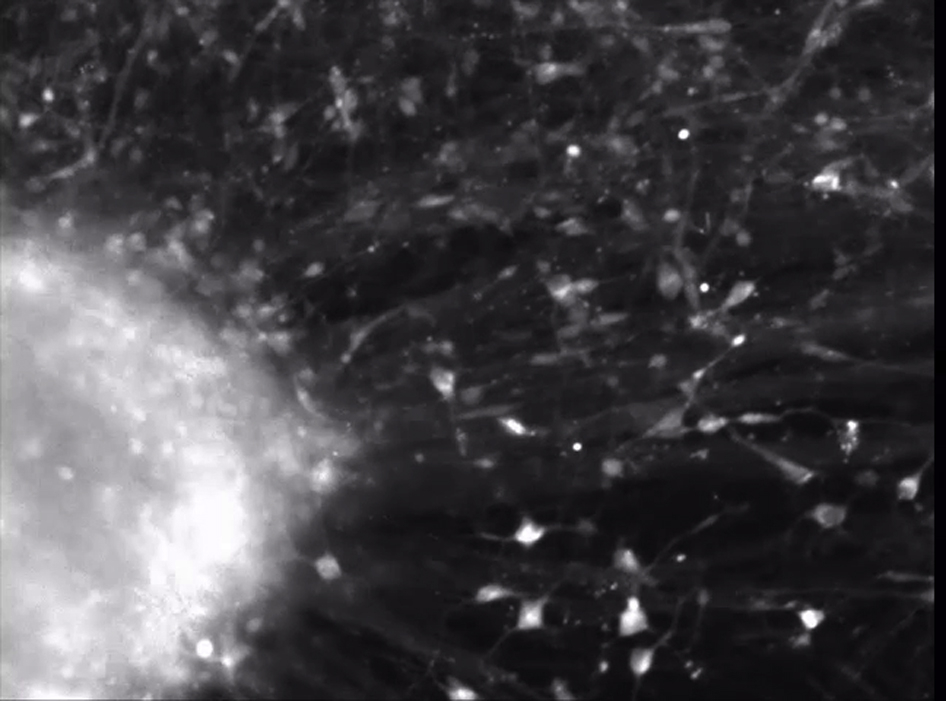

Supplement: Movie S1. Calcium Imaging in Neuronal Monoculture Followed by Images of Co-culture; Images Were Taken Every 3 Seconds for 2 Minutes, Related to Figure 1 [file mmc3.jpg]

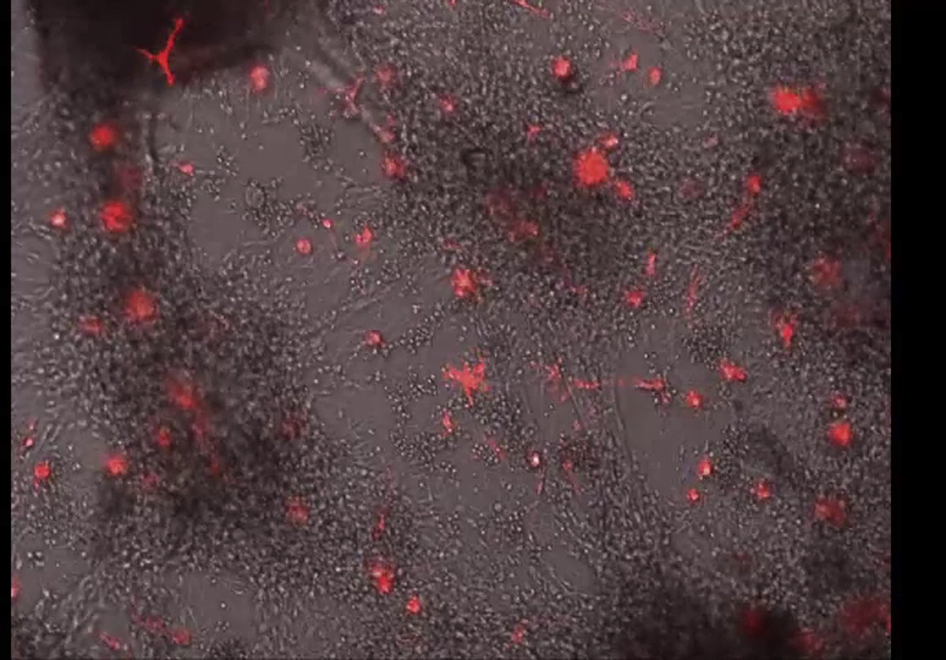

Supplement: Movie S2. RFP Co-culture Microglia Unstimulated, Red Channel/Phase Overlay; Images Were Taken Every 5 Minutes for 20 Hours, Related to Figure 5 [file mmc4.jpg]

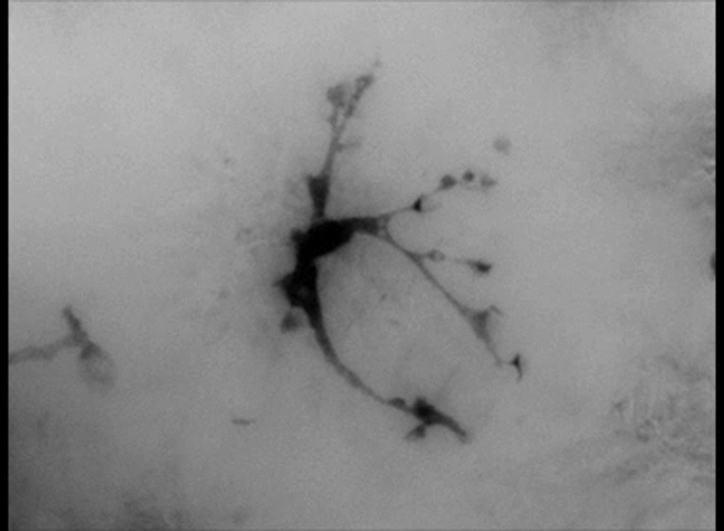

Supplement: Movie S3. RFP Co-culture Microglia Unstimulated, Red Channel Red Channel Inverted LUT; Images Were Taken Every 12 Seconds for 1 Hour, Related to Figure 5 [file mmc5.jpg]

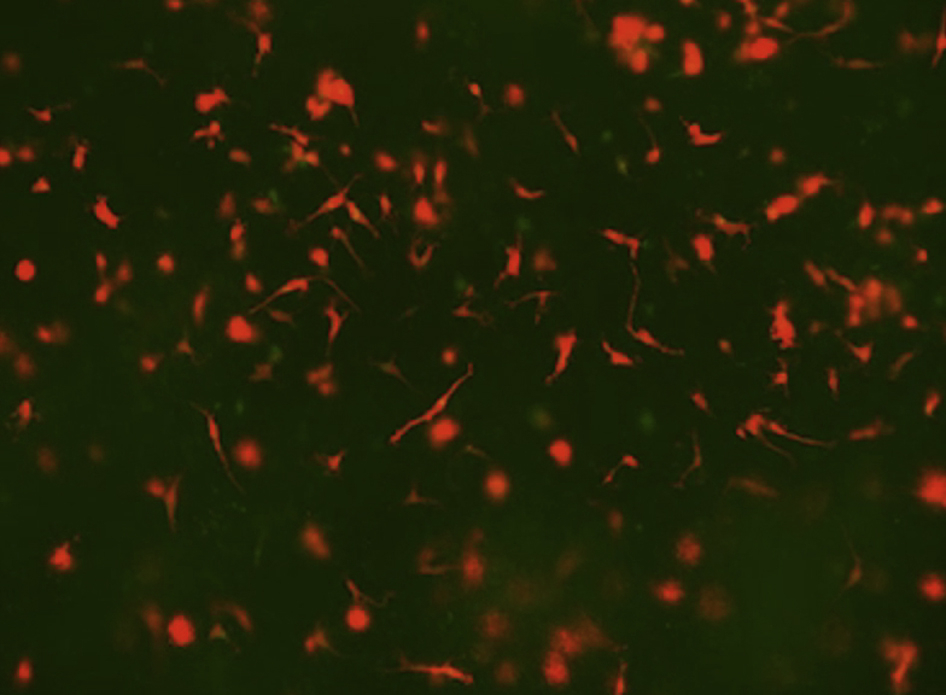

Supplement: Movie S4. RFP Co-Culture Microglia Fed pHrodo Green Zymosan, Followed by RFP pMac Fed pHrodo Green Zymosan; Images Were Taken Every 10 Minutes for 3 Hours, Related to Figure 5 [file mmc6.jpg]

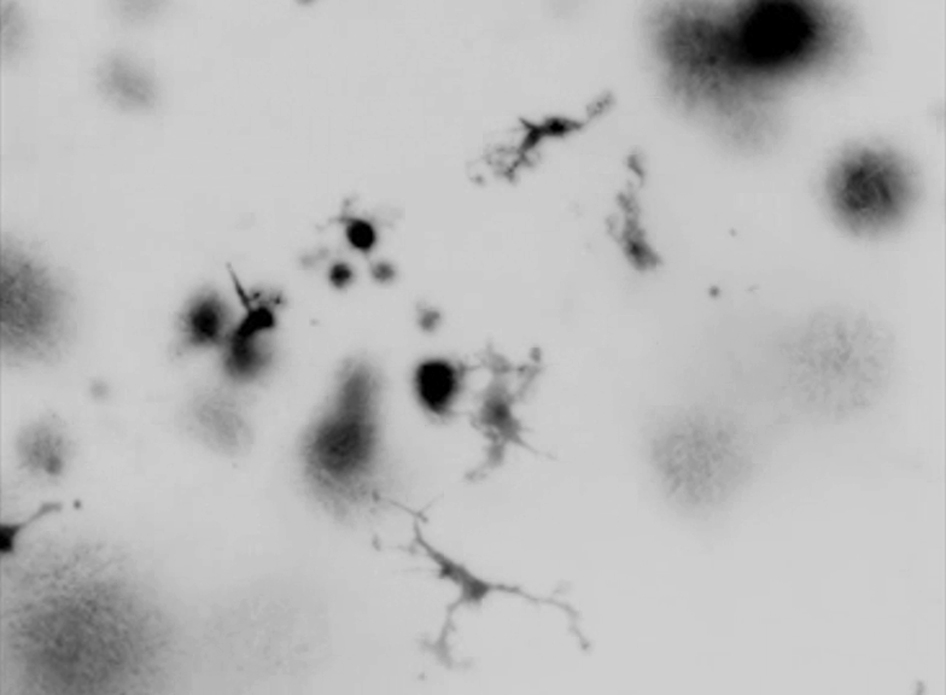

Supplement: Movie S5. RFP Co-culture Microglia Unstimulated Day 12, Followed by RFP Co-culture Microglia Stimulated with LPS, Red Channel Inverted LUT; Images Were Taken Every 5 Minutes for 17.5 Hours, Related to Figure 6 [file mmc7.jpg]
