# Supplementary material for: A Highly Efficient Human Pluripotent Stem Cell Microglia Model Displays a Neuronal-Co-culture-Specific Expression Profile and Inflammatory Response
Source: Stem Cell Reports. 2017 Jun 9;8(6):1727–42. doi: 10.1016/j.stemcr.2017.05.017 (PMC5470330; doi:10.1016/j.stemcr.2017.05.017)
Supplement: Document S2. Article plus Supplemental Information [file mmc8.pdf]

Stem Cell Reports
Article
OPEN ACCESS

A Highly Efficient Human Pluripotent Stem Cell Microglia Model Displays a Neuronal-Co-culture-Specific Expression Profile and Inflammatory Response

Walther Haenseler,[1,11] Stephen N. Sansom,[2,11] Julian Buchrieser,[1] Sarah E. Newey,[3] Craig S. Moore,[4] Francesca J. Nicholls,[5] Satyan Chintawar,[6] Christian Schnell,[7] Jack P. Antel,[8] Nicholas D. Allen,[7] M. Zameel Cader,[6] Richard Wade-Martins,[9,10] William S. James,[1] and Sally A. Cowley[1,10,*]
[1]Sir William Dunn School of Pathology, University of Oxford, South Parks Road, Oxford OX1 3RE, UK
[2]Kennedy Institute of Rheumatology, University of Oxford, Roosevelt Drive, Headington, Oxford OX3 7FY, UK
[3]Department of Pharmacology, University of Oxford, Oxford OX1 3QT, UK
[4]Division of BioMedical Sciences, Faculty of Medicine, Memorial University of Newfoundland, St. John's, NL A1B 3V6, Canada
[5]Department of Psychiatry, University of Oxford, Warneford Hospital, Oxford OX3 7JX, UK
[6]Weatherall Institute of Molecular Medicine, University of Oxford, Oxford OX3 9DS, UK
[7]School of Biosciences, College of Biomedical and Life Sciences, Cardiff University, Cardiff CF10 3AT, UK
[8]Montreal Neurological Institute, McGill University, Montreal, QC H3A 2B4, Canada
[9]Department of Physiology, Anatomy and Genetics
[10]Oxford Parkinson's Disease Centre
University of Oxford, South Parks Road, Oxford OX1 3QX, UK
[11]Co-first author
*Correspondence: sally.cowley@path.ox.ac.uk
http://dx.doi.org/10.1016/j.stemcr.2017.05.017

SUMMARY

Microglia are increasingly implicated in brain pathology, particularly neurodegenerative disease, with many genes implicated in Alzheimer's, Parkinson's, and motor neuron disease expressed in microglia. There is, therefore, a need for authentic, efficient in vitro models to study human microglial pathological mechanisms. Microglia originate from the yolk sac as *MYB*-independent macrophages, migrating into the developing brain to complete differentiation. Here, we recapitulate microglial ontogeny by highly efficient differentiation of embryonic *MYB*-independent iPSC-derived macrophages then co-culture them with iPSC-derived cortical neurons. Co-cultures retain neuronal maturity and functionality for many weeks. Co-culture microglia express key microglia-specific markers and neurodegenerative disease-relevant genes, develop highly dynamic ramifications, and are phagocytic. Upon activation they become more ameboid, releasing multiple microglia-relevant cytokines. Importantly, co-culture microglia downregulate pathogen-response pathways, upregulate homeostatic function pathways, and promote a more anti-inflammatory and pro-remodeling cytokine response than corresponding monocultures, demonstrating that co-cultures are preferable for modeling authentic microglial physiology.

INTRODUCTION

Microglia are brain-resident macrophages, with important homeostatic functions that provide a supportive environment to neurons. This includes pruning incompetent synapses during development, and clearance of dead cells, misfolded proteins, and other cellular debris (Ransohoff, 2016). However, they can become activated by inflammatory stimuli, producing a battery of cytokines, including the potentially damaging tumor necrosis factor α (TNFα). If not satisfactorily resolved, this response can lead to a chronically damaging cycle of activation and neuronal destruction. Numerous genes associated with Alzheimer's disease (AD), Parkinson's disease (PD), motor neuron disease/amyotrophic lateral sclerosis (MND/ALS), and frontotemporal dementia (FTD) are expressed in microglia, including *TREM2*, *CD33*, *LRRK2*, and *C9orf72* (O'Rourke et al., 2016; Russo et al., 2014; Villegas-Llerena et al., 2016), prompting a growing interest in microglia biology and their relevance to neurodegenerative disease.

Study of microglia has been largely restricted to non-human models (mostly mouse), since availability of fresh primary human microglia is very limited and they cannot be propagated. Moreover, microglia rapidly lose their unique identity when removed from the brain environment and cultured in monoculture in vitro (Butovsky et al., 2014). Transformed microglial-like cell lines are by definition highly proliferative and therefore not a good model for understanding a predominantly non-proliferating, differentiated cell type. There is therefore a need for practical, authentic human microglial cellular models. However, only recently has the ontogeny of microglia been established to inform appropriate modeling.

In mice, two waves of embryonic macrophages are produced in the yolk sac blood islands at embryonic day 7.5 (E7.5) and E8.25, and the first wave migrate into the developing brain and differentiate to microglia (Ginhoux et al., 2010; Gomez Perdiguero et al., 2015; Hoeffel et al., 2015; Palis et al., 1999). These yolk sac-derived macrophages are *Myb* independent but dependent on *PU.1* and

This is an open access article under the CC BY license (http://creativecommons.org/licenses/by/4.0/).

Irf8 (Kierdorf et al., 2013; Schulz et al., 2012). Hematopoietic stem cells (HSCs), in contrast, derive from the aorto-gonado-mesonephros region at day E10.5, populate the fetal liver and bone marrow, and give rise to adult blood cells from HSCs in bone marrow niches, which are dependent on *Myb* for their renewal. *Myb* independence, therefore, distinguishes yolk sac-derived macrophages from adult, definitive, blood monocyte-derived macrophages. Microglia in the developing brain proliferate locally at a low rate and are not normally replaced by other monocytes and macrophages from outside the brain, in contrast to most other tissue-resident macrophages (which also initially originate from yolk sac-derived macrophages, but are partially or fully replaced by fetal liver- or blood monocyte-derived macrophages [Bain et al., 2014; Calderon et al., 2015; Epelman et al., 2014; Guilliams et al., 2014; Hoeffel and Ginhoux, 2015; Tamoutounour et al., 2013]). In the brain, interleukin-34 (IL-34) is an alternative CSF1R ligand supporting microglia survival and differentiation (Greter et al., 2012), and microglia adopt an increasingly ramified morphology and continued maturation far beyond birth.

In humans there are few opportunities to investigate the ontogeny of microglia, but it is assumed that the processes are analogous to those in mice. Yolk sac-derived macrophages appear at E17 (Tavian and Peault, 2005), enter the brain from E31 onward (Rezaie et al., 2005; Monier et al., 2007), and mature together with neurons to fully functional ramified microglia (Figure 1A). Human cortical neurons show spontaneous electrical activity after microglia invasion, from gestation week 20 onwards (Moore et al., 2011).

We aimed to recapitulate the in vivo developmental pathway of microglia in vitro, using human induced pluripotent stem cells (iPSCs). These have the advantages of limitless self-renewal and normal karyotype, and can be directed to terminally differentiated cell types. They can be derived from patients (retaining the patient's genetic background) and are amenable to gene editing, enabling sophisticated interrogation of genes of interest. To recapitulate the development of yolk sac-derived macrophages, we use our previously established, straightforward, highly efficient, serum- and feeder-free protocol for deriving PSC macrophages (Karlsson et al., 2008; van Wilgenburg et al., 2013). We have recently directly demonstrated that these derive from *MYB*-independent, *RUNX1*- and *PU.1*-dependent precursors, characteristic of yolk sac-derived macrophages (Buchrieser et al., 2017; Vanhee et al., 2015). Here, we co-culture them with iPSC cortical neurons (Shi et al., 2012), in medium optimized for survival and functionality of both neurons and microglia. The resulting co-cultures are stable for many weeks, express relevant microglia markers (including key disease-related genes), upregulate pathways relating to homeostatic functions, and downregulate pathogen-response pathways. They are phagocytic, display highly dynamic ramifications, respond to activation by clustering and adoption of ameboid morphology, and produce cytokine profiles that are specific to co-culture versus monoculture.

RESULTS

iPSC-Derived Embryonic Macrophages Co-cultured with iPSC-Derived Neurons Recapitulate Microglial Development in the Embryo

To recapitulate the development of microglia in the embryo (Figure 1A), we used our previously established protocol (van Wilgenburg et al., 2013) to generate embryonic-like, *MYB*-independent macrophage precursors from iPSCs from four different donors (Table S1). Defined-size embryoid bodies (EBs) are formed using Aggrewells (STEMCELL Technologies), cultured with bone morphogenetic protein 4 (BMP4; to induce mesoderm), vascular endothelial growth factor (VEGF; endothelial precursors), and stem cell factor (SCF; hematopoietic precursors), then plated into large-format flasks with IL-3 and macrophage colony-stimulating factor (M-CSF) to promote myeloid differentiation. Most EBs adhere, put out surrounding adherent stromal cells, and develop cystic, yolk-sac-like structures. After 3–4 weeks, embryonic-like macrophage precursors emerge into the supernatant as a uniform population of large, round cells with obvious filopodia and ruffles. Originally termed "monocytes" (Karlsson et al., 2008; van Wilgenburg et al., 2013), we now understand their ontogeny to be *MYB*-independent primitive myeloid cells (Buchrieser et al., 2017), so they are more accurately termed macrophage precursors (pMacpre, Figure 1B). These can simply be harvested by collecting the supernatant without disrupting the EBs and replenishing flasks with fresh medium for many subsequent weekly harvests. The cumulative yield of pMacpre for the lines used in this study was 10- to 43-fold higher than the number of input iPSCs, consistent with yields previously reported for this protocol (van Wilgenburg et al., 2013), similar to a recently published hiPS-microglia protocol (typically 40-fold) (Abud et al., 2017), and 10-fold higher than two other recently published hiPS-microglia protocols (0.5- to 4-fold and 0.8- to 3-fold yields relative to input iPSCs, respectively) (Muffat et al., 2016; Pandya et al., 2017).

To mimic the subsequent seeding of embryonic macrophages into the developing brain, we co-cultured harvested pMacpre with iPSC-derived cortical neurons (Shi et al., 2012) (Figure 1C). We designed the co-culture medium to maintain microglia survival, which is dependent on signaling through the tyrosine kinase receptor CSF1, so we included the CSF1R ligand IL-34 (the alternative CSF1

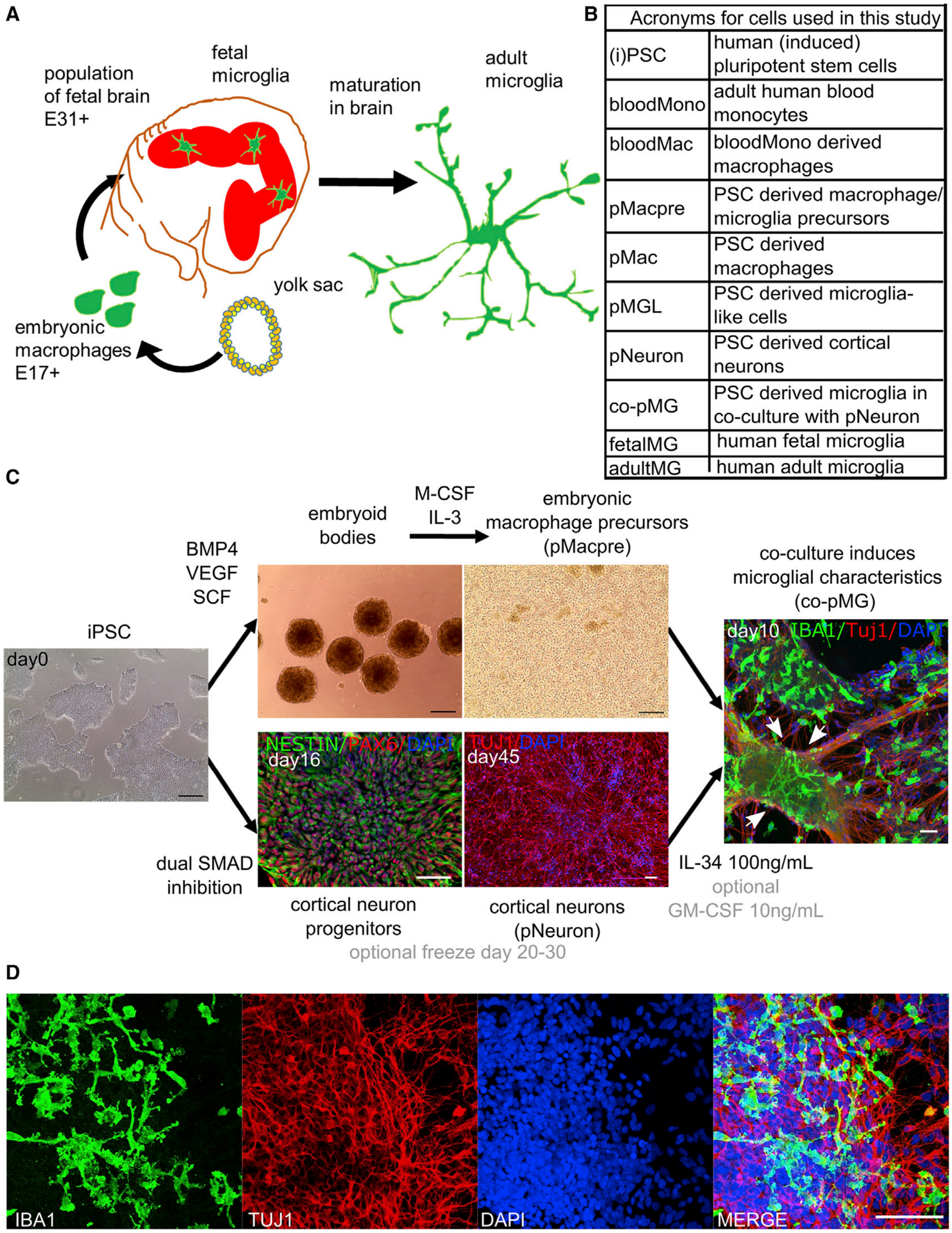

Acronyms for cells used in this study	
(i)PSC	human (induced) pluripotent stem cells
bloodMono	adult human blood monocytes
bloodMac	bloodMono derived macrophages
pMacpre	PSC derived macrophage/ microglia precursors
pMac	PSC derived macrophages
pMGL	PSC derived microglia-like cells
pNeuron	PSC derived cortical neurons
co-pMG	PSC derived microglia in co-culture with pNeuron
fetalMG	human fetal microglia
adultMG	human adult microglia

Figure 1. iPSC-Derived Microglia-Neuron Co-culture Recapitulates Microglial Development in the Embryo

(A) Human microglia originate from the yolk sac as primitive macrophages, migrating into the fetal brain before the formation of the blood-brain barrier, and completing their maturation in the brain environment.

(B) Acronyms used for the cell types in this study.

(C) Co-culture of iPSC embryonic macrophages and iPSC-cortical neurons with IL-34 (and, optionally, low level GM-CSF) recapitulates development of microglia in the brain. White arrows: highly ramified cells most evident in dense neuron clusters.

(D) Ramified microglia after 2 weeks of co-culture.

Black scale bars, 200 μm; white scale bars, 50 μm. See also Figures S1 and S2; Movie S1.

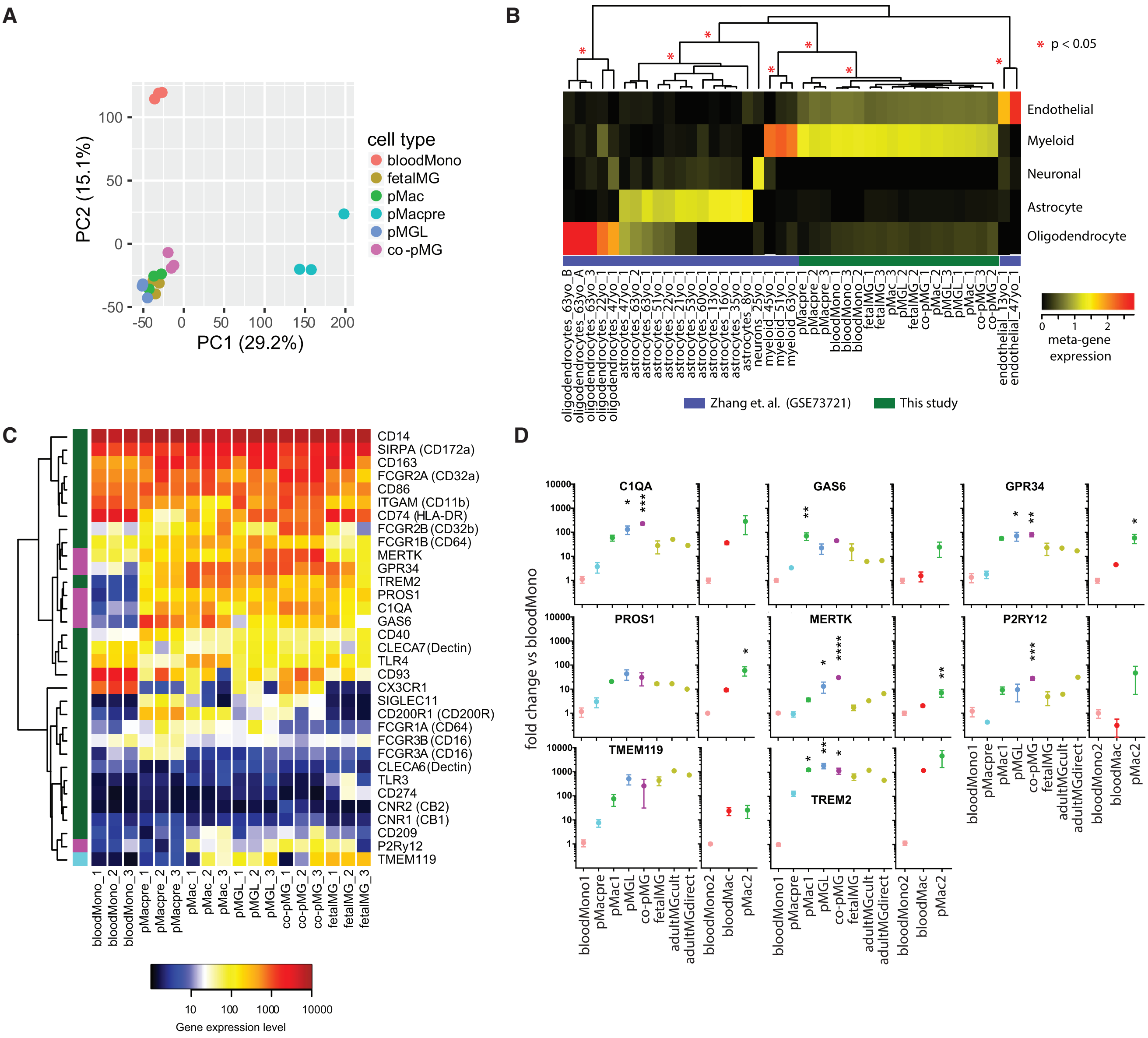

Figure 2. iPSC Co-culture Microglia Express Consensus Microglia Markers

Illumina HT12v4 transcriptome analysis of blood monocytes (bloodMono), iPSC-derived macrophage precursors (pMacpre), iPSC-derived macrophages (pMac), iPSC macrophages in microglia medium (pMGL), iPSC co-culture microglia isolated from co-culture (co-pMG), and freshly isolated human primary fetal microglia (fetalMG) (three genetic backgrounds each).

(A) Principal component (PC) analysis of samples based on protein coding gene expression. Numbers in parentheses indicate the percentage variance.

(B) Cell type analysis. Samples are hierarchically clustered by their expression of metagenes defined by non-negative matrix factorization from a previously published expression dataset for human cortex myeloid cells, cortical oligodendrocytes, astrocytes, neurons, and endothelial cells (Zhang et al., 2016). Red asterisks indicate significant clusters (pvclust, approximately unbiased).

(C) Expression of consensus human microglia/monocyte markers. Genes found by Melief et al. (2012) to be highest in microglia are highlighted in green on left-hand bar; six genes identified as being differentially expressed in microglia versus blood monocytes by Butovsky et al. (2014) are magenta, and TMEM119 (Bennett et al., 2016), is azure. Rows are hierarchically clustered.

(D) Expression of key microglia markers by qRT-PCR. Fold change was calculated using the ΔΔCT method, with 18S RNA as an endogenous control and normalization to bloodMono. Three genetic backgrounds for all conditions, as per transcriptome samples, with additional comparison with cultured adult human microglia (n = 1, with technical PCR triplicates) and with directly isolated/processed adult human

(legend continued on next page)

ligand produced in the brain, M-CSF being the main ligand in the periphery). We also sought compatibility with iPSC cortical neurons in culture, but aimed to reduce the presence of components in neuronal media that might compromise microglia function, so the neuronal supplement B27 was not included as it contains corticosterone, superoxide dismutase (SOD), and catalase. Finally, in pilot experiments, we tested the ability of different media and growth factors to induce ramified microglia-like morphology in our macrophages, following previous evidence that astrocyte-derived granulocyte M-CSF (GM-CSF), M-CSF, and transforming growth factor β (TGFβ) can each induce ramified morphology in microglia (Schilling et al., 2001), and that IL-34 and GM-CSF can ramify blood monocytes (Etemad et al., 2012; Ohgidani et al., 2014). Advanced DMEM/F12 with N2 and 100 ng/mL IL-34 satisfied the requirement for CSF1R engagement and neuronal compatibility, while IL-34 with (optional) low-dose GM-CSF (10 ng/mL) induced the most ramified morphology (Figures S1A–S1D), so this microglia medium was used for all subsequent experiments (Table S2). In microglia medium and in co-culture with iPSC cortical neurons (pNeurons), pMacpre adopted ramified microglial morphology with secondary branching within 2 weeks (Figures 1C and 1D). They are referred to hereafter as co-culture PSC microglia, or co-pMG. PSC macrophages are termed pMac, unless cultivated in microglia medium (as monocultures), when they are termed PSC microglia-like cells, pMGL.

Co-culture Is Compatible with iPSC Cortical Neuronal Maturation and Function

Co-culture could be extended for at least 42 days, during which time neurons maintained spontaneous electrical activity at least as well as neurons cultured alone (Figure S1E), and calcium flux was observable upon addition of potassium ions (Movie S1). Pre- and postsynaptic markers (Synaptophysin and PSD95) were observable in neuronal monocultures and in co-cultures (Figures S1F and S1G). Neuronal progenitors present in the cultures continued to proliferate, leading to an increase in the density of the cultures, whereas proliferation, as assessed by Ki-67 staining, was very low in co-pMG (similar to pMGL and pMac, Figures S1H–S1K). For this reason, most assays were conducted after 2 weeks of co-culture. Nonetheless, co-pMG persisted within the extended-duration cultures, maintaining expected density to at least day 39 (Figure S2). Co-pMG were dependent on CSF1R ligand delivery in the culture medium for persistence in co-culture, as withdrawal of IL-34 led to depletion of co-pMG (data not shown). Finally, co-culture neurons expressed both deep-layer (TBR1) and upper-layer (SatB2) cortical identity markers (Figure S2). Together, these observations indicate that co-culture conditions were compatible for co-pMG and had no detrimental effect on the maturity and functionality of the neurons.

Transcriptome Analysis Demonstrates a Microglial Signature in iPSC Co-culture Microglia

To assess to what extent the co-cultured cells resembled microglia, we isolated co-pMG from the neuronal culture using CD11b magnetic beads and compared their transcriptome with human fetal microglia (fetalMG), pMGL, pMac, pMacpre, and fresh adult blood-derived monocytes (bloodMono).

Based on their first two principal components, the samples separated into three distinct groups comprised of (1) bloodMono, (2) pMacpre, and (3) pMac, pMGL, co-pMG, and fetalMG (Figure 2A). The bloodMono and pMacpre samples showed an orthogonal separation from the macrophage and microglia samples that is in line with the different developmental origins of these cells. To investigate this possibility, we identified the set of genes significantly differentially expressed between these two populations (Figure S3 and Table S3). *FLT3*, a marker of definitive hematopoiesis, showed higher expression in the bloodMono samples (5.5-fold, adjusted $p < 2.4 \times 10^{-8}$), along with several *HLA* genes, in agreement with blood monocytes exposure to priming cytokines in the blood and their role in antigen presentation to T cells. Meanwhile *MAF*, a known marker of primitive hematopoiesis, showed higher expression in pMacpre (21.7-fold, adjusted $p < 7.8 \times 10^{-7}$). *APOE*, variants of which are major risk factors for AD, was among the most strongly differentially expressed genes, being very low in bloodMono and high in all other populations (Figure S3A).

Comparison with a previously published expression dataset for cells derived from human brain tissue (Zhang et al., 2016) showed that all the cell types in the current study cluster with human cortex myeloid cells and not with cortical oligodendrocytes, astrocytes, neurons, or endothelial cells (Figure 2B).

As a set, genes previously identified to be associated specifically with microglia but not with blood monocytes (Bennett et al., 2016; Butovsky et al., 2014; Melief et al., 2012) (Figure 2C) showed similar expression in co-pMG

microglia (n = 1, technical PCR triplicates). A second set of bloodMono were also differentiated to macrophages and assessed for these markers, alongside a second batch of pMac (three genetic backgrounds each). Mean ± SEM, one-way ANOVA, Dunnett's multiple comparisons test versus bloodMono. *p < 0.05, **p < 0.01, ***p < 0.001, ****p < 0.0001.
See Figures S3 and S4 for further transcriptomic analyses.

and fetalMG. Most notably, the six key microglia-specific genes identified by Butovsky et al. (2014), MERTK, GPR34, PROS1, C1QA, GAS6, and P2RY12, were all strongly expressed in co-pMG, whereas bloodMono poorly expressed most of these markers (highlighted in magenta, Figure 2C) These expression profiles were confirmed by qRT-PCR (Figure 2D), which also showed that co-pMG had comparable levels of expression of these microglial genes with cultured adult human microglia and with directly processed adult human microglia, and that while blood monocyte-derived macrophages also upregulated several of these genes, they mostly did not reach the same expression level. Notably, pMacpre, pMac, and pMGL also expressed high levels of most of these genes, along with many of those identified by Melief et al. (2012).

Next, we investigated the difference between the iPSC-derived macrophage and microglia populations. A targeted principal components analysis of these samples revealed a weak neural cell signature in the co-cultured pMG isolated with CD11b beads, but otherwise demonstrated the close similarity of these cells to fetal microglia (Figures S4A and S4B). We then sought to better understand the transcriptional differences between the differentiated PSC-derived samples pMac, pMGL, and co-pMG. We focused on genes with high and significantly variable expression using k-means clustering to identify five distinct signatures of gene expression (Figure S4C). Gene ontology analysis identified biological processes with significant enrichment in these gene sets, demonstrating that co-pMG downregulate genes in pathways associated with type I interferon responses (involved in antiviral responses), Toll-like receptor 1 (TLR1) and TLR2 signaling (bacterial and yeast recognition), and antigen presentation, relative to the monoculture populations. This implies that co-culture with neurons downregulates responses to external pathogens. Meanwhile, genes upregulated in co-pMG were enriched for biological processes including differentiation, chemotaxis/migration, regulation of cell-cell adhesion, and metal ion response (Hancock et al., 2014), all of which would be important for microglia to carry out their homeostatic surveillance and clearing functions.

Taken together, these results show the transcriptomic similarity of co-pMG with primary microglia, with expression of key microglial markers and genes in relevant homeostatic pathways, and downregulation of antimicrobial pathways in co-pMG. However, these results also highlight a previously unappreciated detail, which is that genes that have been previously identified as being specific to microglia versus blood monocytes are not necessarily exclusive to microglia, but a subset of them are more likely correlates of primitive macrophages, since they are also highly expressed in pMac.

Co-culture Microglia Express Genes Associated with Major Neurodegenerative Disease

Because numerous genes associated with AD, PD, MND/ALS, and FTD (through acquisition of mutations or SNP variants) have been found to be expressed in microglia, we examined the expression of these genes in our transcriptome dataset (Figure 3). *FERMT2*, *TREM2*, *APOE*, and *UCHL1* were expressed in fetalMG and co-pMG but not in bloodMono (although TREM2 was upregulated in bloodMono-derived macrophages, Figure 2D). Other key AD-related genes expressed in fetalMG and co-pMG (and bloodMono) included *APP*, *PICALM*, and *CD33*; PD-related genes included *PARK15*, *PINK1*, *SNCA*, and *DJ-1*; and MND-related genes included *C9orf72*, *TDP43*, and *SOD1*. Note that almost all of these genes were also expressed in pMacpre, pMac, and pMGL. Together, this shows that our co-culture system is a relevant model to study the effects of numerous genes associated with neurodegenerative disease, and that monoculture pMac or pMGL can be useful for answering specific disease gene-related questions where co-culture is impractical.

Co-culture Microglia Express Macrophage-/Microglia-Relevant Proteins

We next sought evidence for the functional protein products of key microglia genes. Flow cytometry with directly conjugated antibodies showed CD11b (integrin alpha M, a marker for mature myeloid cells and a subunit of the complement receptor, CR3, also known as Mac-1), CD14 (a component of the receptor for bacterial lipopolysaccharide [LPS]), and CD45 (a pan-leukocyte marker and tyrosine phosphatase which dephosphorylates several receptor tyrosine kinases), were expressed on all PSC-derived macrophage and microglia (Figures 4A and 4B). CD11c (integrin alpha X, part of the inactivated-C3b receptor 4, CR4), was well expressed in monoculture cells, but weakly expressed in co-pMG. HLA-DR (a major histocompatibility complex [MHC] class II antigen) was undetectable in any PSC-derived myeloid lineages, reflecting the unprimed culture conditions. The microglia-associated protein MERTK was expressed highly on all PSC-derived macrophage and microglia conditions, while the AD-associated protein CD33 was detected albeit at low levels. An unconjugated polyclonal antibody to TMEM119 gave modest staining of pMGL but background staining was evident in co-culture cells, and an unconjugated antibody to the purinergic receptor P2YR12 showed strong staining of pMGL although background staining was similarly evident in co-culture (Figure S5). These markers did not increase in a consistent way during the time course of the co-cultures (Figures S5C–S5E). IBA1 (a cytoplasmic calcium-binding protein associated with myeloid cells, particularly microglia) was readily detectable in co-pMG by immunocytochemistry

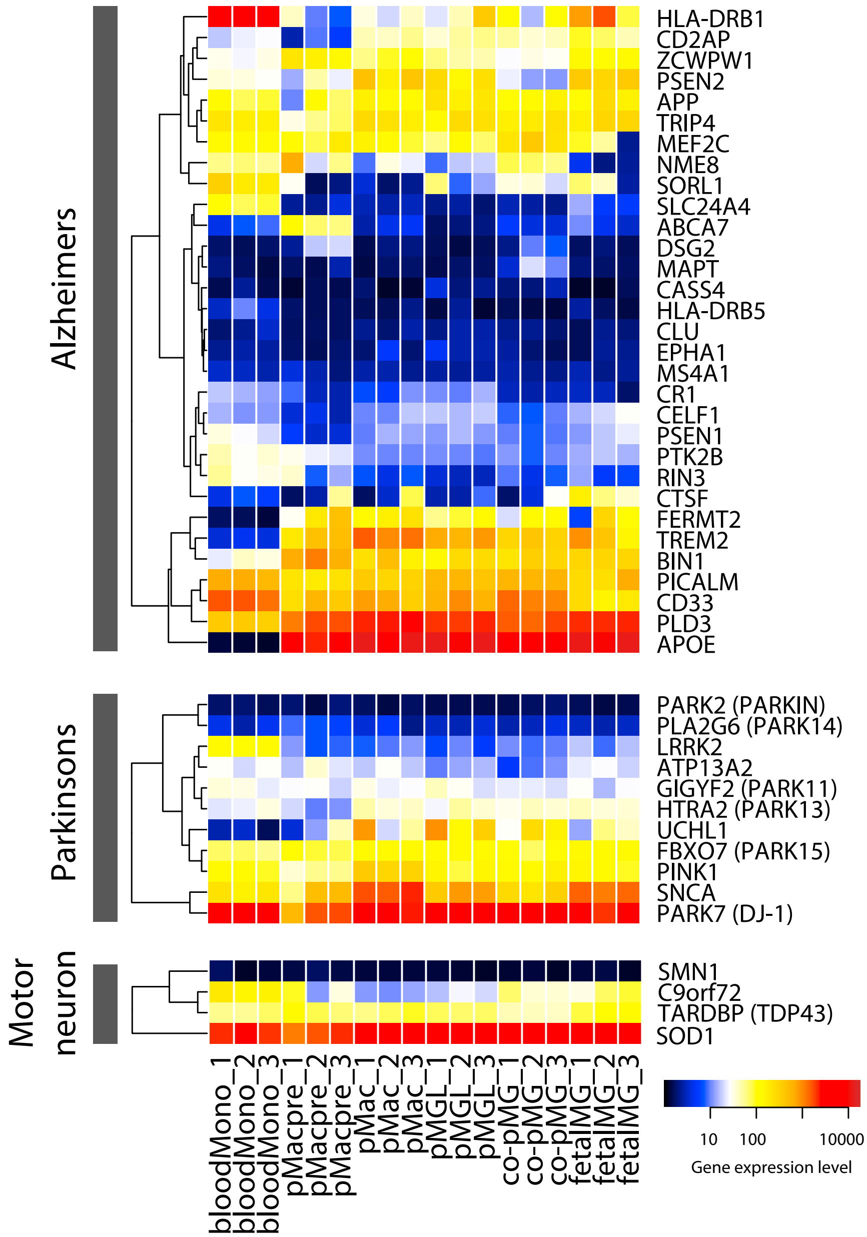

Figure 3. Expression of Genes Associated with Major Neurodegenerative Disorders

Samples and expression dataset as per Figure 2.

(Figures 1B and 1C). These results show that co-pMG and their primitive precursors express expected microglia and myeloid-associated proteins, although PSC macrophages and microglia have not been exposed to the cytokine milieu of the body and thus have low basal levels of proteins such as MHC antigens.

Co-culture Promotes Microglial Ramification and Motility

To identify and image microglia live in co-culture, we used co-pMG differentiated from an iPSC line containing multiple copies of an integrated lentivector containing the RFP gene under the control of the constitutively active EF-1α promoter. Co-pMG roughly "tile" within the neuronal culture, making direct contacts with the neurons (Figure 5A). Live imaging revealed a dynamically remodeled ramified morphology, every 5-min and even 12-s frame revealing changes to primary and secondary branching (Movies S2 and S3). Co-pMG moved constantly, most of them roughly maintaining their territories. Quantification of movement of co-pMG, pMGL, and pMac over 5 hr showed co-pMG moved a significantly greater accumulated distance (251 ± 21 μm; mean ± SEM; n = 6) than pMGL (119 ± 6 μm; n = 5) and pMac (53 ± 11 μm; n = 5),

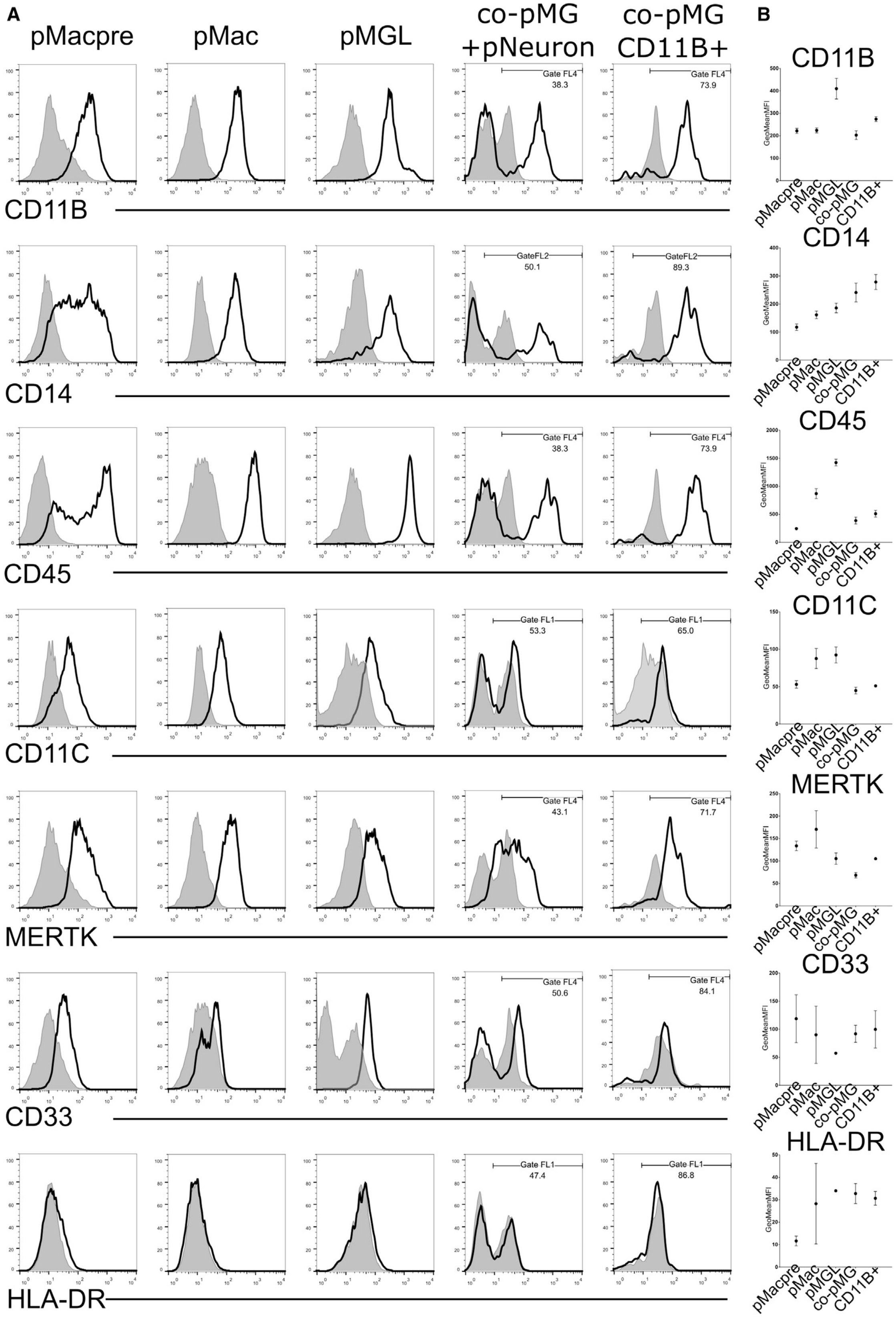

(legend on next page)

which hardly moved at all (Figures 5B–5F). Together, these results show that co-pMG display the morphology and dynamic behavior expected of microglia, continually sensing and responding to their neuronal environment. These features were a direct result of physical contact with neurons, as cells monocultured on tissue-culture plastic did not display such dynamic microglial characteristics.

Co-culture Microglia Are Phagocytically Competent

We have previously demonstrated that pMac are competent at phagocytosing particles, progressive acidification of the maturing phagosome being detectable using particles coupled to pH-sensitive fluorophores (Kapellos et al., 2016). pH-sensitive fluorescent zymosan particles added to co-cultures became visible inside co-pMG, within 1 hr, comparable with pMac (Movie S4), indicating the competent development of mature phagosomes in co-pMG.

iPSC Microglia Display Co-culture-Specific Inflammatory Responses

To explore the ability of co-pMG to respond physically to inflammatory signals, we stimulated co-cultures with LPS and imaged them over the next 20 hr. Unstimulated co-pMG retained roughly territorial surveillance behavior over the whole imaging period (Movie S5), suggesting no imaging-induced activation. In contrast, within 5 hr LPS-stimulated co-pMG migrated to form clusters, and some microglia had reduced ramifications and increased area-to-perimeter ratio, indicative of transition to activated, ameboid microglia (Figures 6A–6C and Movie S5). Clustering was measured as distance to nearest neighbor, showing a leftward shift (i.e., smaller distance) in curves for LPS-treated cultures, indicative of the clustering clearly observable by eye (Figure 6D). Blinded morphology scoring showed a significant increase in the proportion of cells with activated morphology in LPS-treated cultures in six analyzed videos (time point mean ± SEM: 0 hr, 4.1 ± 0.6; 10 hr, 7.6 ± 1.1; 20 hr, 8.1 ± 0.8; Figure 6E).

To examine the cytokine responses of co-pMG, we compared co-pMG and pMac using Proteome Profiler for 102 cytokines with and without maximal activation (LPS/interferon-γ [IFNγ], Table S2). Differentially expressed cytokines were then selected for a more detailed investigation using Luminex multiplex array, with supernatants from pMac, pMGL, co-pMG, and pNeuron, with or without LPS/IFNγ stimulation (Figure 7). There was broad correspondence across Proteome Profiler and Luminex platforms. Unstimulated neurons secreted only macrophage migration inhibitory factor (MIF) and VEGF-A, and when stimulated secreted IL-6, IL-8, and a subset of chemokines. pMac secreted very few cytokines constitutively (macrophage inflammatory protein 1α [MIP1α] and MIPβ, CXCL1 and CXCL10, IL-8, and MIF), but secreted the entire panel of 22 cytokines upon stimulation, in concordance with our previous publication (which also includes comparison with blood monocyte-derived macrophages [Jiang et al., 2012]). pMGL had a higher baseline number of cytokines secreted, and upregulated most cytokines upon stimulation (except IL-23A). GM-CSF did not account for this difference, as its absence did not significantly change the cytokine profile of pMGL (Figure S6).

Co-pMG displayed an overall dampened secretion of chemokines and cytokines versus pMGL, both constitutive and induced. Interestingly, SerpinE1 (a serine protease inhibitor that inhibits fibrinolysis) and VEGF-A (stimulates angiogenesis) were significantly higher in activated co-culture versus monoculture, suggesting that tissue remodeling factors are specifically induced in co-culture. Meanwhile, IL-10, which is anti-inflammatory, was significantly increased in activated co-culture versus monocultures. Together, these results indicate that co-culture induces specific anti-inflammatory and pro-remodeling responses not seen in corresponding monocultures.

DISCUSSION

We have established a highly efficient in vitro human iPSC-derived microglia-neuron co-culture model, which recapitulates the ontogenetic development of microglia in vivo. Co-pMG can be maintained in co-culture, retaining neuronal maturity and functionality for many weeks. They express key human microglia-specific markers and neurodegenerative disease-relevant genes, upregulate homeostatic pathways, downregulate pathogen-response pathways, and exhibit a transcriptional profile similar to that of fetal

Figure 4. iPSC Co-culture Microglia Express Relevant Protein Markers

(A) Flow cytometry of cells differentiated from one line (SFC856-03-04, black line is surface marker, filled gray area is isotype control). pMacPre were differentiated for 14 days to pMac, pMGL, or co-cultured with neurons to obtain co-pMG, which were either stained in single-cell suspension of the co-culture or isolated with CD11b beads before staining.

(B) Expression of surface markers (three genetic backgrounds, lines SBAD3-01, SFC840-03-03, SFC856-03-04) in pMacpre, pMac, pMGL, and co-cultures at 2, 7, 10, and 14 days. To remove obviously non-myeloid cells from MFI analysis, we set a gate to FL1, FL2, and FL4 for all cytometry data. Error bars denote SEM.

See Figure S7 for FSC/SSC gating and additional cytometry data.

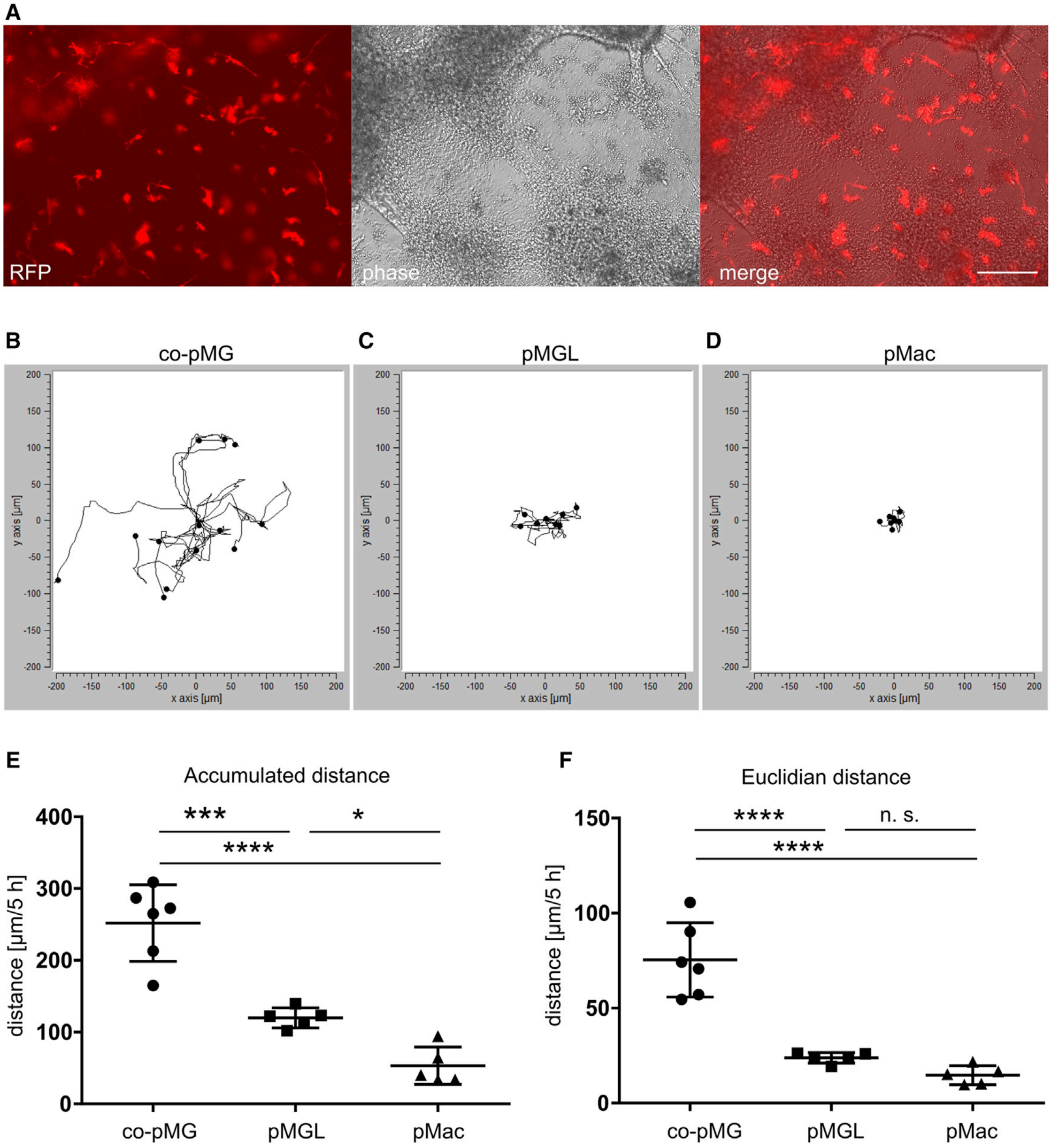

Figure 5. Co-culture with Neurons Promotes iPSC-Microglial Motility

Macrophages and co-culture microglia were imaged every 5 min for 5 hr (two videos each of three cultures i.e., six replicates per condition).

(A) co-pMG expressing RFP to enable identification in co-culture. Scale bar, 200 µm.

(B–D) Tracks of co-culture microglia (co-pMG; B), compared with cells on tissue-culture plastic: pMGL (C) and pMac (D).

|(E) Accumulated distance.

(F) Euclidian distance (distance in a straight line, start to end).

Error bars represent SD. Statistical analysis by Tukey's multiple comparisons test. *$p < 0.05$, ***$p < 0.001$, ****$p < 0.0001$; n.s., not significant. See also Movies S2, S3, andS4.

microglia. Co-pMG are phagocytic, adopt a highly dynamic ramified microglia-like morphology, upon activation display an activated morphology, and release a battery of microglia-relevant cytokines, with co-culture promoting a more anti-inflammatory and pro-remodeling cytokine response than corresponding monocultures. This shows the relevance of using co-culture to examine direct and paracrine microglia-neuronal interactions.

Since primary human microglia can only be obtained from fresh brain material, previous efforts have been made by others to develop methods for deriving microglia in vitro. Etemad and colleagues used human blood monocytes as the starting point (Etemad et al., 2012; Ohgidani et al., 2014), but it is now known that this pathway poorly recapitulates microglia ontogenesis and that blood is not a limitless source of cells, unlike PSCs. Beutner et al. (2013) described a method for deriving microglia from mouse embryonic stem cells, and, using the same methodology, Almeida et al. (2012) derived microglia from human iPSCs. However, this method directs the EBs through a neuronal differentiation pathway and thus does not replicate yolk sac myelopoiesis. Moreover, these cells do not express a convincing microglia signature (Butovsky et al., 2014; Melief et al., 2012). Schwartz et al. (2015) recently successfully seeded iPSC macrophages into 3D iPSC-neuronal structures for toxicity testing, but did not characterize the resulting cells extensively.

In 2008, we derived human PSC macrophages using a very simple methodology without OP9 feeders (Karlsson et al., 2008), which we subsequently adapted to a fully defined, robust serum-free protocol (van Wilgenburg et al., 2013), now widely used by others (Aflaki et al., 2014; Alasoo et al., 2015; Gupta et al., 2016). Our own work (Buchrieser et al., 2017) and that of others (Hoeffel et al., 2015) indicates that this protocol produces *MYB*-independent myeloid cells, recapitulating an embryonic ontogeny, and lineage-tracking studies in mouse have demonstrated that microglia derive from primitive, yolk sac macrophages that migrate into the developing brain (Ginhoux et al., 2010, 2013). Together, this provides a rationale for deriving PSC microglia, using PSC macrophages as a starting point and then skewing them toward a microglial phenotype. During the drafting of this manuscript, three protocols along these general principles have been published (Abud et al., 2017; Muffat et al., 2016; Pandya et al., 2017). Where our analyses overlap, there is broad consensus. However, only our protocol has direct evidence by gene knockout for producing *MYB*-independent primitive macrophages (Buchrieser et al., 2017). It is robust and efficient: time frame 1 month, versus 2 months for Muffat et al. (2016); yield 10–43 per starting iPSC for our protocol, versus 0.5–4 for Muffat et al. (2016) and Pandya et al. (2017); manipulation consisting of once-weekly feeding of differentiation cultures in flasks, simple supernatant harvest of pure precursors, and multiple harvests possible, versus sequential trituration/replating steps for Muffat et al. (2016), fluorescence-activated cell (FAC) sorting/plating on astrocytes/second FAC sorting for Pandya et al. (2017), and low oxygen concentrations/several replatings/five different cytokine cocktails/FAC sorting progenitors for Abud et al. (2017). Our microglia medium avoids B27, which contains cortisone, SOD, and catalase (likely to compromise microglia function). Muffat et al. (2016) characterized their iPSC microglia in monoculture, briefly assessing co-culture, and Pandya et al. (2017) characterized their iPSC microglia in monoculture following isolation from astrocytes. We have extensively characterized iPSC microglia in co-culture with iPSC cortical neurons, where cells have highly ramified, dynamic characteristics, and compared directly to the intermediate and parallel monoculture stages of differentiation. This reveals that while key microglia genes are expressed in co-pMG, they are often also expressed in pMacpre, pMac, and pMGL, indicating that such genes may be features of primitive macrophages rather than being microglia specific, and that comparison only with blood monocytes/macrophages does not give a complete interpretation. Finally, we show that co-culture induces a unique cytokine profile which is not the sum of monocultures.

There is a fast-growing interest in microglia, as they are increasingly implicated in neurodegenerative disease, neurodevelopmental disorders, and in neuropathic pain. Our iPSC microglia transcribe key genes involved in AD, PD, and MND. Several other disease-associated genes, including *LRRK2*, would be expected to be upregulated upon microglial stimulation (reviewed in Lee et al., 2017). Many of these genes are likely involved in phagocytosis and processing of misfolded proteins and of dying neurons (common features of these diseases), and in generating inappropriate chronic cytokine responses that exacerbate neuronal damage, creating a destructive cycle. Human iPSC microglia models enable study of these gene products at their correct gene dosage, in an authentic human in vitro system. Some of these functions and disease-relevant genes can be studied in the monoculture conditions detailed here, but others, involving crosstalk between microglia and neurons, such as paired receptor engagement, paracrine signaling, damage responses, synaptic surveillance, and pruning, will be better studied using the co-culture model we have described. The system is also amenable to scaling for the development of drug-screening assays to identify compounds that can improve microglial homeostatic clearance functions and dampen chronically activated microglia.

EXPERIMENTAL PROCEDURES

Consent for Use of Human Material

All human material (iPSCs, adult blood, fetal and adult microglia) was obtained with informed consent and with the approval of the relevant institutions (see Supplemental Information for full details).

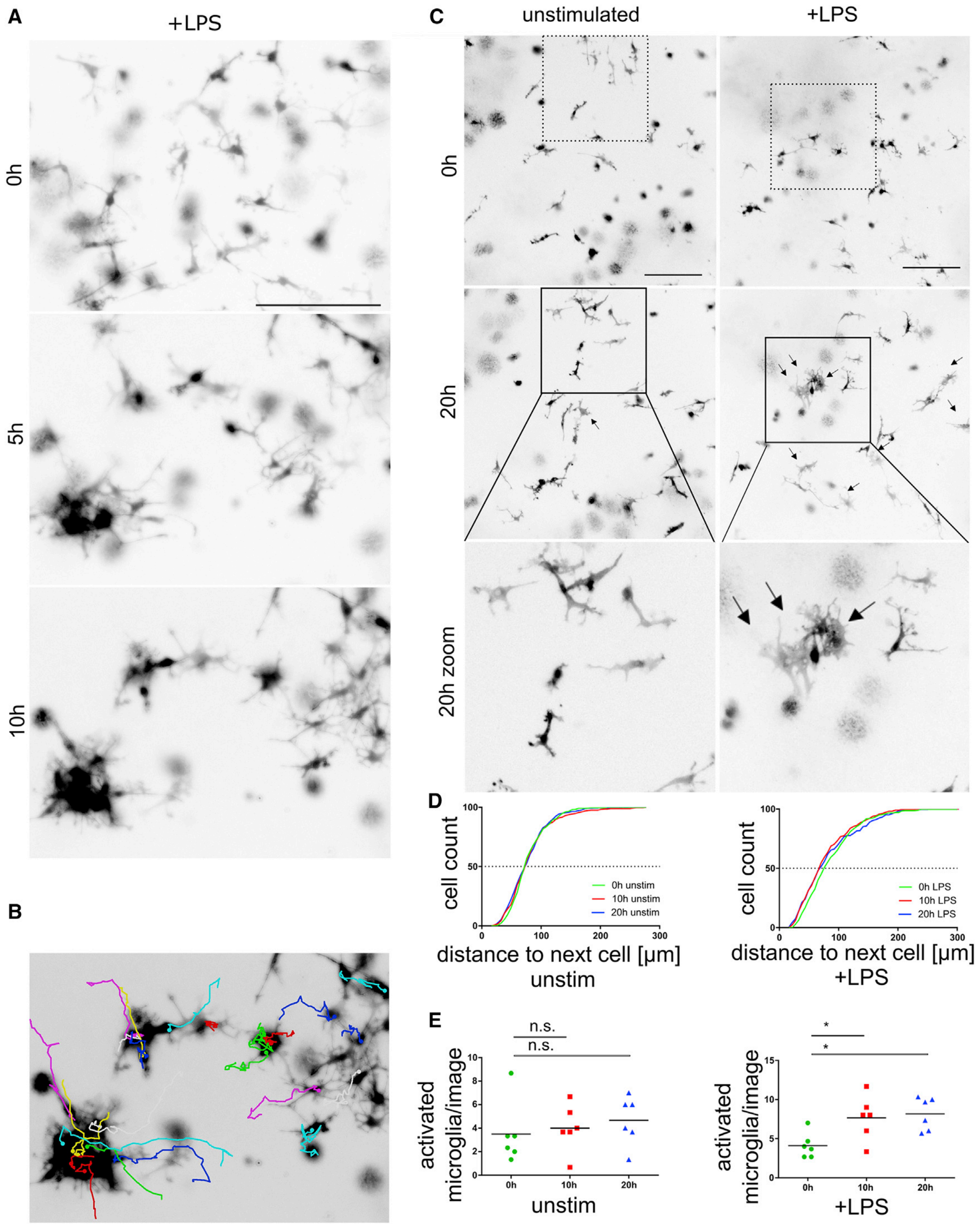

Figure 6. LPS Induces Inflammatory Morphology and Clustering in Co-culture Microglia

(A–D) Microglial morphology displayed as inverted LUT black and white images of RFP-iPSC microglia in co-culture. (A) Images (co-culture day 12) every 5 min for 10 hr. Representative images of LPS-stimulated co-pMG are shown at 0, 5, and 10 hr. (B) co-pMG clustering on LPS stimulation shown by cell tracking. (C–E) Quantitative analysis (two videos each of three cultures, i.e., six replicates per condition). Images were taken on co-culture on day 14 every 5 min for 20 hr. (C) Representative images of 0-hr and 20-hr time points; Bottom panel: area in the black square magnified to show microglial morphology: unstimulated co-pMG show no obvious changes in morphology during imaging period, but on LPS stimulation some microglia cluster, adopting more ameboid morphology with shorter processes and higher area-to-perimeter ratio, representative of a pro-inflammatory phenotype (black arrows). (D) Distance between microglia clustering upon

(legend continued on next page)

Macrophage Differentiation

See Supplemental Experimental Procedures for details of cells and assays used. iPSCs were differentiated to macrophages as previously described (van Wilgenburg et al., 2013). In short, 3 × 10^6 iPSCs were seeded into an Aggrewell 800 well (STEMCELL Technologies) to form EBs, in mTeSR1 and fed daily with medium plus 50 ng/mL BMP4 (Peprotech), 50 ng/mL VEGF (Peprotech), and 20 ng/mL SCF (Miltenyi Biotec). Four-day EBs were then differentiated in either 6-well plates (15 EBs/well), T75 (75 EBs), or T175 flasks (150 EBs) in X-VIVO15 (Lonza), supplemented with 100 ng/mL M-CSF (Invitrogen), 25 ng/mL IL-3 (R&D), 2 mM Glutamax (Invitrogen), 100 U/mL penicillin and 100 μg/mL streptomycin (Invitrogen), and 0.055 mM β-mercaptoethanol (Invitrogen), with fresh medium added weekly. pMacpre emerging into the supernatant after approximately 1 month were collected weekly and differentiation cultures replenished with fresh medium. Harvested cells were strained (40 μm, Corning) and used: either directly as pMacpre; or plated onto tissue-culture treated plastic or glass coverslips at 100,000 per cm^2 and differentiated for 7 days or more to pMac in X-VIVO15 with 100 ng/mL M-CSF, 2 mM Glutamax, 100 U/mL penicillin, and 100 μg/mL streptomycin; or co-cultured with iPSC-derived neurons.

Neuronal Differentiation

iPSCs were differentiated to cortical neuron progenitors (NPCs) (Shi et al., 2012) with the following modifications: feeder-free iPSCs were plated onto Matrigel-coated 6-well plates, with neural induction for 12 days using dual SMAD inhibition; after replating the neuroepithelial sheet at day 12 using dispase, 20 ng/mL fibroblast growth factor 2 (FGF2) was added to neural maintenance medium (NMM) on days 13–17, newly formed rosettes were dispased on days 17–18, and stocks of NPC were frozen at days 25–30.

Microglia Medium

In pilot experiments, macrophages were differentiated in three different basal media (XVIVO15, RPMI, or Advanced DMEM/F12 + N2 supplement) supplemented with 2 mM Glutamax, 100 U/mL penicillin and 100 μg/mL streptomycin, and 0.055 μM β-mercaptoethanol, with combinations of 100 ng/mL M-CSF (Invitrogen), 100 ng/mL IL-34 (Peprotech or Biolegend), and 10 ng/mL GM-CSF (Invitrogen). ADMEM/F12 + N2 supplement +100 ng/mL IL-34 + 10 ng/mL GM-CSF microglia medium was used for all further experiments (for details see Table S2).

Microglia-Neuron Co-culture

NPCs were thawed, centrifuged (200 × *g*, 10-fold volume NMM), and plated onto Matrigel in NMM supplemented with 10 μmol/L Y-27632 and 20 ng/mL FGF2. Medium was replaced the next day and every other day thereafter with NMM. After 7 days they were dissociated to single cells with StemPro Accutase (STEMCELL), added to their final Matrigel-coated format (Corning 96-well or 6-well plate, or Ibidi 8-well slide) at 100,000 cells/cm^2 and cultured for 14 days in NMM. pMacpre were resuspended in microglia medium, added at 100,000 cells/cm^2 to neurons, and co-cultured for a minimum 14 days before assaying.

Statistics

GraphPad Prism was used for statistical analysis. One-way ANOVA and Dunnett's multiple comparisons, Tukey's multiple comparisons, or paired two-tailed t test (for single comparisons) were used as indicated. Values are indicated in figures as *$p < 0.05$, **$p < 0.01$, ***$p < 0.001$, ****$p < 0.0001$, and n.s. (not significant). Numbers in parentheses are (mean ± SEM, n).

ACCESSION NUMBERS

The accession number for the Illumina HT12v4 expression array datasets reported in this paper is GEO: GSE89795.

SUPPLEMENTAL INFORMATION

Supplemental Information includes Supplemental Experimental Procedures, six figures, three tables, and five movies and can be found with this article online at http://dx.doi.org/10.1016/j.stemcr.2017.05.017.

AUTHOR CONTRIBUTIONS

Conceptualization, S.A.C., W.S.J., and W.H.; Methodology, S.E.N., S.C., W.H., S.A.C., and S.N.S.; Investigation, W.H.; Formal Analysis, W.H. and S.N.S.; Writing, W.H. and S.A.C.; Writing – Original Draft, W.H., S.N.S., and S.A.C.; Writing – Review & Editing, S.A.C., R.W.-M., M.Z.C., and W.S.J.; Funding Acquisition, S.A.C., R.W.-M., M.Z.C., W.H., and W.S.J.; Resources, N.D.A., S.E.N., C.S., F.J.N., C.S.M., J.P.A., S.C., S.N.S., and J.B.; Supervision, S.A.C. and W.S.J.

ACKNOWLEDGMENTS

Financial support: The Wellcome Trust WTISSF121302 and the Oxford Martin School LC0910-004 (James Martin Stem Cell Facility Oxford, W.H., S.A.C.); the MRC Dementias Platform UK Stem Cell Network Capital Equipment MC_EX_MR/N50192X/1, Partnership MR/N013255/1 (W.H., S.A.C., N.A., R.W.-M.) and Momentum MC_PC_16034 (W.H., S.A.C., M.Z.C.) Awards; the Swiss National Foundation Early Postdoc Mobility, 148607, and ARUK Oxford pilot grant (W.H.); the Kennedy Institute of Rheumatology Trust (S.N.S.); Royal Society Dorothy Hodgkin Fellowship (S.E.N.); Medical Research Council, Heatley Merck Sharpe and Dohme studentship (J.B.); seventh Framework Program, RepairHD (C.S.). The work was supported by the Innovative Medicines Initiative Joint Undertaking under grant agreement number 115439, resources of which are composed of financial contribution from the European Union's Seventh Framework Program (FP7/2007e2013) and EFPIA companies' in kind contribution. We thank

stimulation is evidenced by a leftward shift of the plot after 10 hr and 20 hr. (E) Micrographs were scored blind by three independent assessors for number of microglia with activated morphology. Statistical analysis by Dunnett's multiple comparison test. n.s., not significant; *$p < 0.05$.
Scale bars, 200 μm. See also Movie S5.

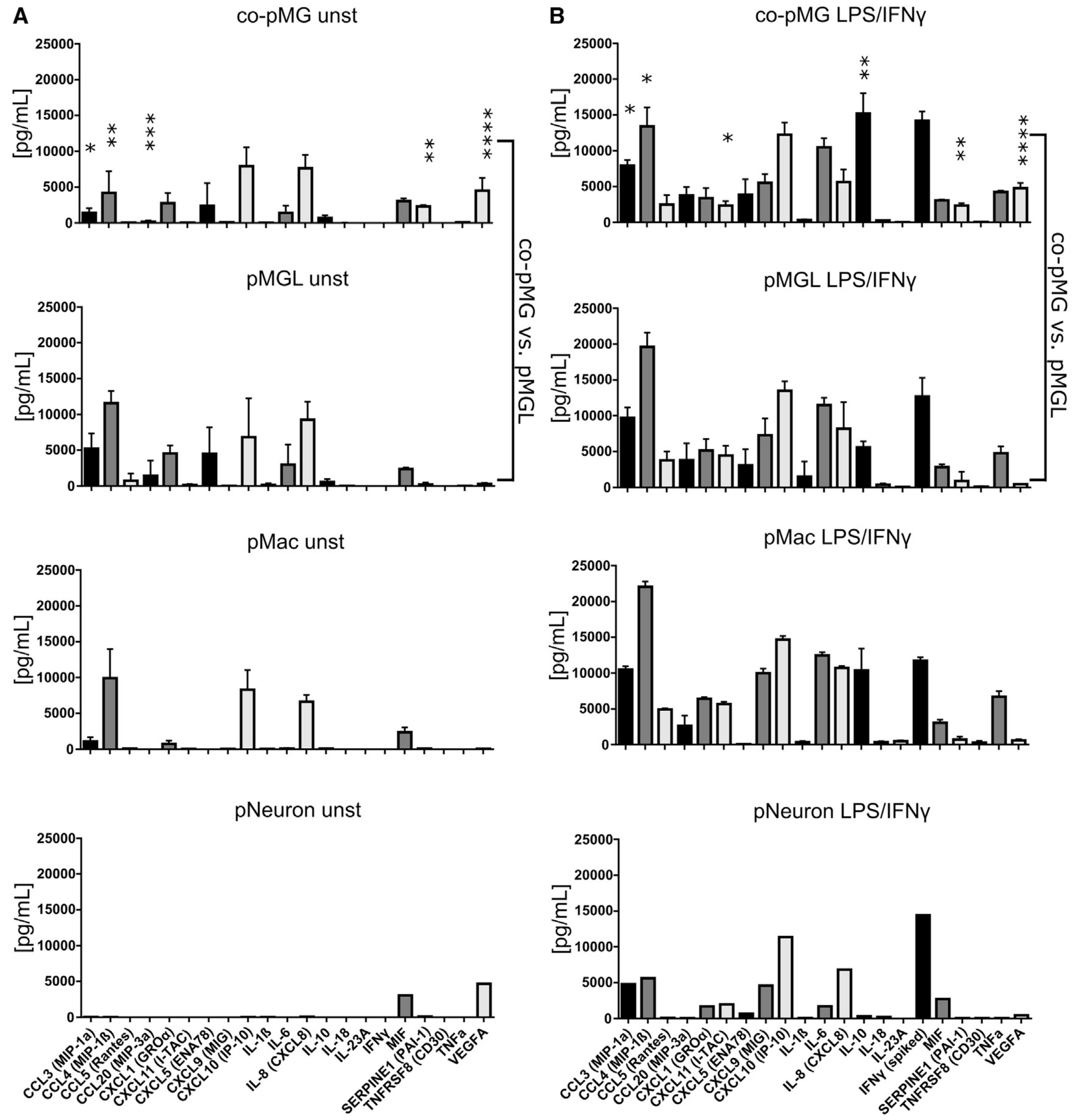

Figure 7. Cytokine Profiles of Co-culture versus Monocultures

Eighteen-hour supernatants from cells stimulated with or without LPS/IFNγ were assayed with a Luminex multiplex assay. DMEM/F12/N2-based microglia medium was used for monoculture (pMGL), co-culture (co-pMG), and neuron-only culture (pNeuron); standard XVIVO15-based macrophage medium was used for pMac. Medium alone contained negligible levels of all cytokines tested. Supernatants from lines: SBAD3-01 neurons ± SFC180-01-01, SFC840-03-03, and SFC856-03-04 macrophages/microglia.

(A) Unstimulated cells.

(B) LPS/IFNγ-stimulated cells.

Mean ± SD, three genetic backgrounds. Asterisks indicate significant difference between co-pMG and pMGL by two-tailed paired t test. $*p < 0.05$, $**p < 0.01$, $***p < 0.001$, $****p < 0.0001$. See Table S2 for further factors tested with pMac and co-pMG, and Figure S6 for effect of additional media and growth factor combinations on cytokine secretion.

the High-Throughput Genomics Group at the Wellcome Trust Center for Human Genetics, Oxford (Funded by Wellcome Trust grant reference 090532/Z/09/Z and MRC Hub grant G0900747 91070) for the generation of Illumina genotyping and transcriptome data. We would also like to thank the National Phenotypic Screening Center for instrument support. Samples and associated clinical data were supplied by the Oxford Parkinson's Disease Center (OPDC) study, funded by the Monument Trust Discovery Award from Parkinson's UK, a charity registered in England and Wales (2581970) and in Scotland (SC037554), with the support

of the National Institute for Health Research (NIHR) Oxford Biomedical Research Center based at Oxford University Hospitals NHS Trust and University of Oxford, and the NIHR Comprehensive Local Research Network.

Received: November 7, 2016
Revised: May 15, 2017
Accepted: May 15, 2017
Published: June 6, 2017

REFERENCES

Abud, E., Ramirez, R., Martinez, E., Healy, L., Nguyen, C., Newman, S., Yeromin, A., Scarfone, V., Marsh, S., Fimbres, C., et al. (2017). iPSC-derived human microglia-like cells to study neurological diseases. Neuron *94*, 278–293.e9.

Aflaki, E., Stubblefield, B., Maniwang, E., Lopez, G., Moaven, N., Goldin, E., Marugan, J., Patnaik, S., Dutra, A., Southall, N., et al. (2014). Macrophage models of Gaucher disease for evaluating disease pathogenesis and candidate drugs. Sci. Transl. Med. *6*, 240ra73.

Alasoo, K., Martinez, F.O., Hale, C., Gordon, S., Powrie, F., Dougan, G., Mukhopadhyay, S., and Gaffney, D.J. (2015). Transcriptional profiling of macrophages derived from monocytes and iPS cells identifies a conserved response to LPS and novel alternative transcription. Sci. Rep. *5*, 12524.

Almeida, S., Zhang, Z., Coppola, G., Mao, W., Futai, K., Karydas, A., Geschwind, M.D., Tartaglia, M.C., Gao, F., Gianni, D., et al. (2012). Induced pluripotent stem cell models of progranulin-deficient frontotemporal dementia uncover specific reversible neuronal defects. Cell Rep. *2*, 789–798.

Bain, C.C., Bravo-Blas, A., Scott, C.L., Gomez Perdiguero, E., Geissmann, F., Henri, S., Malissen, B., Osborne, L.C., Artis, D., and Mowat, A.M. (2014). Constant replenishment from circulating monocytes maintains the macrophage pool in the intestine of adult mice. Nat. Immunol. *15*, 929–937.

Bennett, M., Bennett, C., Liddelow, S., Ajami, B., Zamanian, J., Fernhoff, N., Mulinyawe, S., Bohlen, C., Adil, A., Tucker, A., et al. (2016). New tools for studying microglia in the mouse and human CNS. Proc. Natl. Acad. Sci. USA *113*, E1738–E1746.

Beutner, C., Linnartz-Gerlach, B., Schmidt, S.V., Beyer, M., Mallmann, M.R., Staratschek-Jox, A., Schultze, J.L., and Neumann, H. (2013). Unique transcriptome signature of mouse microglia. Glia *61*, 1429–1442.

Buchrieser, J., James, W., and Moore, M. (2017). Human induced pluripotent stem cell-derived macrophages share ontogeny with MYB-independent tissue-resident macrophages. Stem Cell Rep. *8*, 334–345.

Butovsky, O., Jedrychowski, M.P., Moore, C.S., Cialic, R., Lanser, A.J., Gabriely, G., Koeglsperger, T., Dake, B., Wu, P.M., Doykan, C.E., et al. (2014). Identification of a unique TGF-beta-dependent molecular and functional signature in microglia. Nat. Neurosci. *17*, 131–143.

Calderon, B., Carrero, J.A., Ferris, S.T., Sojka, D.K., Moore, L., Epelman, S., Murphy, K.M., Yokoyama, W.M., Randolph, G.J., and Unanue, E.R. (2015). The pancreas anatomy conditions the origin and properties of resident macrophages. J. Exp. Med. *212*, 1497–1512.

Epelman, S., Lavine, K.J., Beaudin, A.E., Sojka, D.K., Carrero, J.A., Calderon, B., Brija, T., Gautier, E.L., Ivanov, S., Satpathy, A.T., et al. (2014). Embryonic and adult-derived resident cardiac macrophages are maintained through distinct mechanisms at steady state and during inflammation. Immunity *40*, 91–104.

Etemad, S., Zamin, R.M., Ruitenberg, M.J., and Filgueira, L. (2012). A novel in vitro human microglia model: characterization of human monocyte-derived microglia. J. Neurosci. Methods *209*, 79–89.

Ginhoux, F., Greter, M., Leboeuf, M., Nandi, S., See, P., Gokhan, S., Mehler, M., Conway, S., Ng, L., Stanley, R., et al. (2010). Fate mapping analysis reveals that adult microglia derive from primitive macrophages. Science *330*, 841–845.

Ginhoux, F., Lim, S., Hoeffel, G., Low, D., and Huber, T. (2013). Origin and differentiation of microglia. Front. Cell Neurosci. *7*, 45.

Gomez Perdiguero, E., Klapproth, K., Schulz, C., Busch, K., Azzoni, E., Crozet, L., Garner, H., Trouillet, C., de Bruijn, M.F., Geissmann, F., et al. (2015). Tissue-resident macrophages originate from yolk-sac-derived erythro-myeloid progenitors. Nature *518*, 547–551.

Greter, M., Lelios, I., Pelczar, P., Hoeffel, G., Price, J., Leboeuf, M., Kundig, T.M., Frei, K., Ginhoux, F., Merad, M., et al. (2012). Stroma-derived interleukin-34 controls the development and maintenance of Langerhans cells and the maintenance of microglia. Immunity *37*, 1050–1060.

Guilliams, M., Ginhoux, F., Jakubzick, C., Naik, S., Onai, N., Schraml, B., Segura, E., Tussiwand, R., and Yona, S. (2014). Dendritic cells, monocytes and macrophages: a unified nomenclature based on ontogeny. Nat. Rev. Immunol. *14*, 571–578.

Gupta, R., Meissner, T., Cowan, C., and Musunuru, K. (2016). Genome-edited human pluripotent stem cell–derived macrophages as a model of reverse cholesterol transport—brief report. Arterioscler. Thromb. Vasc. Biol. *36*, 15–18.

Hancock, S., Finkelstein, D., and Adlard, P. (2014). Glia and zinc in ageing and Alzheimer's disease: a mechanism for cognitive decline? Front. Aging Neurosci. *6*, 137.

Hoeffel, G., and Ginhoux, F. (2015). Ontogeny of tissue-resident macrophages. Front. Immunol. *6*, 486.

Hoeffel, G., Chen, J., Lavin, Y., Low, D., Almeida, F.F., See, P., Beaudin, A.E., Lum, J., Low, I., Forsberg, E.C., et al. (2015). C-Myb(+) erythro-myeloid progenitor-derived fetal monocytes give rise to adult tissue-resident macrophages. Immunity *42*, 665–678.

Jiang, Y., Cowley, S., Siler, U., Melguizo, D., Tilgner, K., Browne, C., Dewilton, A., Przyborski, S., Saretzki, G., James, W., et al. (2012). Derivation and functional analysis of patient-specific induced pluripotent stem cells as an in vitro model of chronic granulomatous disease. Stem Cells *30*, 599–611.

Kapellos, T., Taylor, L., Lee, H., Cowley, S., James, W., Iqbal, A., and Greaves, D. (2016). A novel real time imaging platform to quantify macrophage phagocytosis. Biochem. Pharmacol. *116*, 107–119.

Karlsson, K., Cowley, S., Martinez, F., Shaw, M., Minger, S., and James, W. (2008). Homogeneous monocytes and macrophages

from human embryonic stem cells following coculture-free differentiation in M-CSF and IL-3. Exp. Hematol. *36*, 1167–1175.

Kierdorf, K., Erny, D., Goldmann, T., Sander, V., Schulz, C., Perdiguero, E.G., Wieghofer, P., Heinrich, A., Riemke, P., Hölscher, C., et al. (2013). Microglia emerge from erythromyeloid precursors via Pu.1- and Irf8-dependent pathways. Nat. Neurosci. *16*, 273–280.

Lee, H., James, W., and Cowley, S. (2017). LRRK2 in peripheral and central nervous system innate immunity: its link to Parkinson's disease. Biochem. Soc. Trans. *45*, 131–139.

Melief, J., Koning, N., Schuurman, K., Van De Garde, M., Smolders, J., Hoek, R., Van Eijk, M., Hamann, J., and Huitinga, I. (2012). Phenotyping primary human microglia: tight regulation of LPS responsiveness. Glia *60*, 1506–1517.

Monier, A., Adle-Biassette, H., Delezoide, A.L., Evrard, P., Gressens, P., and Verney, C. (2007). Entry and distribution of microglial cells in human embryonic and fetal cerebral cortex. J. Neuropathol. Exp. Neurol. *66*, 372–382.

Moore, A.R., Zhou, W.L., Jakovcevski, I., Zecevic, N., and Antic, S.D. (2011). Spontaneous electrical activity in the human fetal cortex in vitro. J. Neurosci. *31*, 2391–2398.

Muffat, J., Li, Y., Yuan, B., Mitalipova, M., Omer, A., Corcoran, S., Bakiasi, G., Tsai, L.-H., Aubourg, P., Ransohoff, R., et al. (2016). Efficient derivation of microglia-like cells from human pluripotent stem cells. Nat. Med. *22*, 1358–1367.

O'Rourke, J.G., Bogdanik, L., Yáñez, A., Lall, D., Wolf, A.J., Muhammad, A.K., Ho, R., Carmona, S., Vit, J.P., Zarrow, J., et al. (2016). C9orf72 is required for proper macrophage and microglial function in mice. Science *351*, 1324–1329.

Ohgidani, M., Kato, T.A., Setoyama, D., Sagata, N., Hashimoto, R., Shigenobu, K., Yoshida, T., Hayakawa, K., Shimokawa, N., Miura, D., et al. (2014). Direct induction of ramified microglia-like cells from human monocytes: dynamic microglial dysfunction in Nasu-Hakola disease. Sci. Rep. *4*, 4957.

Palis, J., Robertson, S., Kennedy, M., Wall, C., and Keller, G. (1999). Development of erythroid and myeloid progenitors in the yolk sac and embryo proper of the mouse. Development *126*, 5073–5084.

Pandya, H., Shen, M., Ichikawa, D., Sedlock, A., Choi, Y., Johnson, K., Kim, G., Brown, M., Elkahloun, A., Maric, D., et al. (2017). Differentiation of human and murine induced pluripotent stem cells to microglia-like cells. Nat. Neurosci. *20*, 753–759.

Ransohoff, R. (2016). Neuroinflammation: surprises from the sanitary engineers. Nature *532*, 185–186.

Rezaie, P., Dean, A., Male, D., and Ulfig, N. (2005). Microglia in the cerebral wall of the human telencephalon at second trimester. Cereb. Cortex *15*, 938–949.

Russo, I., Bubacco, L., and Greggio, E. (2014). LRRK2 and neuroinflammation: partners in crime in Parkinson's disease? J. Neuroinflammation *11*, 52.

Schilling, T., Nitsch, R., Heinemann, U., Haas, D., and Eder, C. (2001). Astrocyte-released cytokines induce ramification and outward K+ channel expression in microglia via distinct signalling pathways. Eur. J. Neurosci. *14*, 463–473.

Schulz, C., Perdiguero, E., Chorro, L., Szabo-Rogers, H., Cagnard, N., Kierdorf, K., Prinz, M., Wu, B., Sten, E., Pollard, J., et al. (2012). A lineage of myeloid cells independent of Myb and hematopoietic stem cells. Science *336*, 86–90.

Schwartz, M.P., Hou, Z., Propson, N.E., Zhang, J., Engstrom, C.J., Santos Costa, V., Jiang, P., Nguyen, B.K., Bolin, J.M., Daly, W., et al. (2015). Human pluripotent stem cell-derived neural constructs for predicting neural toxicity. Proc. Natl. Acad. Sci. USA *112*, 12516–12521.

Shi, Y., Kirwan, P., and Livesey, F.J. (2012). Directed differentiation of human pluripotent stem cells to cerebral cortex neurons and neural networks. Nat. Protoc. *7*, 1836–1846.

Tamoutounour, S., Guilliams, M., Montanana Sanchis, F., Liu, H., Terhorst, D., Malosse, C., Pollet, E., Ardouin, L., Luche, H., Sanchez, C., et al. (2013). Origins and functional specialization of macrophages and of conventional and monocyte-derived dendritic cells in mouse skin. Immunity *39*, 925–938.

Tavian, M., and Peault, B. (2005). Embryonic development of the human hematopoietic system. Int. J. Dev. Biol. *49*, 243–250.

van Wilgenburg, B., Browne, C., Vowles, J., and Cowley, S. (2013). Efficient, long term production of monocyte-derived macrophages from human pluripotent stem cells under partly-defined and fully-defined conditions. PLoS One *8*, e71098.

Vanhee, S., De Mulder, K., Van Caeneghem, Y., Verstichel, G., Van Roy, N., Menten, B., Velghe, I., Philippe, J., De Bleser, D., Lambrecht, B.N., et al. (2015). In vitro human embryonic stem cell hematopoiesis mimics MYB-independent yolk sac hematopoiesis. Haematologica *100*, 157–166.

Villegas-Llerena, C., Phillips, A., Garcia-Reitboeck, P., Hardy, J., and Pocock, J. (2016). Microglial genes regulating neuroinflammation in the progression of Alzheimer's disease. Curr. Opin. Neurobiol. *36*, 74–81.

Zhang, Y., Sloan, S., Clarke, L., Caneda, C., Plaza, C., Blumenthal, P., Vogel, H., Steinberg, G., Edwards, M., Li, G., et al. (2016). Purification and characterization of progenitor and mature human astrocytes reveals transcriptional and functional differences with mouse. Neuron *89*, 37–53.

Stem Cell Reports, Volume *8*

Supplemental Information

A Highly Efficient Human Pluripotent Stem Cell Microglia Model Displays a Neuronal-Co-culture-Specific Expression Profile and Inflammatory Response

Walther Haenseler, Stephen N. Sansom, Julian Buchrieser, Sarah E. Newey, Craig S. Moore, Francesca J. Nicholls, Satyan Chintawar, Christian Schnell, Jack P. Antel, Nicholas D. Allen, M. Zameel Cader, Richard Wade-Martins, William S. James, and Sally A. Cowley

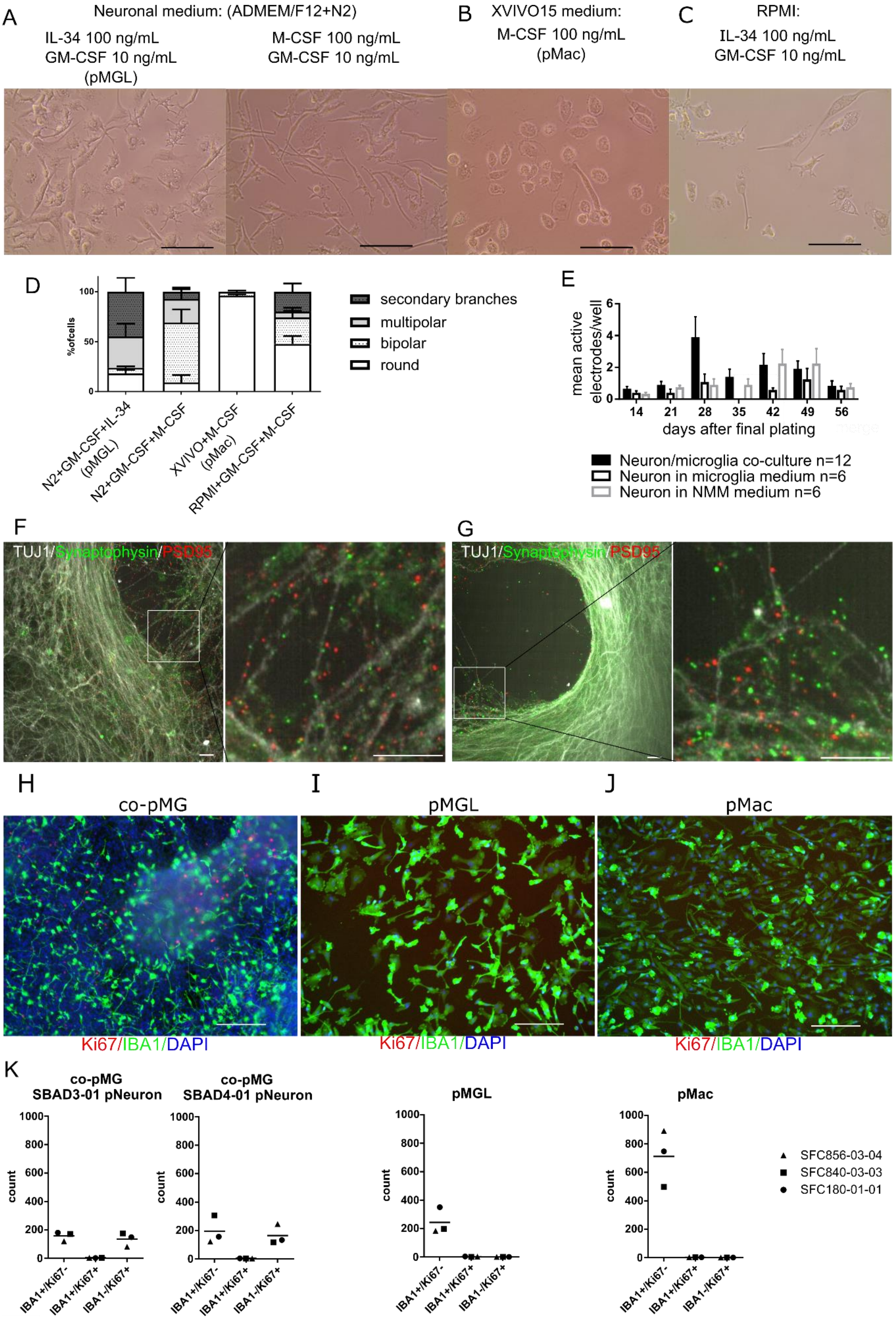

Figure S1 Further characterisation of macrophage and microglia cultures

(A-D) Identification of basal media and growth factors that promote ramified microglial morphology.pMacpre were differentiated for 17 days with different basal media and growth factors, as indicated. (A) Neuronal medium. (B) Our standard iPSC-macrophage medium (van Wilgenburg et al., 2013). (C) Medium described previously to promote microglia-like morphology in blood monocytes (Etemad et al., 2012). Representative phase contrast images of each condition show that ADMEM/F12 + N2 + 100 ng/mL IL-34 + 10 ng/mL GM-CSF promotes the most ramified microglia-like morphology. Scale bar 50 µm. (D) Quantification of morphology. Secondary branching was considered indicative of a ramified microglia-like morphology. Mean of 3 images per condition, error bars represent SEM. (E-G) Neuronal electrical functionality and synaptic markers in co-cultures. (E) Spontaneous electrical activity of cultures detected using a multi-electrode array (12 electrodes per well, n = number of wells, error bars represent SD). Neurons show spontaneous electrical activity from the beginning of co-culture, which increases modestly over an extended time period. Electrical activity is not inhibited by the presence of co-pMG. Note that electrical activity can only be detected from neurons that are in contact with the electrodes, and since the neurons form clumps, especially at longer culture times, not all replicate wells record activity. (F, G) The presynaptic marker Synaptophysin and the postsynaptic marker PSD95 can be detected in co-cultures (F) and neuron monoculture (G) (images taken at day 15 of co-culture, scalebar 20 µm). See also Video S1, showing calcium flux upon K^+ stimulation. (H-K) Continued Proliferation of iPSC-neuronal progenitors but rarely of microglia or macrophages. (H-J) Cultures were stained for the proliferation marker Ki-67 (Red) and IBA1 (macrophages/microglia, green) after 3 weeks. (H) Co-culture of SFC840-03-01 microglia with SBAD3-01 neurons. (I) SFC840-03-03 macrophages cultured in microglia medium. (J) SFC840-03-03 macrophages cultured in macrophage medium. Scale bar 100 µm. (K) Quantification of IBA1 and Ki67 signal in microglia/macrophages of 3 iPSC lines in co-culture with 2 different pNeurons (SBAD3-01, SBAD4-01) or in monoculture. In neuronal clusters the DAPI signal was too dense for quantification of the number of neurons, but is expected to be >3000 neurons/image.

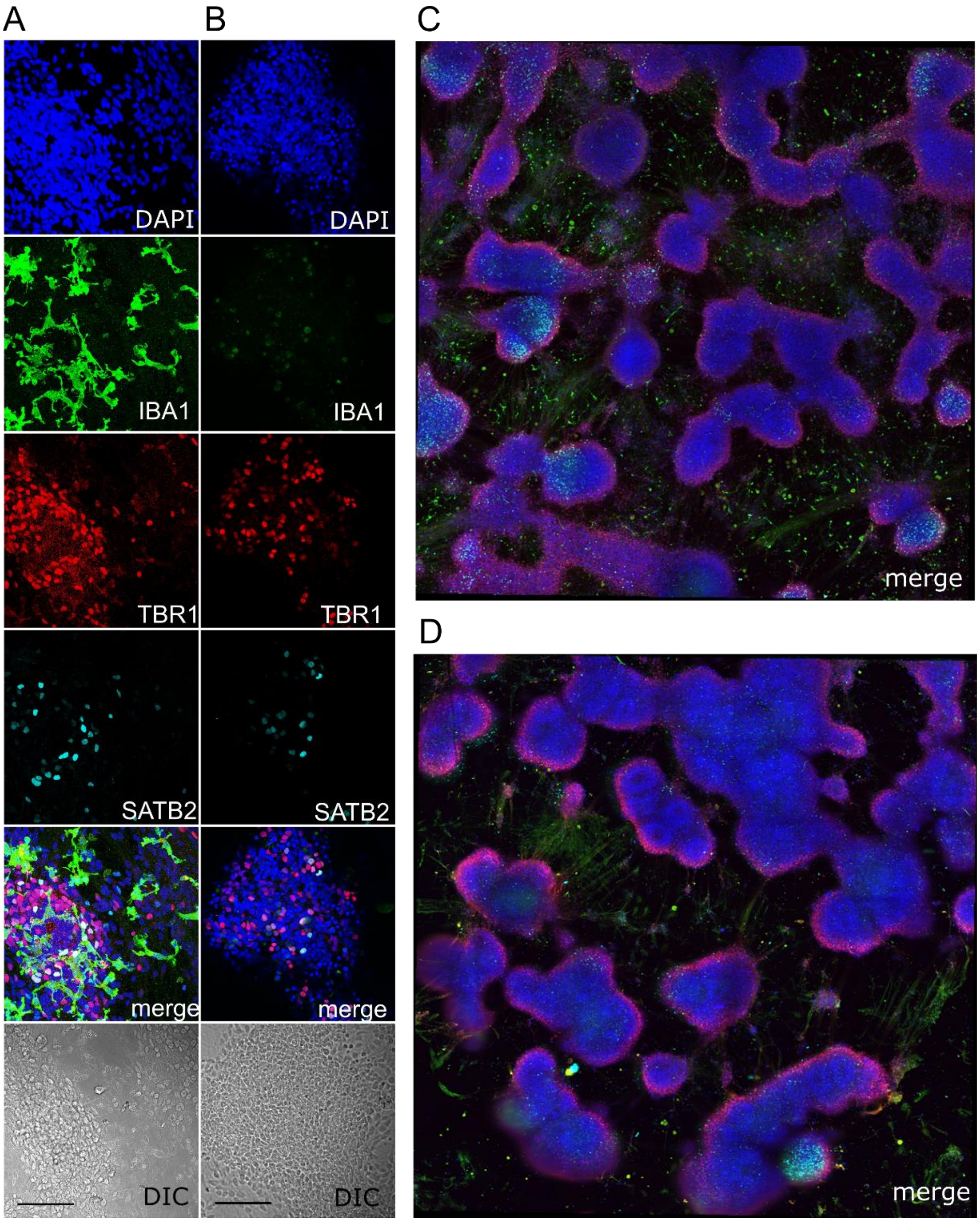

Figure S2 iPSC-microglia-neuronal co-cultures express deep layer (TBR1) and upper layer (SATB2) cortical markers and are stable for extended periods of co-culture (Relates to Figure 1)

(A) 14 day co-culture. (B) 14 day neuron-only culture. (C) 39 day co-culture (IBA1, SATB2, TBR1, DAPI). (D) 39 day neuron-only culture. Scale bar 50 µm.

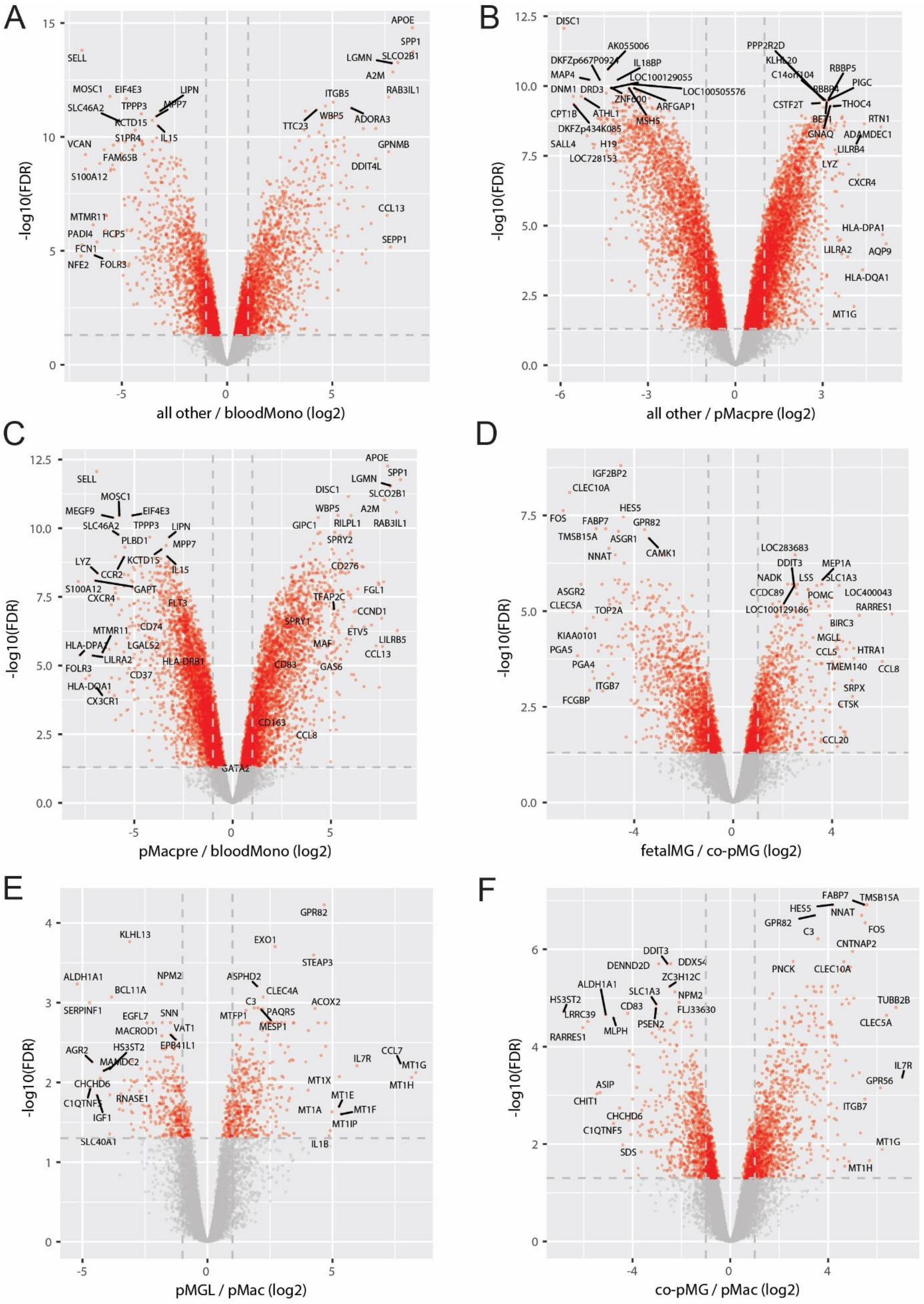

Supplementary Figure S3 Differential expression analyses (Relates to Figure 2)

Volcano plots shows genes differentially expressed between (A) bloodMono vs all other samples (to explore PC2 in Figure 2A); (B) pMacpre vs all other samples (to explore PC1 in Figure 2A); (C-F) other comparisons of interest

as indicated. Horizontal dashed lines indicate an adjusted p- value of 0.05. Vertical dashed lines indicate a two-fold difference in expression.

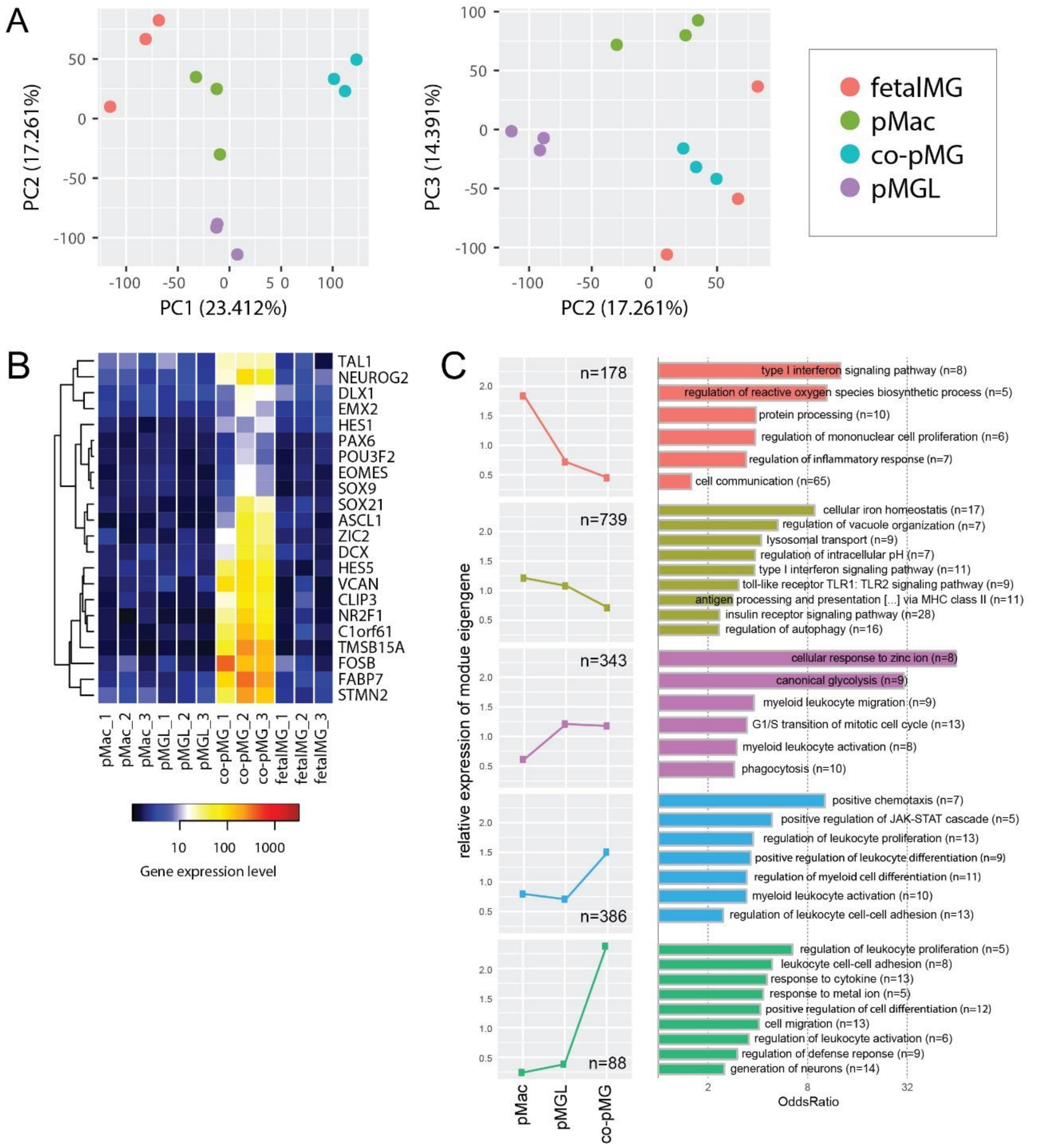

Supplementary Figure S4 Transcriptomics analysis of macrophage populations (Relates to Figure 2)

(A) PCA analysis of gene expression. Inspection of the proportion of variance scree plot identified three important components (data not shown). GO analysis revealed genes positively loading the first principle component (PC1, 23.4% of variance) to contain annotation categories associated with neural cells (data not shown). This signature is associated with co-pMG which, given their close association with neural cells whilst in co-culture, likely reflects a low level of neural cell derived contamination. Reassuringly, sample projection based on PC2 and PC3 (together explaining 31.7% of variance, right panel) demonstrated the similarity of the co-pMG and fetalMG samples. (B) The heatmap shows examples of neural genes that positively contribute to PC1 (A). (C) Neuronal co-culture induces a microglia-like differentiation signature in iPSC derived cells. The figure shows k-means cluster profiles (left, line plots) and associated enriched biological processes (selected GO categories, adjusted p-value < 0.05) (right, bar graphs). Weakly detected genes were excluded from the analysis to limit the impact of transcripts deriving from the apparent low-level neural cell derived contamination of the co-pMG sample. Genes with significantly variable expression (adjusted $p < 0.05$) between pMac, pMGL and co-pMG were used as the input for the kmeans clustering. Salmon red panel shows pathways strongly downregulated in co-pMG versus iMac,

green panel shows pathways strongly upregulated in co-pMG versus pMac, the intermediate panels show pathways moderately up- (blue, purple) or downregulated (green-grey) in co- pMG. In the line graph panels n = number of microarray probes, in the bar graph panels n = number of genes. Further details of the analyses are given in the Supplementary Experimental Procedures.

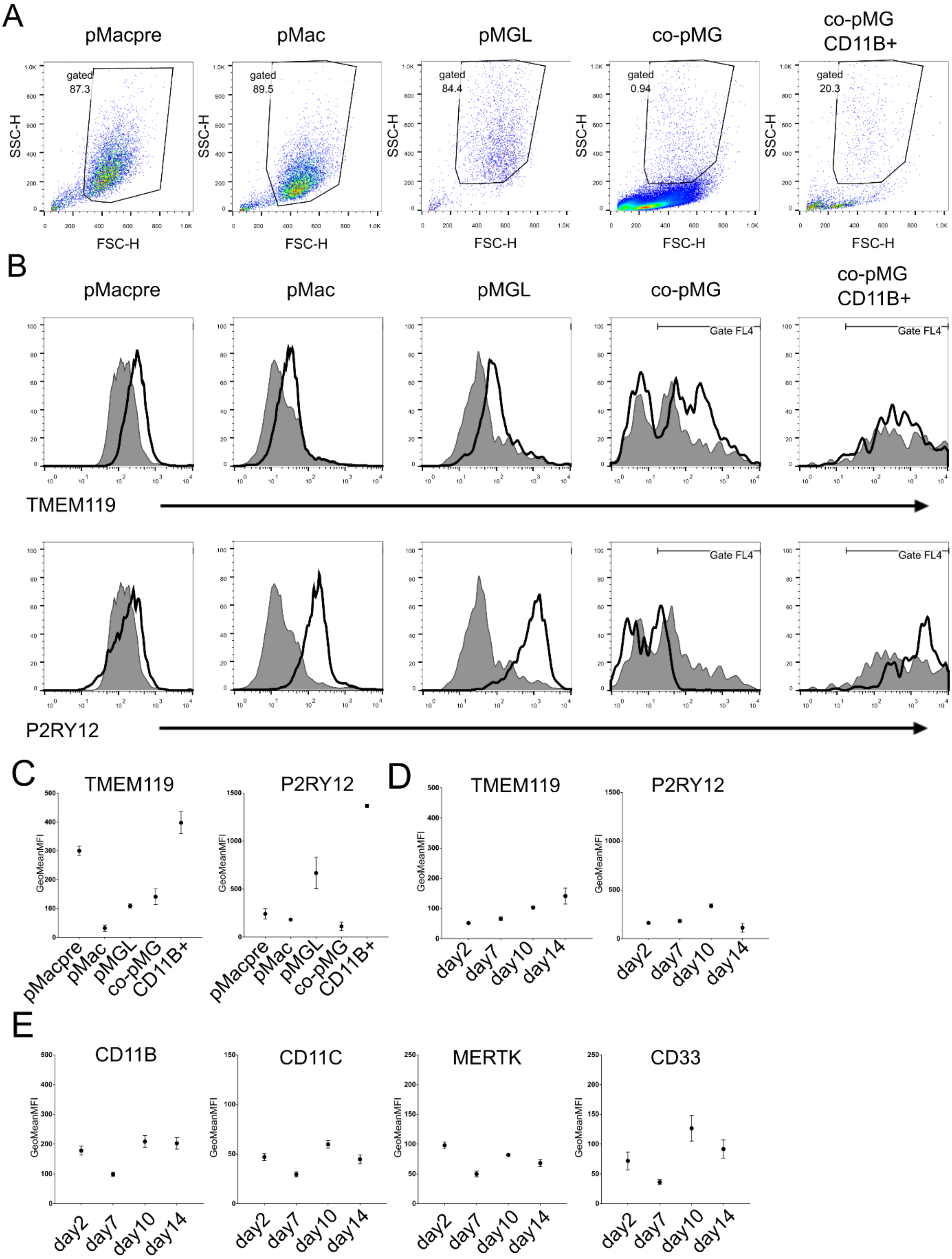

Figure S5 Additional flow cytometry (Relates to Figure 4)

(A) Forward Scatter FSC/Side Scatter SSC gating for Figure 4 and Figure S6. (B) FACS plots of microglia marker (black line) and 2nd antibody-only staining (grey) for SFC856-03-04 shows staining for TMEM119 and P2RY12 in monoculture but in co-culture non-specific background staining for 2nd antibody-only is apparent. (C) Mean Fluorescence Intensity of microglia markers (mean and SEM of 3 genetic backgrounds) in the different macrophage populations (D, E) Time-course of co-culture for microglia and monocyte/macrophage markers.

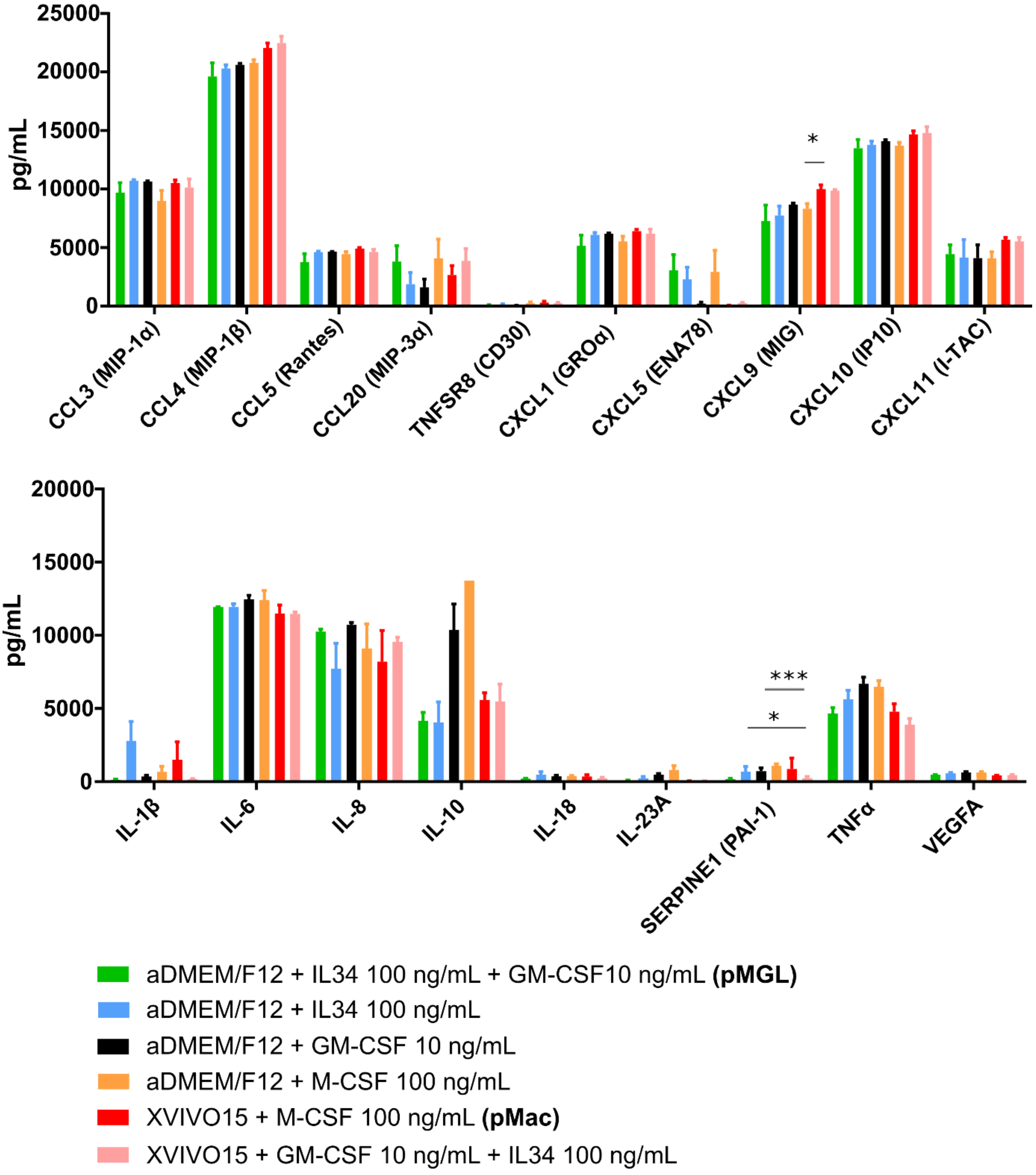

Figure S6 Effect of basal medium and cytokines on the inflammatory response to LPS/IFNγ (Relates to Figure 7)

Luminex multiplex array. Mean ± SEM of 3 biological replicates. Statistical analysis was performed with one way ANOVA followed by Tukey's multiple comparison test.

Table S1: Details of cells and materials used in this study

iPSC lines used in this study (All from disease-free donors)					
ID of fibroblast	ID of iPSC clone	Gender	Age of Biopsy (years)	Reprogramming method	iPSC clone characterised
SF180	SFC180-01-01	female	60	Cytotune1	Haenseler in submission
SF840	SFC840-03-01	female	67	Cytotune1	(Fernandes et al., 2016)
	SFC840-03-03				
SF856	SFC856-03-04	female	78	Cytotune1	Haenseler in submission
SBAD3	SBAD3-01	female	36	Cytotune1	Melguzo in submission
SBAD4	SBAD4-01	male	80	Cytotune1	Melguzo in submission
AH016	AH016-3	male	80	rv SO^{3}KMN	(Sandor et al., 2017)
	AH016-3 Lenti_RFP_IP (11 copy)				This study

Microglia medium (for pMGL, co-pMG and pNeuron)				
	Final conc	Stock conc	Supplier	Cat no.
Advanced DMEM/F12	1x	1x	Life Technologies	12634-010
N2 supplement	1x	100x	Life Technologies	17502-048
GlutaMAX^{TM}	2mM	200mM	Life Technologies	35050-061
2-mercaptoethanol	50µM	50mM	Life Technologies	31350-010
Pen/Strep	50U/mL	100x	Life Technologies	17502-048
IL-34	100ng/mL	100ug/mL	Peprotech	200-34
GM-CSF	10ng/mL	10ug/mL	Life Technologies	PHC2013
Growth-factor reduced Matrigel (undefined product for coating plate for co-pMG and pNeuron)	83-fold dilution of supplied stock		Scientific Laboratory Supplies	354277

Neuronal maintenance medium (NMM) (for differentiation until start of co-culture and for pNeuron (Shi et al., 2012))				
	Final conc	Stock conc	Supplier	Cat no.
Neurobasal	1x	1x	Life Technologies	21103-049
Advanced DMEM/F12	1x	1x	Life Technologies	12634-010
B27 supplement	0.5x	100x	Life Technologies	17504-044
N2 supplement	0.5x	100x	Life Technologies	17502-048
GlutaMAX^{TM}	2mM	200mM	Life Technologies	35050-061
2-mercaptoethanol	50µM	50mM	Life Technologies	31350-010
Pen/Strep	50U/mL	100x	Life Technologies	17502-048
Insulin	5ug/mL		Sigma	I6634
Growth-factor reduced Matrigel (undefined product for coating plate)	83-fold dilution of supplied stock		Scientific Laboratory Supplies	354277

Macrophage differentiation medium (for pMac (van Wilgenburg et al., 2013))				
	Final conc	Stock conc	Supplier	Cat no.
X-VIVO 15	1x	1x	Lonza	BE04-418
GlutaMAX^{TM}	2mM	200mM	Life Technologies	35050-061
2-mercaptoethanol	50µM	50mM	Life Technologies	31350-010

Pen/Strep	50U/mL	100x	Life Technologies	17502-048
M-CSF	100ng/mL	100µg/mL	Gibco	PHC 9501

Composition of N2 and B27 supplements
(*components that are potentially immunosuppressive / stress buffers)

**N2 Supplement**	Conc in 100x	**B27 supplement**	Conc in 50x
Human Transferrin (Holo)	1mM	DL Alpha Tocopherol Acetate	Concentrations not given by manufacturer
Insulin Recombinant Full Chain*	0.086mM	DL Alpha-Tocopherol	
Progesterone*	0.002mM	Vitamin A (acetate)	
Putrescine*	10mM	BSA, fatty acid free Fraction V	
Selenite	0.003mM	Catalase*	
		Human Recombinant Insulin*	
		Superoxide Dismutase*	
		Corticosterone*	
		D-Galactose	
		Ethanolamine HCl	
		Glutathione (reduced)	
		L-Carnitine HCl	
		Linoleic Acid	
		Linolenic Acid	
		Progesterone*	
		Putrescine 2HCl*	
		Sodium Selenite	
		T3 (triodo-I-thyronine)	

Antibodies used for Immunocytochemistry

Primary	Species/ clonality	Manu-facturer	Cat. No.	Secondary	Fluorophore	Manu-facturer	Cat. No.
IBA1	goat/ poly	abcam	ab5076	donkey-αgoat	Alexa488	Thermo Fisher	A11055
TUJ1	mouse/ mono	Covance	MMS-435P	donkey-αmouse	Alexa647	Thermo Fisher	A10042
TUJ1	rabbit	Covance	MRB-435P	Donkey-αrabbit	Alexa568	Thermo Fisher	A31571
GFAP	rabbit/ poly	DAKO	ZO334	donkey-αrabbit	Alexa568	Thermo Fisher	A31571
TBR1	rabbit/ poly	Abcam	ab31940	donkey-αrabbit	Alexa568	Thermo Fisher	A31571
SATB2	mouse/ mono	Abcam	ab51502	donkey-αmouse	Alexa647	Thermo Fisher	A10042
NESTIN	mouse/ mono	Abcam	ab22035	donkey-αmouse	Alexa647	Thermo Fisher	A10042

PAX6	rabbit/ poly	Covance	PRB-278P	donkey-αrabbit	Alexa568	Thermo Fisher	A31571
SYNAPTO-PHYSIN	guinea pig/ poly	Synaptic Systems	101 004	goat-α-guinea pig	Alexa488	Thermo Fisher	A11073
PSD95	mouse/ mono	Thermo Fisher	MA1-045	donkey-αmouse	Alexa647	Thermo Fisher	A10042
Ki67	mouse/ mono	Merck Millipore	MAB4190	donkey-αmouse	Alexa647	Thermo Fisher	A10042
TMEM119	rabbit/ poly	abcam	ab185333	donkey-αrabbit	Alexa568	Thermo Fisher	A31571
P2RY12	rabbit/ mono	abcam	ab188968	donkey-αrabbit	Alexa568	Thermo Fisher	A31571
IgG	rabbit/ poly	abcam	ab27478	donkey-αrabbit	Alexa568	Thermo Fisher	A31571
MERTK	mouse/ mono	abcam	Ab52591	donkey-αmouse	Alexa647	Thermo Fisher	A10042
IgG1	Mouse/ mono	AbD serotec	MCA928	donkey-αmouse	Alexa647	Thermo Fisher	A10042

Fluorophore conjugated antibodies used for Flow Cytometry

Marker	Fluorophore	Isotype	Manufacturer	Cat. No. marker	Cat. No. isotype control
CD11b	APC	mouse IgG1-Κ	Biolegend	301309	400119
CD11C	FITC	mouse IgG2a	ImmunoTools	21487113	21335023
CD14	PE	mouse IgG1	ImmunoTools	21620144	21335014
CD45	APC	mouse IgG1	ImmunoTools	21270456	21275516
HLA-DR	FITC	mouse IgG2a	ImmunoTools	21278993	21335023
CX3CR1	APC	rat IgG2b-Κ	Biolegend	341609	400611
CD33	APC	mouse IgG1	eBioscience	17-0338-42	17-4717-41
MERTK	Alexa647	mouse IgG1-Κ	Biolegend	367606	400130

Primers used for qRT-PCR of microglia markers

	Forward primer sequence: (5' to 3')	Reverse primer sequence: (5' to 3')	supplier
C1QA	GTGACACATGCTCTAAGAAG	GACTCTTAAGCACTGGATTG	Sigma Aldrich
GAS6	CGAAGAAACTCAAGAAGCAG	AGACCTTGATCTCCATTAGG	Sigma Aldrich
GPR34	GAAGACAATGAGAAGTCATACC	TGTTGCTGAGAAGTTTTGTG	Sigma Aldrich
PROS1	AAAGATGTGGATGAATGCTC	TCACATTCAAAATCTCCTGG	Sigma Aldrich
MERTK	AGGACTTCCTCACTTTACTAAG	TGAACCCAGAAAATGTTGAC	Sigma Aldrich
P2RY12	AAGAGCACTCAAGACTTTAC	GGGTTTGAATGTATCCAGTAAG	Sigma Aldrich
TMEM119	AGTCCTGTACGCCAAGGAAC	GCAGCAACAGAAGGATGAGG	Sigma Aldrich
TREM2	TCTGAGAGCTTCGAGGATGC	GGGGATTTCTCCTTCAAGA	Sigma Aldrich
18S	Sequences not provided by supplier		Eurogentec

Table S2 Initial screen with Proteome Profiler™ Human XL Cytokine Array (Relates to Figure 7)

Released factor	pMac unstim	pMac LPS/IFNγ	co-pMG unstim	co-pMG LPS/IFNγ
Adiponectin	2870	1970	4640	2960
Aggrecan	9375	7010	7080	9105
**Angiogenin**	21250	14100	106500	65350
Angiopoietin-1	2365	2540	4475	5655
Angiopoietin-2	4655	4095	7020	8695
BAFF	4385	3485	4870	10725
BDNF	3010	1510	3855	4140
**C5/C5a**	2420	12600	4645	19300
CCL2 (MCP-1)	116500	144500	148000	143500
**CCL3/4 (MIP-1α/MIP-1β)***	3260	155500	3230	18200
**CCL5 (RANTES)***	3120	115500	3930	4665
**CCL7 (MCP-3)***	3975	48200	6505	51700
CCL17 (SDF-1α)	4525	6120	9840	10320
CCL17 (TARC)	3130	2035	3255	3070
**CCL19 (MIP-3β)***	2435	14350	2765	9790
**CCL20 (MIP-3α)***	1760	99650	2570	6895
**CD14**	31450	24200	46350	38900
CD40Ligand (CD154)	3425	2340	4855	4795
Chitinase 3-like 1	250500	236000	229000	209000
Complement Factor D	16100	10940	10130	13200
C-Reactive Protein	2665	5360	5040	7775
Cripto-1	2360	2320	3510	3080
**CXCL1 (GRO-α)***	3515	101900	62050	102100
CXCL4 (PF4)	2045	162	1895	2900
**CXCL5 (ENA-78)***	2225	3960	166000	102000
**CXCL9 (MIG)***	2935	128000	3620	55100
**CXCL10 (IP-10)***	3720	172500	3620	156500
**CXCL11 (I-TAC)***	2970	231000	1825	119000
Cytostatin C	35500	22950	55400	34800
Dkk-1	2475	3135	2780	2675
DPPIV (CD26)	21700	47700	42050	16045
**EGF**	3455	2880	6240	6510
**EMMPRIN (CD147)**	12050	20800	21000	4105
Endoglin (CD105)	8365	8665	9940	7470
**Fas Ligand (CD178)**	2970	1925	3015	5575
**FGF basic**	3850	9590	6925	11950
**FGF-7**	2275	966	3385	5385
**FGF19**	14900	24600	24250	35250
**Flt-3 Ligand**	1915	1355	3395	1520
**G-CSF**	1330	4840	2765	6175
GDF-15	7325	10570	6195	5790
**GM-CSF(spiked in co-pMG)**	4335	4860	31950	13350
**Growth hormone**	1340	1340	4215	1910
**HGF**	2245	7675	4780	7715
**ICAM-1 (CD54)**	10900	26600	30950	38400
**IGFBP-2**	2135	1147	124500	91850
IGFBP-3	3540	4555	8625	7430
**IL-1-ra**	15745	68350	47400	25300
**IL-1α**	4585	4540	6940	9825
IL-1β*	3535	6535	4740	5630
IL-2	2995	3025	4645	2610
IL-3	1565	1102	2335	1930
**IL-4**	4970	8225	7430	9435
IL-5	2190	909	2330	2055

**IL-6***	4440	107500	6840	102000
**IL-8 (CXCL8)***	63150	122000	149000	136000
**IL-10***	4530	19900	7565	47850
**IL-11**	5835	7365	9115	10850
**IL-12p70**	2915	4310	4290	5660
**IL-13**	2135	2525	4410	6250
**IL-15**	2690	4040	3085	6725
**IL-16**	2145	2405	2715	5860
**IL-17A**	14600	33250	18700	28150
**IL-18 Bpa***	2180	10600	3125	9030
**IL-19**	2635	2645	3145	39600
IL-22	5120	9720	6410	8270
**IL-23***	1830	13250	1468	5400
IL-24	2355	4260	3705	4155
IL-27	3760	9640	2390	5680
IL-31	1770	2089	2735	2510
IL-32α/β/γ	2510	4625	2585	4175
**IL-33**	754	2125	2675	4035
**IL-34 (spiked in co-pMG)**	2130	806	14100	3980
INFγ (spiked in LPS/IFNγ)*	8420	109000	8100	104500
Kallikrein 3	1695	2840	3860	1747
Leptin	3430	2940	3275	4620
LIF	2555	1565	1750	4270
**Lipocalin-2**	7785	8250	2320	3675
**M-CSF (spiked in pMac)**	137500	133500	5250	10725
**MIF***	20550	11450	70800	72250
**MMP-9**	56950	55100	76150	19100
Myeloperoxidase	2530	322	1605	1641
Osteopontin	107750	106000	111000	105750
**PDGF-AA**	9715	19200	5195	7080
**PDGF-AB/BB**	5615	4865	1200	1515
**Pentraxin-3**	5150	3310	12350	19050
RAGE	2320	825	2880	3295
**RBP4**	315500	355000	4180	4565
Relaxin-2	2590	2070	1510	3035
Resistin	5670	5420	7495	5515
**SerpinE1 (PAI-1)***	4630	12000	104000	87350
SHBG	3675	4675	5120	4790
ST2	2680	1930	2255	3760
TFF3	5340	3845	2950	3865
TfR (CD71)	3380	2025	3970	4475
TGF-α	2865	2230	3735	3980
**TNFRSF8 (CD30)***	2445	645	4800	2090
**TNF-α***	3645	103500	1960	8195
Thrombospondin-1	1455	-130	2950	769
**uPAR (CD87)**	6895	51050	14250	6130
**VEGF***	1590	147	36350	3315
Vitamin D BP	8660	10600	6240	7865

Legend Table S2

Results were quantified with Image Studio Lite. Numbers show the mean luminescence signal of two dots per factor (n=2). Factors that are substantially differentially released between pMac and co-pMG or upon stimulation with LPS/IFNγ are in bold font. Negative values are where measurement is below background luminescence. * are followed up in Figure 7.

Legend Table S3 Transcriptomics data and differential expression results

The Excel file contains the normalised probe level expression data and the full results of the differential expression analyses presented in Supplementary Figure S3. Further details of these analyses are given in the Supplementary Experimental Procedures.

Supplemental Experimental Procedures

iPSC lines

The derivation and characterisation of the iPSC lines used in this study is described elsewhere (Fernandes et al., 2016; Sandor et al., 2017) Haenseler, submitted, Melguzo in preparation), see Table S1. All lines were derived from dermal fibroblasts from disease-free donors recruited through StemBANCC (SF180, SF856) (Morrison et al., 2015), or the Oxford Parkinson's Disease Centre (SF840, AH016): participants were recruited to this study having given signed informed consent, which included derivation of hiPSC lines from skin biopsies (Ethics Committee: National Health Service, Health Research Authority, NRES Committee South Central, Berkshire, UK, who specifically approved this part of the study (REC 10/H0505/71), or from fibroblasts purchased from Lonza (SBAD3, SBAD4), who provide the following ethics statement: 'These cells were isolated from donated human tissue after obtaining permission for their use in research applications by informed consent or legal authorization.' iPSC were cultured in mTeSR™1 (StemCell Technologies), on hESC-qualified Matrigel-coated plates (BD), passaging as clumps using 0.5 mM EDTA in PBS (Beers et al., 2012). Large-scale SNP-QCed batches were frozen at p15-25 and used for experiments within a minimal number of passages post-thaw to ensure consistency.

Generation and characterisation of the RFP expressing iPSC line AH016-3 Lenti_IP_RFP

To generate an iPSC line constitutively expressing RFP, AH016-3 was transduced with a second generation SIN lentiviral vector (LV-EF1a-RFP-IRES-Puromycin^R). Cells were kept under continuous puromycin selection (2 µg/mL: a concentration sufficient to kill untransduced cells). For single cell cloning AH016-3-RFP were plated at 10^4 per 10 cm dish on mitotically-inactivated mouse embryonic fibroblast feeder cells (MEF; outbred Swiss mice established and maintained at the Department of Pathology, Oxford (Chia, Achilli, Festing, & Fisher, 2005; Gardner, 1982)) on gelatin-coated tissue culture plates in hESC medium (KO-DMEM, 2 mmol/L GlutaMAX 100 mmol/L non-essential amino acids, 20% serum replacement (KO-SR), and 8 ng/mL basic fibroblastic growth factor (FGF2)), supplemented with 10 µmol/L Y-27632 on the day of the plating. After 7 days of expansion, individual single-cell colonies were picked manually onto a matrigel coated 96 well plate in mTeSR™1. Number of lentiviral integrants per clone was quantified using digital droplet PCR (ddPCR) copy number variation analysis (Bio-Rad QX200) according to manufacturer's protocol. Briefly, 2 µl of EcoRI-digested genomic DNA at 100 ng/µl was used with the EvaGreen Super Mix and 100 nM forward and reverse primers. The following RFP primers were used: JB-111 (5' - ATGCAGAAGAAAACACGCGG - 3') and JB-112 (5' - CCGGGCATCTTGAGGTTCTT - 3'). PCR primers for the *MYB* gene, were used as endogenous control: JB-71 (5' – ACAGGAAGGTTATCTGCAGGAGTCT – 3') and JB-72 (5' – AGTGGCAGGGAGTTGAGCTGTA - 3'). The iPSC clone used in this study has 11 lentiviral integrants.

Immunofluorescence

Cells were fixed with 4% paraformaldehyde (PFA) in PBS for 10 min, permeabilised with 0.3% Triton X-100 in PBS and blocked with 10% normal donkey serum (Sigma) for 1 hr, then incubated with primary antibodies in PBS, 5% normal donkey serum and 0.1% Triton X-100 overnight, washed 3 times with PBS and 0.3% Triton X-100, incubated with secondary antibodies in PBS 0.1% Triton X-100 and 5% normal donkey serum for 90 min, washed 3 times with PBS and 0.3% Triton X-100, stained with DAPI, washed once with PBS, overlaid with PBS and imaged with an EVOS fl auto microscope (AMG), FV1200 (Olympus) confocal microscope, OperaPhenix (PerkinElmer) or an IN Cell Analyzer 6000 (GE).

The antibodies used are listed in Table S3.

Transcriptome sample preparation and analysis

30 mL of peripheral blood was collected from 3 healthy adult volunteers, according to University of Oxford OHS policy document 1/03, with signed informed consent. PBMCs were isolated after density gradient centrifugation with Ficol-Paque PLUS (17-1440-03, GE Healthcare), and monocytes (bloodMono) were extracted with CD14 MACS beads (130-050-201, Miltenyi). iPSC-derived macrophage precursors (pMacpre), from 3 control lines, were collected and immediately lysed for RNA isolation. From the same harvest, cells were set up in macrophage differentiation medium for 2 weeks to obtain pMac, and in microglia medium to obtain pMGL (lysed directly in the well after 2 weeks to obtain RNA), or resuspended in microglia medium, added to SBAD3-01 neurons and differentiated to co-pMG for 2 weeks. Then co-culture was dissociated to a single cell suspension with StemPro accutase (StemCell technologies), and any remaining adherent microglia gently lifted with a cell scraper. Cells were passed through a 70 µm cell strainer (352350, BD Bioscience), then co-pMG were selected from the co-culture with CD11b MACS beads (130-093-634, Miltenyi Biotec) and lysed immediately for RNA isolation. RNA was extracted from lysates using an RNeasy kit (Qiagen) for Illumina HT12v4 transcriptome array analysis. For qPCR, additional samples were blood monocyte-derived macrophages (bloodMac), which were differentiated on tissue-culture-treated plates, for 1 week in macrophage medium and compared directly to the same donors' bloodMono (lysed straight after CD14 bead selection) and to pMac differentiated for 1 week.

RNA from human microglia was obtained from 3 individual human fetal samples (at pre-myelinating gestational ages of fetalMG_1 20, fetalMG_2 23, fetalMG_3_15 weeks) and one human adult sample, according to Durafourt et al. (Durafourt et al., 2013). Briefly, microglia were cultured *ex vivo* in DMEM supplemented with 5% FBS for 5-7 days prior to RNA isolation using standard Trizol^{TM} methods. All procedures related to the use of these cells followed established institutional (McGill University, Montreal, QC, Canada) and Canadian Institutes of Health Research guidelines for the use of human cells. A further sample of adult microglia RNA was also obtained from directly isolated human surgical brain material (age 51; UK: Re: An Investigation of Novel Proteins and Biomarkers in Surgically-Resected Tissue from Patients with Epilepsy. R&D Ref: 10815; REC Ref: 14/EE/1098; IRAS No: 144065). Brain material was papain-treated to obtain single cells, then panned with CD11b MACS beads and the positive population lysed immediately for RNA extraction, within 4 hours of surgical removal.

Microarray data were pre-processed with the Bioconductor beadarray package (Dunning et al., 2007) using the "neqc" method to normalise expression levels within and between samples. Annotations were sourced from the Bioconductor illuminaHumanv4.db package: probes with an assigned quality of "No match" were excluded from down-stream analysis. Following inspection of the data only the top two-thirds of probes (by maximum expression level) were retained for further analysis being considered to represent "expressed genes". PCA analysis of the normalised, scaled expression matrix was performed using the R "prcomp" function. Differential expression analysis was performed using the Limma Bioconductor package (Ritchie et al., 2015). The Benjamini-Hochberg (BH) multiple testing correction procedure was used to compute adjusted p-values.

K-means clustering analysis of gene expression in pMac, pMGL and co-pMG was based on a set of 1734 probes with high (>=1000 in at least 2 replicates) and significantly variable (overall F-test, Limma, BH adjusted p value < 0.05) expression in these samples. K-means clustering was performed on the matrix of mean-scaled gene expression levels (replicate samples first combined by median averaging) using the R "kmeans" function (nstarts=10000, iter.max=10000). Selection of cluster number was guided by scree-plot analysis of within-cluster sum of squares (not shown). The Bioconductor GOStats package (Falcon and Gentleman, 2007) was used to identify significantly over-enriched GO biological processes within each cluster (conditional test, p-value cutoff 0.01, gene universe comprised of the top two-thirds "expressed genes", adjusted p-value < 0.05).

RNA-seq data for human astrocytes, endothelial cells myeloid cells, neurons and oligodendrocytes (Zhang et al., 2016) was retrieved from GEO (GSE73721). Per-gene expression levels (upper-quartile normalised TPMs) were quantitated using Salmon (Patro et al., 2017) with a quasi index (31bp k-mers) built from human coding sequences (Ensembl version 84 hg38 annotations). Gene expression levels for the RNA-seq and microarray data were merged, subject to a robust quantile normalisation (R package “preprocessCore”, weighted to be informed only the RNA-seq samples) and log2(n+1) transformed. Non-negative matrix factorisation was applied to the values from the Zhang. et. al. samples (filtered to exclude genes below the 25th expression quantile). Five meta-genes were identified using the R package “NNLM” (method=”scd”, rel.tol=-1, max.iter=100K, loss=”mkl”). Samples were hierarchically clustered by meta-gene expression level (manhattan distance, complete agglomeration), the R package “pvclust” was used to calculate approximately unbiased p-values for the clusters (nboot=100K) and leaf order was optimised using the R package “cba”.

Reverse transcription and qRT PCR

RNA was reverse transcribed using High-Capacity RNA-to-cDNA™ Kit (Thermo Fisher). Qunatitative real time PCR was performed with *Power* SYBR® Green PCR Master Mix (Thermo Fisher) on a StepOnePlus™ Real-Time PCR System. Primers used are listed in Table S5.

Flow cytometry

Co-pMG were isolated with CD11b magnetic beads (MACS®, Miltenyi Biotec) as described for the transcriptome sample preparation. Pilot experiments to detach macrophages from the tissue culture plate with accutase, in direct comparison with our previous protocol of cold 5 mM EDTA/12 mM Lidocaine (Carter et al., 2009), showed no substantive difference in surface marker levels, so accutase was used as it lifts the cells much more rapidly, thereby minimising cellular change/damage. Where relevant, macrophages were also subjected to CD11b bead treatment. Freshly harvested macrophage precursors were stained directly, after passage through a 40 µM cell strainer (BD Bioscience). The antibodies used are listed in Table S4. Isotype control antibodies used were from the same company with the same fluorophore at the same concentration (van Wilgenburg et al., 2013).

Live imaging

AH016-3 Lenti-IP-RFP-microglia were co-cultured with SFC840-03-01 cortical neurons in matrigel-coated 96-well black/clear bottom plates (Costar, 3603). RFP signal was used to visualize microglia in co-culture. Images of RFP signal and phase were taken every 5 minutes for 5 hours (2 videos/well). Microglial movement was manually tracked with ImageJ and tracks were analysed with Chemotaxis and Migration Tool Version 2.0 (Ibidi). Microglia positions were determined by marking all microglia with Image J. Manual counting and distance to next neighbour was calculated from this data with R.
To check for proinflammatory microglial morphology, co-cultures were treated with 100 ng/mL LPS imaged every 5 minutes for 17 hr respectively 20 hr. To visualise phagocytic activity, pHrodo Green zymosan yeast bioparticles (ThermoFisher, P35365) were added at 50 µg/mL. pHrodo dyes fluoresce at low pH, ie, as the phagosome is progressively acidified after uptake of the particle in microglia (Kapellos et al., 2016). Wells were imaged every 10 minutes for 5 hours.

Calcium imaging was performed with Fluo-4 Direct™ Calcium assay kit (Thermo Fisher). Cells were cultured in 100 µl microglia medium, 100 µl of assay reagent was added to the medium and cells were incubated for 1 hr at 37ºC. All medium was then removed and replaced with 200 µl Tyrode's solution supplemented with 6 mM potassium to activate the neurons. Neurons were then imaged every 3 seconds for 2 minutes. Live imaging was performed with an EVOS™ FL Auto imaging system (Thermo Fisher) with a humidified onstage incubator set to 37ºC, 5% CO_2.

Cytokine/chemokine release measurements

Proteome profilerXL (R&D systems) was used to identify candidate cytokines that are upregulated upon stimulation or differentially expressed/released between standard iPSC-derived macrophages and co-culture microglia. SFC180-01-01 pMac, or SFC180-01-01 co-pMG in co-culture with SBAD4-01 pNeurons, were

stimulated, after 3 weeks of culture, for 18 hours with 100 ng/mL LPS and 100 ng/mL IFNγ. Supernatant was then collected and applied to the proteome profiler membranes according to manufacturer's instructions. Luminescence was captured with a GeneSnap Gel documentation system (SynGene) and signal was quantified with Image Studio Lite Version 5.2 (LI-COR).

22 cytokine/chemokine targets were assayed with a ProcartaPlex™ Custom Panel (eBioscience). pNeuron were co-cultured for 2 weeks with 3 different co-pMG, meanwhile iMac from the same lines were cultured in parallel monocultures in either macrophage medium or as pMGL in microglia medium, in a 96 well plate. Cells were then stimulated with 100 ng/mL LPS and 100 ng/mL IFNγ or with medium only for 18 hr, supernatant was collected, centrifuged and analysed with multiplex beads, according to manufacturer's instructions, with a Luminex 100 Bio-Plex system (BioRad).

Supplemental References

Beers, J., Gulbranson, D., George, N., Siniscalchi, L., Jones, J., Thomson, J., and Chen, G. (2012). Passaging and colony expansion of human pluripotent stem cells by enzyme-free dissociation in chemically defined culture conditions. Nat Protocols *7*, 2029-2040.

Carter, G., Bernstone, L., Sangani, D., Bee, J., Harder, T., and James, W. (2009). HIV entry in macrophages is dependent on intact lipid rafts. Virology *386*, 192-202.

Dunning, M., Smith, M., Ritchie, M., and Tavaré, S. (2007). beadarray: R classes and methods for Illumina bead-based data. Bioinformatics (Oxford, England) *23*, 2183-2184.

Durafourt, B., Moore, C., Blain, M., and Antel, J. (2013). Isolating, culturing, and polarizing primary human adult and fetal microglia. Methods in molecular biology (Clifton, NJ) *1041*, 199-211.

Etemad, S., Zamin, R.M., Ruitenberg, M., and Filgueira, L. (2012). A novel in vitro human microglia model: Characterization of human monocyte-derived microglia. Journal of neuroscience methods *209*, 79-89.

Falcon, S., and Gentleman, R. (2007). Using GOstats to test gene lists for GO term association. Bioinformatics (Oxford, England) *23*, 257-258.

Fernandes, H.J., Hartfield, E.M., Christian, H.C., Emmanoulidou, E., Zheng, Y., Booth, H., Bogetofte, H., Lang, C., Ryan, B.J., Sardi, S.P., *et al.* (2016). ER Stress and Autophagic Perturbations Lead to Elevated Extracellular alpha-Synuclein in GBA-N370S Parkinson's iPSC-Derived Dopamine Neurons. Stem cell reports *6*, 342-356.

Kapellos, T., Taylor, L., Lee, H., Cowley, S., James, W., Iqbal, A., and Greaves, D. (2016). A novel real time imaging platform to quantify macrophage phagocytosis. Biochemical pharmacology *116*, 107-119.

Morrison, M., Klein, C., Clemann, N., Collier, D.A., Hardy, J., Heisserer, B., Cader, M.Z., Graf, M., and Kaye, J. (2015). StemBANCC: Governing Access to Material and Data in a Large Stem Cell Research Consortium. Stem cell reviews *11*, 681-687.

Patro, R., Duggal, G., Love, M., Irizarry, R., and Kingsford, C. (2017). Salmon provides fast and bias-aware quantification of transcript expression. Nature methods.

Ritchie, M., Phipson, B., Wu, D., Hu, Y., Law, C., Shi, W., and Smyth, G. (2015). limma powers differential expression analyses for RNA-sequencing and microarray studies. Nucleic acids research *43*, gkv007-e047.

Sandor, C., Robertson, P., Lang, C., Heger, A., Booth, H., Vowles, J., Witty, L., Bowden, R., Hu, M., Cowley, S., *et al.* (2017). Transcriptomic profiling of purified patient-derived dopamine neurons identifies convergent perturbations and therapeutics for Parkinson's disease. Human molecular genetics.

Shi, Y., Kirwan, P., and Livesey, F.J. (2012). Directed differentiation of human pluripotent stem cells to cerebral cortex neurons and neural networks. Nature protocols *7*, 1836-1846.

van Wilgenburg, B., Browne, C., Vowles, J., and Cowley, S. (2013). Efficient, long term production of monocyte-derived macrophages from human pluripotent stem cells under partly-defined and fully-defined conditions. PloS one *8*, e71098.

Zhang, Y., Sloan, S., Clarke, L., Caneda, C., Plaza, C., Blumenthal, P., Vogel, H., Steinberg, G., Edwards, M., Li, G., *et al.* (2016). Purification and Characterization of Progenitor and Mature Human Astrocytes Reveals Transcriptional and Functional Differences with Mouse. Neuron *89*, 37-53.
